# Supplementary material for: Impact of a short version of the CONSORT checklist for peer reviewers to improve the reporting of randomised controlled trials published in biomedical journals: study protocol for a randomised controlled trial
Source: BMJ Open. 2020 Mar 19;10(3):e035114. doi: 10.1136/bmjopen-2019-035114 (PMC7103787; doi:10.1136/bmjopen-2019-035114)
Supplement: Author's manuscript [file bmjopen-2019-035114.draft_revisions.pdf]

BMJ Open

BMJ Open is committed to open peer review. As part of this commitment we make the peer review history of every article we publish publicly available.

When an article is published we post the peer reviewers' comments and the authors' responses online. We also post the versions of the paper that were used during peer review. These are the versions that the peer review comments apply to.

The versions of the paper that follow are the versions that were submitted during the peer review process. They are not the versions of record or the final published versions. They should not be cited or distributed as the published version of this manuscript.

BMJ Open is an open access journal and the full, final, typeset and author-corrected version of record of the manuscript is available on our site with no access controls, subscription charges or pay-per-view fees (http://bmjopen.bmj.com).

If you have any questions on BMJ Open's open peer review process please email info.bmjopen@bmj.com

BMJ Open

Impact of a short version of the CONSORT checklist for peer reviewers to improve the reporting of randomised controlled trials published in biomedical journals: study protocol for a randomised controlled trial

Journal:	*BMJ Open*
Manuscript ID	bmjopen-2019-035114
Article Type:	Protocol
Date Submitted by the Author:	21-Oct-2019

Complete List of Authors:	Speich, Benjamin; University of Oxford, Centre for Statistics in Medicine, Nuffield Department of Orthopaedics, Rheumatology and Musculoskeletal Sciences<br>Schroter, Sara; BMJ Editorial,<br>Briel, Matthias; University of Basel, Basel Institute for Clinical Epidemiology and Biostatistics, Department of Clinical Research, University Hospital Basel<br>Moher, David; Ottawa Hospital Research Institute, Ottawa Methods Centre<br>Puebla, Iratxe ; PLOS ONE, PLOS ONE, Public Library of Science, San Francisco, California, United States of America and Cambridge<br>Clark, Alejandra ; PLOS One, PLOS ONE, Public Library of Science, San Francisco, California, United States of America and Cambridge<br>Maia Schlüssel, Michael; University of Oxford, Centre for Statistics in Medicine, Nuffield Department of Orthopaedics, Rheumatology and Musculoskeletal Sciences<br>Ravaud, Philippe; Universite Paris Descartes,<br>Boutron, Isabelle; Université Paris Descartes, Centre d\'épidémiologie clinique<br>Hopewell, Sally; University of Oxford, Centre for Statistics in Medicine, Nuffield Department of Orthopaedics, Rheumatology and Musculoskeletal Sciences

Keywords:	Protocols & guidelines < HEALTH SERVICES ADMINISTRATION & MANAGEMENT, Quality in health care < HEALTH SERVICES ADMINISTRATION & MANAGEMENT, MEDICAL EDUCATION & TRAINING, STATISTICS & RESEARCH METHODS

SCHOLARONE™
Manuscripts

I, the Submitting Author has the right to grant and does grant on behalf of all authors of the Work (as defined in the below author licence), an exclusive licence and/or a non-exclusive licence for contributions from authors who are: i) UK Crown employees; ii) where BMJ has agreed a CC-BY licence shall apply, and/or iii) in accordance with the terms applicable for US Federal Government officers or employees acting as part of their official duties; on a worldwide, perpetual, irrevocable, royalty-free basis to BMJ Publishing Group Ltd ("BMJ") its licensees and where the relevant Journal is co-owned by BMJ to the co-owners of the Journal, to publish the Work in this journal and any other BMJ products and to exploit all rights, as set out in our licence.

The Submitting Author accepts and understands that any supply made under these terms is made by BMJ to the Submitting Author unless you are acting as an employee on behalf of your employer or a postgraduate student of an affiliated institution which is paying any applicable article publishing charge ("APC") for Open Access articles. Where the Submitting Author wishes to make the Work available on an Open Access basis (and intends to pay the relevant APC), the terms of reuse of such Open Access shall be governed by a Creative Commons licence – details of these licences and which Creative Commons licence will apply to this Work are set out in our licence referred to above.

Other than as permitted in any relevant BMJ Author's Self Archiving Policies, I confirm this Work has not been accepted for publication elsewhere, is not being considered for publication elsewhere and does not duplicate material already published. I confirm all authors consent to publication of this Work and authorise the granting of this licence.

For peer review only

Impact of a short version of the CONSORT checklist for peer reviewers to improve the reporting of randomised controlled trials published in biomedical journals: study protocol for a randomised controlled trial

Benjamin Speich[1,2,*], Sara Schroter[3], Matthias Briel[2,4], David Moher[5,6], Iratxe Puebla[7], Alejandra Clark[7], Michael M Schlussel[1,8], Philippe Ravaud[9,10], Isabelle Boutron[9,10], Sally Hopewell[1]

[1]Centre for Statistics in Medicine, Nuffield Department of Orthopaedics, Rheumatology and Musculoskeletal Sciences, University of Oxford, Oxford, United Kingdom

[2]Basel Institute for Clinical Epidemiology and Biostatistics, Department of Clinical Research, University Hospital Basel, University of Basel, Basel, Switzerland

[3]The BMJ, London, United Kingdom

[4]Department of Health Research Methods, Evidence, and Impact, McMaster University, Hamilton, Canada

[5]Centre for Journalology, Clinical Epidemiology Program, Ottawa Hospital Research Institute, Canada

[6]School of Epidemiology and Public Health, Faculty of Medicine, University of Ottawa

[7]*PLOS ONE*, Public Library of Science, San Francisco, California, United States of America and Cambridge, United Kingdom

[8]The EQUATOR Network, Oxford, United Kingdom

[9]Centre d'Épidémiologie Clinique, Hôpital Hôtel-Dieu, Assistance Publique Hôpitaux de Paris (APHP) F-75004 Paris, France

[10]Université de Paris, CRESS, Inserm, INRA, F-75004, Paris, France

*Corresponding author:

Benjamin Speich, PhD,
Centre for Statistics in Medicine, Nuffield Department of Orthopaedics, Rheumatology and Musculoskeletal Sciences,

For peer review only
For peer review only - http://bmjopen.bmj.com/site/about/guidelines.xhtml

University of Oxford,
Windmill Road
OX3 7LD Oxford
United Kingdom
Email: benjamin.speich@ndorms.ox.ac.uk

E-mail addresses of all co-authors:

Benjamin Speich <benjamin.speich@ndorms.ox.ac.uk>

Sara Schroter <sschroter@bmj.com>

Matthias Briel <matthias.briel@usb.ch>

David Moher <dmoher@ohri.ca>

Iratxe Puebla <ipuebla@plos.org>

Alejandra Clark <aclark@plos.org>

Michael M Schlussel <michael.schlussel@ndorms.ox.ac.uk>

Philippe Ravaud <philipperavaud@gmail.com>

Isabelle Boutron <isabelle.boutron@aphp.fr>

Sally Hopewell <sally.hopewell@csm.ox.ac.uk>

Running title: CONSORT for Peer Review (CONSORT-PR)

Funding: Benjamin Speich is supported by an Advanced Postdoc.Mobility grant from the Swiss National Science Foundation (P300PB_177933). David Moher is supported by a University Research Chair, Ottawa. Michael M Schlussel is funded by Cancer Research UK. The funders had no role in designing the study and will also have no role in conducting the study, or analysing and reporting study results.

Keywords: CONSORT; reporting guidelines; randomised controlled trials as topic; peer review; meta-research

For peer review only

For peer review only - http://bmjopen.bmj.com/site/about/guidelines.xhtml

Abstract

Introduction: Transparent and accurate reporting is essential for readers to adequately interpret the results of a study. Journals can play a vital role in improving the reporting of published randomised controlled trials (RCTs). We describe an RCT to evaluate our hypothesis that asking peer reviewers to check whether the most important and poorly reported CONSORT (CONsolidated Standards for Reporting Trials) items are adequately reported, will result in higher adherence to CONSORT guidelines in published RCTs.

Methods and Analysis: Manuscripts presenting the primary results of RCTs submitted to participating journals will be randomised to either the intervention group (peer reviewers will receive a reminder and short explanation of the ten most important and poorly reported CONSORT items; they will be asked to check if these items are reported in the submitted manuscript) or a control group (usual journal practice). The primary outcome will be the mean proportion of the ten items that are adequately reported in the published articles. Peer reviewers and manuscript authors will not be informed of the study hypothesis, design, or intervention. Outcomes will be assessed in duplicate from published articles by two data extractors (at least one blinded to the intervention). We will enrol eligible manuscripts until a minimum of 83 articles per group (166 in total) are published.

Ethics and Dissemination: This pragmatic RCT was approved by the Medical Sciences Interdivisional Research Ethics Committee of the University of Oxford (R62779/RE001). If this intervention is effective, it could be implemented by all medical journals without requiring large additional resources at journal level. Findings will be disseminated through presentations in relevant conferences and peer-reviewed publications. This trial is registered on the Open Science Framework (https://osf.io/c4hn8).

For peer review only

For peer review only - http://bmjopen.bmj.com/site/about/guidelines.xhtml

Strengths and limitations of this study

- Pragmatic randomised controlled trial (RCT) with individual randomisation of real manuscripts describing RCTs submitted to a variety of journals.
- If this simple intervention is effective, it could be implemented by journals without requiring large additional resources at a journal level.
- We could not include the intervention within the journals' agreement to review letter or all peer reviewers would receive the intervention due to the automated processes of the journals' editorial systems. This risks peer reviewers potentially ignoring the separate email containing the CONSORT reminder.

For peer review only

For peer review only - http://bmjopen.bmj.com/site/about/guidelines.xhtml

Introduction

Background and rationale

There is substantial agreement that well conducted and reported randomised controlled trials (RCTs) generate the most trustworthy evidence when evaluating newly developed or existing clinical interventions.[1-3] For clinicians, scientists and decision makers, published articles are often the only way to know how a study was conducted. In order to judge the internal and external validity of RCTs, it is crucial that these articles present transparent, accurate and unbiased information about the methods and conduct of the RCT.

To improve the quality and transparency of clinical and epidemiological research, the EQUATOR (Enhancing the Quality and Transparency of Research) Network was founded in 2006.[4-9] This international network, which assists in the development of reporting guidelines and actively promotes their use, consists of methodologists, epidemiologists, reporting guideline developers, statisticians, clinicians and journal editors.

The CONSORT Statement (CONsolidated Standards of Reporting Trials) is perhaps the most prominent reporting guideline, designed to help improve the transparency and quality of reporting of RCTs.[10-12] It guides authors, peer reviewers and journal editors on the minimum information to be included in published reports of RCTs to facilitate critical judgment and interpretation of results and consists of 25 items and a flow diagram. The last update of the CONSORT Statement was published simultaneously in 10 leading medical journals in 2010[12] and currently CONSORT is endorsed by over 600 journals worldwide.[13]

Despite some improvement in reporting following the endorsement of the CONSORT Statement, there remain major reporting deficiencies in published RCTs.[3 14-20] For example, a study of 1122 RCTs indexed in PubMed in December 2012 found that many did not define the primary outcome (31%), state the sample size calculation (45%), or explain the method of

For peer review only - http://bmjopen.bmj.com/site/about/guidelines.xhtml

allocation concealment (50%).[21] This lack of transparency is a major limiting factor for readers who assess an article in order to find the answer to a specific question; it is also a major problem for scientists who perform systematic reviews and meta-analyses.

Evidence to date

Journals can play a vital role in improving the reporting of published RCTs. For example, a survey of journals' 'Instructions to Authors' in 2014 found that 63% (106 of 168) of biomedical journals mentioned CONSORT;[22] however of those journals only 38 (36%) required a completed CONSORT checklist on submission. Such implementation indicates some improvement over time compared to an assessment in 2007 when only 17 of 62 (27%) journals requested the CONSORT checklist on submission.[23] A study using interrupted time series analysis and assessing if the CONSORT checklist for reporting abstracts of RCTs had an effect on reporting quality found that results were better reported in journals which had an active editorial policy to implement the checklist.[24]

A scoping review conducted in 2017 by Blanco and colleagues summarised different interventions aimed at improving adherence to reporting guidelines.[25] They identified a number of different interventions, some of which had been evaluated at journals. However, all the interventions, except requesting submission of checklists from authors, required additional resources from the journal (e.g. internal peer review by editorial assistants or inviting an additional statistical peer-reviewer[26 27]). Therefore, it is unlikely that these interventions will be implemented in the majority of journals, especially smaller journals with limited resources. Another study found that providing authors with a web-based CONSORT tool, which combined different CONSORT extensions and provided authors with a customised checklist, did not improve reporting when used at the manuscript revision stage.[28] However, a study examining "the nature and extent of changes made to manuscripts after peer review, in relation to the

For peer review only - http://bmjopen.bmj.com/site/about/guidelines.xhtml

reporting of methodological aspects of RCTs" and "the type of changes requested by peer reviewers" found that peer review did lead to some improvement in reporting.[26]

The role of peer reviewers and expectations of them is varied.[29] While CONSORT checklists are sometimes available for peer reviewers to check, they are not typically instructed to assess this information as part of their review and there have been no studies evaluating the effect of asking them to do this. We plan to evaluate the impact of giving peer reviewers a short version of the CONSORT checklist together with a brief explanation of the items and asking them to check if they are adequately reported.

Methods

Objective

The objective of this study is to evaluate the impact of giving peer reviewers, during the standard peer review process, a short version of the CONSORT checklist (C-short) together with a brief explanation of the items and asking them to check if they are adequately reported in the manuscript.

Study design

This study is a multicentre RCT with submitted manuscripts as the unit of randomisation (Figure 1; allocation ratio 1:1).

Study setting and eligibility criteria

The population will be defined on two levels: included journals and included manuscripts.

<u>Inclusion criteria for journals:</u>

For peer review only - http://bmjopen.bmj.com/site/about/guidelines.xhtml

Included journals must: i) endorse the CONSORT Statement by mentioning it in the journals' Instruction to Authors; ii) have published primary results of at least five RCTs in 2017 (identified using a PubMed search). To be efficient, we plan to contact (via email) the editors of eligible journals from specific publishers (e.g. BMJ Publishing Group; Public Library of Science [PLOS]) instead of separate journals. A description of the requirements for participation and a short summary information sheet will be included as part of the email invitation sent to journal editors. If a journal is eligible, and the editor agrees to take part, the editor will need to provide access to their editorial system (e.g. ScholarOne, Editorial Manager) to enable the external researcher (BS) to screen and randomise eligible manuscripts. In cases where this is not possible, we will explore with individual journals if it would be possible to grant limited access (e.g. only rights to screen studies) or to handle the different steps without access to the editorial system (e.g. screening through automated reports; intervention provided by a journal staff member) and that the emails for the intervention would be sent by a member of the editorial team.

<u>Inclusion criteria for manuscripts</u>

- All new manuscript submissions reporting the primary results of RCTs, which the journal editor has decided to send out for external peer review. Since the 10 chosen CONSORT checklist items (C-short) are applicable to different study designs, we will include all manuscripts reporting the primary results of RCTs regardless of study design (e.g. parallel group trial, cluster trial, superiority trial, non-inferiority/equivalence trials).

<u>Exclusion criteria for manuscripts</u>

- Manuscripts clearly presenting secondary trial results, additional time points, economic analyses, or any other analyses.
- Manuscripts which are clearly labelled as a pilot or feasibility study or animal studies.
- Manuscripts not sent for peer review.

For peer review only - http://bmjopen.bmj.com/site/about/guidelines.xhtml

Details of journal manuscript submission and peer review processes, including consent and potential confidentiality issues will be discussed in detail with each journal by teleconference and/or face to face prior to the journal agreeing to take part to ensure that randomisation of manuscripts is feasible.

In participating journals, the external researcher (BS) will check at least twice a week (by screening automated submission lists) all research manuscripts that are sent out for external peer review. As soon as the first invited peer reviewer accepts the invitation to review, the manuscript will be randomised to the intervention or control arm (see "Randomisation" for more details). It is possible that this process might be slightly different amongst different included journals (e.g. that team members of a journal might be involved in the screening if limited or no access to the journal's editorial system is granted).

Interventions

<u>Control group: Usual practice</u>

After accepting to review a manuscript, peer reviewers will receive the automated, journal specific standard email with general information as per each journal's usual practice (e.g. where to access the manuscript, date the peer review report is due).

<u>Intervention group: C-short plus usual practice</u>

After accepting to review a manuscript, peer reviewers will receive the automated, journal specific standard email with general information (identical to control group). In addition, peer reviewers will receive an additional email from the editorial office that includes a short version of the CONSORT checklist (C-short) together with a brief explanation of the items either as a table within the email or as an attachment - based on the preferences and possibilities of the journal (Table 1, appendix 1). Peer reviewers will be asked to check whether the items in the

For peer review only - http://bmjopen.bmj.com/site/about/guidelines.xhtml

C-short checklist are addressed in the manuscript and to request authors to include these items if they are not adequately reported. This second email (see appendix 1), containing the C-short checklist together with a brief explanation, is not generated automatically within the existing journal editorial systems (e.g. ScholarOne or Editorial Manager); it will be sent manually by a researcher (BS) from the journal's editorial system or by a member of the journal's staff. In both cases the email will appear to have come from the editorial office (not the researcher).

<u>Development of the C-short checklist and explanation of items</u>

For the development of C-short we chose the 10 most important and poorly reported CONSORT items as identified by a group of CONSORT experts in a previous study conducted by Hopewell and colleagues.[28] The selection of the items was based on expert opinion and empirical evidence whenever available.[28] In addition, to enable peer reviewers to better understand the items, we added a short explanation for each of the 10 items. These short explanations were extracted and amended from the CONSORT explanation and elaboration paper[10] and from COBWEB which is an online writing aid tool.[30] The short explanation was discussed and adapted by the scientific committee.

Outcomes

<u>Primary outcome</u>

The primary outcome of this study will be the difference in the mean proportion of adequately reported C-short items in published articles between the two groups.

<u>Secondary outcomes</u>

Secondary outcomes will include the following:

- Mean proportion of adequately reported C-short items in published articles considering each item separately.

For peer review only - http://bmjopen.bmj.com/site/about/guidelines.xhtml

For peer review only

- Difference in mean proportion of adequately reported C-short items in published articles considering each sub-item (see “Assessment of outcomes”) as a separate item.
- Time from assigning an editor to the first decision (as communicated to the author after the first round of peer-review).
- Proportion of manuscripts rejected after the first round of peer review.
- Proportion of manuscripts that will be published in the journal under study.

Additional outcomes:

- Exploratory analysis of available peer reviewer comments (i.e. any references to CONSORT).

For journals where peer reviewers’ comments are subsequently published alongside the published article, we will examine the peer reviewers’ comments for any reference to CONSORT and trial reporting. We will contact those journals which do not make peer reviewers’ comments publicly available, to see if reviews could be provided for such analyses under the condition that only anonymised data will be published.

Assessment of outcomes:

The outcomes will be assessed independently by two (blinded or at least partially blinded; see “blinding”) outcome assessors with expertise in the design and reporting of clinical trials. Any disagreement will be resolved by consensus or if necessary by consulting a third assessor. To ensure consistency between reviewers, we will first pilot the data extraction form; any disparities in the interpretation will be discussed and the data extraction form will be modified accordingly.

Adequate reporting of items will be assessed in duplicate from published full-text publications following the same instructions as provided by the CONSORT C-short checklist.[10] The following checklist items have, due to their complexity, sub-items which will be extracted

For peer review only - http://bmjopen.bmj.com/site/about/guidelines.xhtml

separately. The sub-items are highlighted in the short explanation of the intervention (see Table 1 and appendix 1):

- Outcomes (item 6a): (i) Define primary outcome, (ii) how it was measured, (iii) at what time point, and (iv) the analysis metric (e.g. change from baseline, final value).
- Sample size (item 7a): (i) The estimated outcomes in each group, (ii) the α (type I) error level, (iii) the statistical power (or the β (type II) error level), (iv) for continuous outcomes, the standard deviation of the measurements
- Blinding (item 11a): Is the blinding status clear for the following persons: (i) Healthcare provider, (ii) patients, and (iii) outcome assessors.
- Funding (item 25): (i) The funding source, and (ii) the role of funder in the design, conduct, analysis, and reporting.

All items will be judged as either “yes” meaning adequately reported, “no” meaning not adequately reported or not reported at all, or “NA” meaning that this sub-item is not applicable for this RCT. Items with different sub-items will only be judged as adequately reported if all relevant sub-items were adequately reported.

The outcomes “time from assigning an editor to the first decision”, “proportion of manuscripts rejected after the first round of peer-review”, and “proportion of manuscripts that will be published in the journal under study” will be extracted directly from the journal’s editorial system or provided by the journal.

Participant timeline

The overview of the study schedule, including enrolment, intervention and assessments is presented in Table 2.

For peer review only - http://bmjopen.bmj.com/site/about/guidelines.xhtml

Sample size

For the sample size calculation, we hypothesised in a first scenario (Table 3) that the intervention C-short will result in a 25% relative increase in adequate reporting compared to the control (meaning that 70% of items will be adequately reported in the intervention group and 56% in the control group). This is based on a proportion of adequate reporting of 0.56 for the 10 most important and poorly reported items found in the control group of a previous study (meaning that a mean of 56% of the 10 most important and poorly reported items were reported).[28] The standard deviation (SD) in the same study was 0.23. However, we calculated our sample size to account for a slightly larger variability in our data (SD = 0.25). To demonstrate a significant difference with a power of 90% and a type 1 error at 5%, a total of 136 published articles will be required in this scenario (68 per treatment arm; based on a two sided t-test).

Two authors of this protocol, working for *PLOS ONE* (IP and AC), one of the participating journals, pointed out that 3 out of the 10 assessed items (i.e. item “Registration”, “Protocol”, and “Funding”) should always be implemented in submissions to their journal given their policy requirements for clinical trials. Assuming that this journal will recruit a high proportion of manuscripts, and that also other journals might update their templates, we increased the sample size in a second scenario, in which all these 3 items would have an overall adherence of 90% in the control arm (Table 3). This would entail an overall baseline adherence with the 10 C-short items of 71%. Based on a two sided t-test, a sample size of 166 (83 per treatment arm) will have a power of 80% to find a 15% relative increase (71% adherence in control group; 82% adherence in intervention group; SD = 0.25; a type 1 error at 5%).

Since the final sample size will be based on the number of articles published, rather than on the number of manuscripts randomised, eligible manuscripts will be randomised until 83 articles are published in each arm (resulting in no less than 166 articles), to avoid loss of power due to potential imbalance between arms. Recruitment will be stopped as soon as both arms

For peer review only - http://bmjopen.bmj.com/site/about/guidelines.xhtml

reach the sample size of 83. After recruitment has stopped we will wait three months so that manuscripts, which are still in production, can be published. Manuscripts which are published after the three month period will be excluded

Randomisation and blinding

Manuscripts meeting the eligibility criteria and sent out for external peer review by the journals will be randomised into one of the two groups (allocation 1:1). The randomisation list will be created by the Study-Randomizer© system[31] using random block sizes between 2 and 8 and stratified by journal. As soon as the first peer reviewer accepts the invitation, the manuscript will be included and randomised to one of the two study arms. One of the investigators (BS) will log onto the Study-Randomizer© system[31] and enter the study identification number (ID; provided by the journal), the study title, and the journal the study was submitted to. Subsequently, all additional peer reviewers accepting the invitation to review the same manuscript will receive the same group assignment as the first peer reviewer.

Authors will be blinded to the intervention. Editors will not be actively informed about the randomisation (possible exception listed under “Interventions”). To avoid potential bias, peer reviewers and manuscript authors will not be informed of the study hypothesis, design and intervention.

Outcomes will be assessed in duplicate (see “Assessment of outcomes”). At least one outcome assessor will be blinded. Due to restricted resources the investigator conducting the randomisation (BS) might be involved in the data-extraction from published manuscripts.

Data analysis

For peer review only - http://bmjopen.bmj.com/site/about/guidelines.xhtml

All quantitative variables will be described using means and standard deviations, or medians and interquartile ranges in case severe departures from a normal distribution are identified. Data distributions will be inspected visually (i.e. by histograms) instead of performing formal statistical tests for normality. Categorical variables will be described using frequencies and percentages. For the primary and secondary outcomes, we will estimate the mean difference between the two groups and report them with respective 95% confidence intervals. No interim analysis will be conducted.

Populations of analysis

The main population for analysis will be all manuscripts randomised and accepted for publication in the participating journals. In contrast to RCTs conducted with patients, where losses to follow-up need to be carefully considered (e.g. multiple imputation of missing data), we are only interested in the reporting adherence of RCTs that are published. Hence we will exclude randomised manuscripts that were not published from the main analysis. All outcomes will be calculated based on the main population. The secondary outcome "Time to the first decision", will additionally be calculated considering all randomised manuscripts (including the ones which were not published). For all analyses a p-value of 0.05 (5% significance level) will be used to indicate statistical significance. Exact p-values will be presented up to three decimal places. We anticipate there will be no missing data in this study, neither at the individual C-short items, nor at the manuscript level. This is due to the study design, which will include only the randomised manuscripts that are accepted for publication. We will analyse if the rate of manuscripts rejected after the first round of peer-review and if the proportion of manuscripts that will be published differentiate amongst the two study arms (both secondary results).

For peer review only

For peer review only - http://bmjopen.bmj.com/site/about/guidelines.xhtml

Analysis of primary endpoint

The effect of the intervention will be estimated as the mean difference in the proportion of C-short items adequately reported between the study arms. If the data on the primary outcome is normally distributed, groups will be compared using an unpaired Student's t-test. If the data is not normally distributed, comparisons will be performed using a non-parametric equivalent test (i.e. Wilcoxon-Mann-Whitney test).

Analysis of secondary endpoints

To investigate the effect of the intervention on the secondary outcomes, mean differences with respective 95% confidence intervals will be reported. If normality is not observed for any of the continuous secondary outcomes, the same strategy adopted for the primary outcome (use of a non-parametric equivalent to the Student's *t*-test) will be used.

Pre-specified subgroup analysis

No formal subgroup comparative analysis is planned for the primary or secondary outcomes. However, the effect of the intervention on the primary outcome within subgroups will be presented using forest plots to visually examine whether it may differ according to some variables, such as: (1) Journals that actively implement the CONSORT Statement (defined as requiring authors to submit a completed CONSORT checklist alongside their manuscript) vs. journals that are not actively implementing the CONSORT Statement; (2) sample size of included RCTs (n < 100 vs. n ≥ 100); and (3) impact factor (<5, 5.1-10; >10) as there is evidence that higher impact factor as well as higher sample size are associated with higher adherence to reporting guidelines.[32] These analyses will be exploratory, with the aim of supporting new hypothesis generation, rather than being conclusive.

For peer review only - http://bmjopen.bmj.com/site/about/guidelines.xhtml

Data management and confidentiality

Outcomes from publications will be assessed and extracted in duplicate. Since this information is not confidential, we will use freely available online forms (e.g. Google forms) for data extraction from published RCTs. Data entered will be validated for completeness.

Data from the journal's editorial system (e.g. title of manuscript, first author, randomisation ID, journal, date when manuscript was assigned to an editor, date when the final decision was made, final editorial decision, number of peer reviewers who reviewed the manuscript, the peer review reports [if available]) will be extracted (by BS or a member of the journal's staff), anonymised and entered in password protected files which are saved on a server from the University of Oxford. Data will be managed and curated according to University of Oxford regulations, which includes regular back-up (on a daily basis) of the virtual drives where the data are stored. No auditing or data monitoring is planned (as outcomes are directly extracted from journal's editorial system or in duplicate from published RCTs).

The raw data extracted from the included published manuscripts can be made openly accessible in an anonymised way (i.e. giving the included RCT a number instead of identifying them). Derived/aggregated data, including anonymised information generated from the journal's editorial system, will be stored and made available to the research community when the project ends (see also "Publication policy and access to data"). Where appropriate, the researcher who has access to the journal's editorial system (BS) and anyone else who will see the identifiable data will sign a confidentially agreement with the participating journals, confirming that they will not share identifiable data with any other party. Publishers such as the BMJ state in their Company Privacy Statement that reviews and manuscripts may be used for quality improvement purposes and that is the nature of this research. Furthermore, peer reviewers for all BMJ journals receive the following statement in their invitation letter "*We are constantly trying to find ways of improving the peer review system and have an ongoing programme of research. If you do not wish your review entered into a study please let us know by emailing […] as soon as possible*."

For peer review only

For peer review only - http://bmjopen.bmj.com/site/about/guidelines.xhtml

Trial registration

This trial was denied registration on ClinicalTrials.gov as the study is not a clinical study that assesses a health outcome in human subjects. Instead we registered the trial on the Open Science Framework (https://osf.io/c4hn8). The first manuscript was randomised in July 2019. We expect that recruitment will be finished in summer 2021.

Patient and public involvement

Given the specific study topic, the steering committee agreed that patient or public involvement is not needed for this study.

Discussion

RCTs are the current gold standard for evaluating any new intervention in evidence-based medicine. Unfortunately, not all RCTs are of high quality. In fact, there are several well-known shortcomings with respect to reporting.[3 14-19] It is important to note that adhering to the CONSORT Statement does not mean that the study is of high quality. However, reporting all items from the CONSORT checklist will enable readers to adequately judge the quality of RCTs.

In this RCT we will test if a simple intervention in the form of asking peer reviewers to check whether selected CONSORT items are adequately addressed will increase the proportion of reporting completeness in the published RCTs in the participating journals. A multicentre parallel arm RCT with randomisation at the individual manuscript level was chosen instead of a cluster RCT because the risk of "contamination" at journal level was judged as low as the

For peer review only - http://bmjopen.bmj.com/site/about/guidelines.xhtml

intervention will be implemented by an external researcher (i.e. BS) or a member of the journal staff (e.g. personnel from Editorial services). The likelihood of contamination due to peer reviewers being invited to assess several RCTs and therefore becoming exposed to both intervention arms was judged small and therefore we do not plan to adjust for clustering by journal. Originally we planned to implement the intervention within the original instruction to peer reviewer email which is sent out as soon as a peer reviewer accepts the invitation from the journal. However, as these emails are sent automatically by the journal's editorial system we would have needed to modify the software from each journal to make sure that only half of the manuscripts administered the intervention. After our first discussion with journal editors and journal staff, we realised that this approach is not feasible and therefore decided to implement the intervention in the form of a separate email. We intended to conduct this RCT in a pragmatic way so that results "would also be relevant to […] people who decide whether to implement the intervention on the basis of its results".[33] Hence we chose to assess outcomes from published articles and not from manuscripts after the first round of revisions. Ideally, the full impact of the intervention would also be measured including all versions of randomised manuscripts in the final statistical analysis. However, due to confidentiality issues and limited resources we will not be able to evaluate manuscript versions prior to publication.

A selection of CONSORT items was chosen instead of the entire CONSORT checklist as we did not want to put too high a burden on peer reviewers, which could increase the risk that peer reviewers ignore our reminder.

Should the proposed intervention be successful in improving the reporting quality of published RCTs, as measured by the adherence to CONSORT, the intervention could be implemented at the journal level without requiring a large amount of additional resources. In addition, very

For peer review only - http://bmjopen.bmj.com/site/about/guidelines.xhtml

similar interventions for other article types (e.g. systematic reviews, trial protocols) and corresponding guidelines (e.g. PRISMA, SPIRIT) could be easily implemented too.

Authors' contributions

SH, BS, IB, MB, DM, and PR had the study idea and designed the study. SS, IP and AC provided expertise to ensure implementation at the journal level was possible. MMS was responsible for statistical aspects, including the sample size calculation and the data analysis plan. BS and SH wrote the first draft of the study protocol. All authors critically revised the protocol and approved the final version.

Roles and responsibilities

The principal investigator (BS) is responsible for the preparation and the revisions of the study protocol, organising meetings of the steering committee, recruiting and randomising eligible manuscripts as well as the publication of study reports. The steering committee (IB, MB, SH, DM, PR, BS, MMS, and SS) is responsible for revising the protocol, defining and validating the additional short explanation for each CONSORT item, advising on study implementation, and for publishing the results of this study. MMS is responsible for the sample size calculation and the statistical analyses.

Ethical approval

Ethical approval has been obtained from the Medical Sciences Interdivisional Research Ethics Committee of the University of Oxford (R62779/RE001). The original approved study protocol is available in Appendix 2. The WHO Trial Registration Data Set is available in Appendix 3.

Competing interests

SS is employed by the British Medical Journal (BMJ). IP and AC are employed by the Public Library of Science (PLOS). DM, SH, and IB are members of the CONSORT executive and

For peer review only - http://bmjopen.bmj.com/site/about/guidelines.xhtml

For peer review only

authors of the CONSORT 2010 Statement. DM and PR are members of the EQUATOR network steering group. MMS is a meta-researcher and reporting guideline developer, enthusiast, and disseminators, he may therefore overestimate the importance of this project. All authors have declared that no other competing interests exist.

Publication policy and access to data

The results from this study will be published in a peer reviewed journal irrespective of the study results. Authorship of publications will be granted according to the criteria of the International Committee of Medical Journal Editors (ICMJE). We plan to make the anonymised dataset, including the data from the published articles, available as a supplementary file of the main publication.

For peer review only

References

1. Duley L, Antman K, Arena J, et al. Specific barriers to the conduct of randomized trials. *Clin Trials* 2008;5(1):40-8. doi: 10.1177/1740774507087704
2. Collins R, MacMahon S. Reliable assessment of the effects of treatment on mortality and major morbidity, I: clinical trials. *Lancet* 2001;357(9253):373-80. doi: 10.1016/S0140-6736(00)03651-5
3. Chalmers I. Unbiased, relevant, and reliable assessments in health care: important progress during the past century, but plenty of scope for doing better. *BMJ* 1998;317(7167):1167-8.
4. Altman DG, Simera I, Hoey J, et al. EQUATOR: reporting guidelines for health research. *Lancet* 2008;371(9619):1149-50. doi: 10.1016/S0140-6736(08)60505-X
5. Simera I. EQUATOR Network collates resources for good research. *BMJ* 2008;337:a2471. doi: 10.1136/bmj.a2471
6. Simera I, Altman DG. Writing a research article that is "fit for purpose": EQUATOR Network and reporting guidelines. *Evid Based Med* 2009;14(5):132-4. doi: 10.1136/ebm.14.5.132
7. Simera I, Altman DG, Moher D, et al. Guidelines for reporting health research: the EQUATOR network's survey of guideline authors. *PLoS Med* 2008;5(6):e139. doi: 10.1371/journal.pmed.0050139
8. Simera I, Moher D, Hirst A, et al. Transparent and accurate reporting increases reliability, utility, and impact of your research: reporting guidelines and the EQUATOR Network. *BMC Med* 2010;8:24. doi: 10.1186/1741-7015-8-24
9. Simera I, Moher D, Hoey J, et al. The EQUATOR Network and reporting guidelines: Helping to achieve high standards in reporting health research studies. *Maturitas* 2009;63(1):4-6. doi: 10.1016/j.maturitas.2009.03.011
10. Moher D, Hopewell S, Schulz KF, et al. CONSORT 2010 explanation and elaboration: updated guidelines for reporting parallel group randomised trials. *BMJ* 2010;340:c869. doi: 10.1136/bmj.c869
11. Schulz KF, Altman DG, Moher D, et al. CONSORT 2010 Statement: updated guidelines for reporting parallel group randomised trials. *BMC Med* 2010;8:18. doi: 10.1186/1741-7015-8-18
12. Schulz KF, Altman DG, Moher D, et al. CONSORT 2010 statement: updated guidelines for reporting parallel group randomised trials. *BMJ* 2010;340:c332. doi: 10.1136/bmj.c332
13. CONSORT Transparent Reporting of Trials. Endorsers. Journals and Organizations. http://www.consort-statement.org/about-consort/endorsers [accessed: 27 February 2019]
14. Chan AW, Hrobjartsson A, Haahr MT, et al. Empirical evidence for selective reporting of outcomes in randomized trials: comparison of protocols to published articles. *JAMA* 2004;291(20):2457-65. doi: 10.1001/jama.291.20.2457
15. Chalmers I, Glasziou P. Avoidable waste in the production and reporting of research evidence. *Lancet* 2009;374(9683):86-9. doi: 10.1016/S0140-6736(09)60329-9
16. Ioannidis JP, Lau J. Completeness of safety reporting in randomized trials: an evaluation of 7 medical areas. *JAMA* 2001;285(4):437-43.
17. Glasziou P, Meats E, Heneghan C, et al. What is missing from descriptions of treatment in trials and reviews? *BMJ* 2008;336(7659):1472-4. doi: 10.1136/bmj.39590.732037.47 [published Online First: 2008/06/28]
18. Boutron I, Dutton S, Ravaud P, et al. Reporting and interpretation of randomized controlled trials with statistically nonsignificant results for primary outcomes. *JAMA* 2010;303(20):2058-64. doi: 10.1001/jama.2010.651

For peer review only - http://bmjopen.bmj.com/site/about/guidelines.xhtml

19. Hopewell S, Dutton S, Yu LM, et al. The quality of reports of randomised trials in 2000 and 2006: comparative study of articles indexed in PubMed. *BMJ* 2010;340:c723. doi: 10.1136/bmj.c723
20. Turner L, Shamseer L, Altman DG, et al. Does use of the CONSORT Statement impact the completeness of reporting of randomised controlled trials published in medical journals? A Cochrane review. *Syst Rev* 2012;1:60. doi: 10.1186/2046-4053-1-60
21. Odutayo A, Emdin CA, Hsiao AJ, et al. Association between trial registration and positive study findings: cross sectional study (Epidemiological Study of Randomized Trials-ESORT). *BMJ* 2017;356:j917. doi: 10.1136/bmj.j917 [published Online First: 2017/03/16]
22. Shamseer L, Hopewell S, Altman DG, et al. Update on the endorsement of CONSORT by high impact factor journals: a survey of journal "Instructions to Authors" in 2014. *Trials* 2016;17(1):301. doi: 10.1186/s13063-016-1408-z [published Online First: 2016/06/28]
23. Hopewell S, Altman DG, Moher D, et al. Endorsement of the CONSORT Statement by high impact factor medical journals: a survey of journal editors and journal 'Instructions to Authors'. *Trials* 2008;9:20. doi: 10.1186/1745-6215-9-20 [published Online First: 2008/04/22]
24. Hopewell S, Ravaud P, Baron G, et al. Effect of editors' implementation of CONSORT guidelines on the reporting of abstracts in high impact medical journals: interrupted time series analysis. *BMJ* 2012;344:e4178. doi: 10.1136/bmj.e4178 [published Online First: 2012/06/26]
25. Blanco D, Altman D, Moher D, et al. Scoping review on interventions to improve adherence to reporting guidelines in health research. *BMJ Open* 2019;9(5):e026589. doi: 10.1136/bmjopen-2018-026589 [published Online First: 2019/05/12]
26. Hopewell S, Collins GS, Boutron I, et al. Impact of peer review on reports of randomised trials published in open peer review journals: retrospective before and after study. *BMJ* 2014;349:g4145. doi: 10.1136/bmj.g4145
27. Cobo E, Selva-O'Callagham A, Ribera JM, et al. Statistical reviewers improve reporting in biomedical articles: a randomized trial. *PLoS One* 2007;2(3):e332. doi: 10.1371/journal.pone.0000332 [published Online First: 2007/03/29]
28. Hopewell S, Boutron I, Altman DG, et al. Impact of a web-based tool (WebCONSORT) to improve the reporting of randomised trials: results of a randomised controlled trial. *BMC Med* 2016;14(1):199. doi: 10.1186/s12916-016-0736-x
29. Glonti K, Cauchi D, Cobo E, et al. A scoping review on the roles and tasks of peer reviewers in the manuscript review process in biomedical journals. *BMC Med* 2019;17(1):118. doi: 10.1186/s12916-019-1347-0 [published Online First: 2019/06/21]
30. Barnes C, Boutron I, Giraudeau B, et al. Impact of an online writing aid tool for writing a randomized trial report: the COBWEB (Consort-based WEB tool) randomized controlled trial. *BMC Med* 2015;13:221. doi: 10.1186/s12916-015-0460-y [published Online First: 2015/09/16]
31. Study Randomizer. https://studyrandomizer.com/ [accessed: 8. July 2019].
32. Jin Y, Sanger N, Shams I, et al. Does the medical literature remain inadequately described despite having reporting guidelines for 21 years? - A systematic review of reviews: an update. *Journal of multidisciplinary healthcare* 2018;11:495-510. doi: 10.2147/jmdh.S155103 [published Online First: 2018/10/13]
33. Loudon K, Treweek S, Sullivan F, et al. The PRECIS-2 tool: designing trials that are fit for purpose. *BMJ* 2015;350:h2147. doi: 10.1136/bmj.h2147 [published Online First: 2015/05/10]

For peer review only - http://bmjopen.bmj.com/site/about/guidelines.xhtml

Figure legend

Figure 1: Study flowchart

For peer review only

For peer review only - http://bmjopen.bmj.com/site/about/guidelines.xhtml

Table 1: The ten most important and poorly reported CONSORT items as defined by a group of experts on the CONSORT statement.[28] For better understanding key features were summarised within a short explanation (extracted from the CONSORT explanation and elaboration paper[10] as well as from the COBWEB tool[30]).

Item	Section	CONSORT item	Short explanation
1	Outcomes (6a)	Completely defined pre-specified primary outcome measure, including how and when it was assessed	Is it clear (1) what the primary outcome is (usually the one used in the sample size calculation), (2) how it was measured (if relevant; e.g. which score used), (3) at what time point, and (4) what the analysis metric was (e.g. change from baseline, final value)?
2	Sample size (7a)	How sample size was determined	Is there a clear description of how the sample size was determined, including (1) the estimated outcomes in each group; (2) the α (type I) error level; (3) the statistical power (or the β (type II) error level); and (4) for continuous outcomes, the standard deviation of the measurements?
3	Sequence generation (8a)	Method used to generate random allocation sequence	Does the description make it clear if the "assigned intervention is determined by a chance process and cannot be predicted"?
4	Allocation concealment (9)	Mechanism used to implement random allocation sequence (such as sequentially numbered containers), describing any steps taken to conceal the sequence until interventions were assigned	Is it clear how the care provider enrolling participants was made ignorant of the next assignment in the sequence (different from blinding)? Possible methods can rely on centralised or "third-party" assignment (i.e., use of a central telephone randomisation system, automated assignment system, sealed containers).
5	Blinding (11a)	If done, who was blinded after assignment to interventions (for example, participants, care providers, those assessing outcomes)	Is it clear if (1) healthcare providers, (2) patients, and (3) outcome assessors are blinded to the intervention? General terms such as "double-blind" without further specifications should be avoided.
6	Outcomes and estimation (17a/b)	For the primary outcome, results for each group, and the estimated effect size and its precision (such as 95% confidence intervals)	Is the estimated effect size and its precision (such as standard deviation or 95% confidence intervals) for each treatment arm reported? When the primary outcome is binary, both the relative effect (risk ratio, relative risk) or odds ratio) and the absolute effect (risk difference) should be reported with confidence intervals.
7	Harms (19)	All-important harms or unintended effects in each group	Is the number of affected persons in each group, the severity grade (if relevant) and the absolute risk (e.g. frequency of incidence) reported? Are the number of serious, life threatening events and deaths reported? If no adverse event occurred this should be clearly stated.
8	Registration (23)	Registration number and name of trial registry	Is the registry and the registration number reported? If the trial was not registered, it should be explained why.
9	Protocol (24)	Where trial protocol can be accessed	Is it stated where the trial protocol can be assessed (e.g. published, supplementary file, repository, directly from author, confidential and therefore not available)?
10	Funding (25)	Sources of funding and other support (such as supply of drugs) and role of funders	Are (1) the funding sources, and (2) the role of the funder(s) described?

For peer review only - http://bmjopen.bmj.com/site/about/guidelines.xhtml

Table 2: Study schedule

	Enrolment	Allocation and intervention	Intervention	Post-intervention	
Time-point	*Studies which are sent out for peer-review*	*After first peer-reviewer accepts invitation*	*Whenever an additional peer-reviewer accepts invitation*	*First decision by journal*	*Published manuscripts*
Eligibility screen	X				
Allocation		X			
Intervention:					
C-short + usual care		X	X		
Usual care		X	X		
Assessment of trial characteristics:					
Funding source					X
Study centres (single centre or multicentre)					X
Sample size					X
Study design (e.g. parallel arm, crossover)					X
Hypothesis (e.g. superiority, non-inferiority)					X
Medical field					X
Intervention tested					X
Number of trial arms					X
Number of peer-reviewers					X
Journal which published the manuscript					X
Number of journals requesting CONSORT adherence (submission of checklist mandatory)					X
Assessment of outcomes:					
Time from assigning an academic editor until the first decision				X	
Proportion of manuscripts directly rejected after the first round of peer-review				X	
Proportion of manuscripts that will be published in the journal under study					X
Adherence to CONSORT items and sub-items					X

For peer review only - http://bmjopen.bmj.com/site/about/guidelines.xhtml

632 **Table 3:** Assumptions for sample size calculations in two different scenarios.

Item	CONSORT item	Scenario 1. Adequate reporting as published in WebCONSORT [28]	Scenario 2. Adapted from Scenario 1
1	Outcomes (6a)	77% (79 of 103)	77% (79 of 103)
2	Sample size (7a)	83% (85 of 103)	83% (85 of 103)
3	Sequence generation (8a)	76% (78 of 103)	76% (78 of 103)
4	Allocation concealment (9)	55% (57 of 103)	55% (57 of 103)
5	Blinding (11a)	35% (36 of 103)	35% (36 of 103)
6	Outcomes and estimation (17a	44% (45 of 103)	44% (45 of 103)
7	Harms (19)	71% (73 of 103)	71% (73 of 103)
8	Registration (23)	69% (71 of 103)	90%
9	Protocol (24)	19% (20 of 103)	90%
10	Funding (25)	34% (35 of 103)	90%
**Overall**		**56%**	**71%**

633 Abbreviation: CONSORT= CONsolidated Standards for Reporting Trials

634

For peer review only

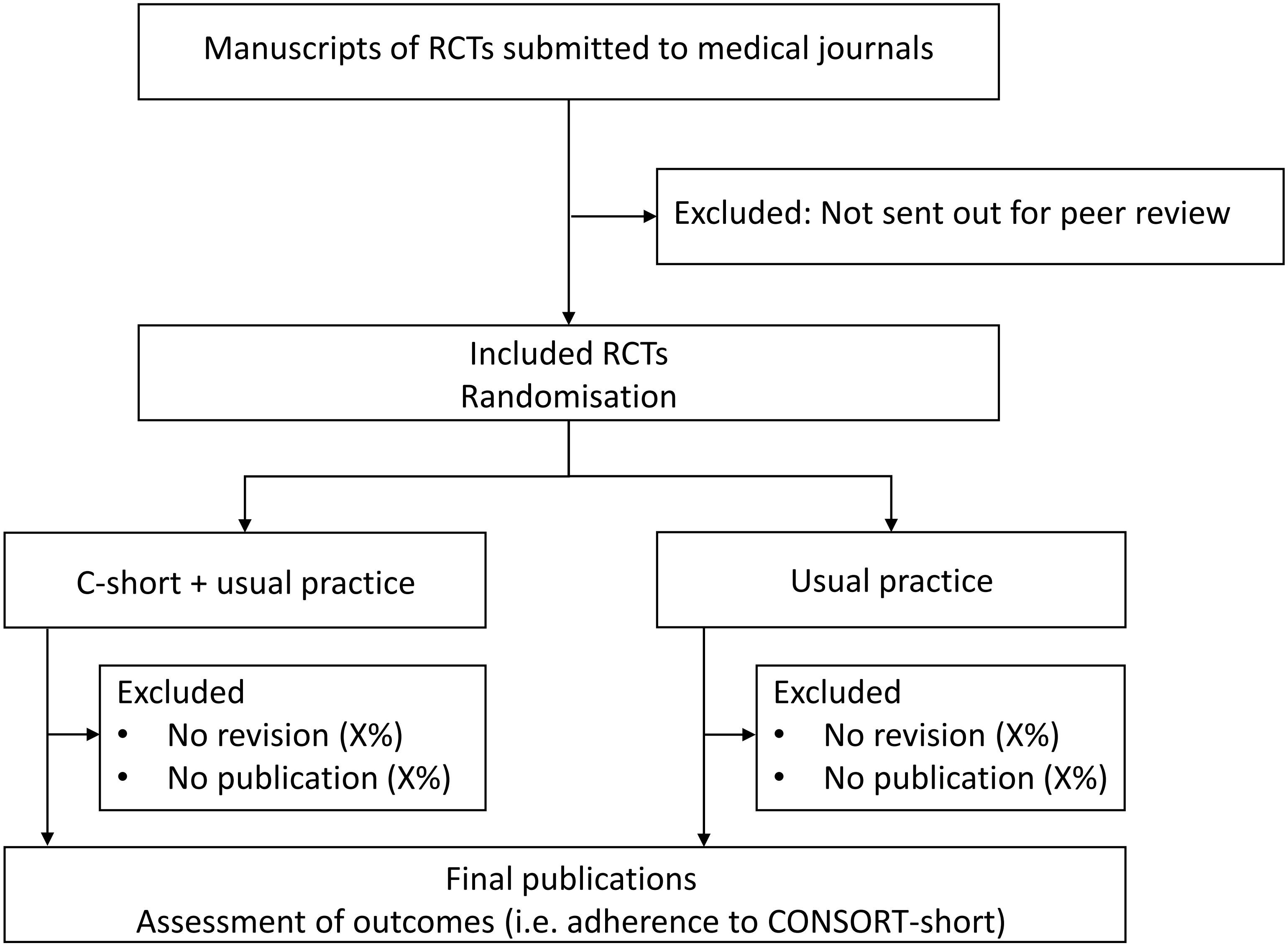
Manuscripts of RCTs submitted to medical journals
Excluded: Not sent out for peer review
Included RCTs
Randomisation
C-short + usual practice
Usual practice
Excluded
• No revision (X%)
• No publication (X%)
Excluded
• No revision (X%)
• No publication (X%)
Final publications
Assessment of outcomes (i.e. adherence to CONSORT-short)

Impact of a short version of the CONSORT checklist for peer reviewers to improve the reporting of randomised controlled trials published in biomedical journals: study protocol for a randomised controlled trial

Benjamin Speich, Sara Schroter, Matthias Briel, David Moher, Iratxe Puebla, Alejandra Clark, Michael M Schlussel, Philippe Ravaud, Isabelle Boutron, Sally Hopewell

Appendix

Appendix 1: Example of the email which will be sent out in the intervention arm (C-Short). The exact wording might be slightly adapted according to the journal preferences.
Page 2

Appendix 2: Original study protocol as it was approved by the Medical Sciences Interdivisional Research Ethics Committee of the University of Oxford (R62779/RE001).
Page 3

Appendix 3: WHO Trial Registration Data Set (Version 1.3.1)
Page 27

For peer review only - http://bmjopen.bmj.com/site/about/guidelines.xhtml

Appendix 1: Example of the email which will be sent out in the intervention arm (C-Short). The exact wording might be slightly adapted according to the journal preferences.

Dear **Title, Name**,

We thank you for accepting to peer-review a manuscript for **journal name**. As we are trying to improve the reporting for randomised controlled trials according to the CONSORT guidelines, we would like to ask if you could check whether the following most important and poorly reported items are adequately implemented as indicated in the *table below/attached table*.

Item	Section	CONSORT item	Short explanation
1	Outcomes (6a)	Completely defined pre-specified primary outcome measure, including how and when they were assessed	Is it clear (1) what the primary outcome is (usually the one used in the sample size calculation), (2) how it was measured (if relevant; e.g. which score used), (3) at what time point, and (4) what the analysis metric was (e.g. change from baseline, final value)?
2	Sample size (7a)	How sample size was determined	Is there a clear description of how the sample size was determined, including (1) the estimated outcomes in each group; (2) the α (type I) error level; (3) the statistical power (or the β (type II) error level); and (4) for continuous outcomes, the standard deviation of the measurements?
3	Sequence generation (8a)	Method used to generate random allocation sequence	Does the description make it clear if the "assigned intervention is determined by a chance process and cannot be predicted"?
4	Allocation concealment (9)	Mechanism used to implement random allocation sequence (such as sequentially numbered containers), describing any steps taken to conceal the sequence until interventions were assigned	Is it clear how the care provider enrolling participants was made ignorant of the next assignment in the sequence (different from blinding)? Possible methods can rely on centralised or "third-party" assignment (i.e., use of a central telephone randomisation system, automated assignment system, sealed containers).
5	Blinding (11a)	If done, who was blinded after assignment to interventions (for example, participants, care providers, those assessing outcomes)	Is it clear if (1) healthcare providers, (2) patients, and (3) outcome assessors are blinded to the intervention? General terms such as "double-blind" without further specifications should be avoided.
6	Outcomes and estimation (17a/b)	For the primary outcome, results for each group, and the estimated effect size and its precision (such as 95% confidence intervals)	Is the estimated effect size and its precision (such as standard deviation or 95% confidence intervals) for each treatment arm reported? When the primary outcome is binary, both the relative effect (risk ratio, relative risk) or odds ratio) and the absolute effect (risk difference) should be reported with confidence intervals.
7	Harms (19)	All-important harms or unintended effects in each group	Is the number of affected persons in each group, the severity grade (if relevant) and the absolute risk (e.g. frequency of incidence) reported? Are the number of serious, life threatening events and deaths reported? If no adverse event occurred this should be clearly stated.
8	Registration (23)	Registration number and name of trial registry	Is the registry and the registration number reported? If the trial was not registered, it should be explained why.
9	Protocol (24)	Where trial protocol can be accessed	Is it stated where the trial protocol can be assessed (e.g. published, supplementary file, repository, directly from author, confidential and therefore not available)?
10	Funding (25)	Sources of funding and other support (such as supply of drugs) and role of funders	Are (1) the funding sources, and (2) the role of funder(s) described?

Your efforts are highly appreciated.

Kind regards,

journal name-Team

For peer review only - http://bmjopen.bmj.com/site/about/guidelines.xhtml

Appendix 2: Original study protocol as it was approved by the Medical Sciences Interdivisional Research Ethics Committee of the University of Oxford (R62779/RE001).

CENTRE for STATISTICS in MEDICINE

UNIVERSITY OF OXFORD

Impact of a short form of the CONSORT checklist for peer reviewers to improve the reporting of randomised controlled trials published in biomedical journals: a randomised controlled trial

Short title: CONSORT for Peer Review (CONSORT-PR)

Principal investigator: Dr Benjamin Speich
Centre for Statistics in Medicine, University of Oxford

Dr Benjamin Speich
Centre for Statistics in Medicine,
Botnar Research Centre, University of Oxford, Windmill Road, Oxford OX3 7LD
Email: Benjamin.speich@ndorms.ox.ac.uk

For peer review only

SCIENTIFIC COMMITTEE

Prof Isabelle Boutron, Paris Descartes University, France
Prof Matthias Briel, University of Basel, Switzerland
Associate Prof Sally Hopewell, Centre for Statistics in Medicine, Oxford University, UK
Prof David Moher, Centre for Journalology, Clinical Epidemiology Program, Ottawa Hospital Research Institute, Canada
Prof Philippe Ravaud, Paris Descartes University, France
Dr Benjamin Speich, Centre for Statistics in Medicine, University of Oxford, UK
Dr. Michael M Schlussel, Centre for Statistics in Medicine, University of Oxford, UK

For peer review only - http://bmjopen.bmj.com/site/about/guidelines.xhtml

Dr Sara Schroter, The BMJ, London, UK

Trial registration: This trial will be prospectively registered under clinicaltrials.gov.

Protocol version: Version 1.1 2019-05-21

Funding: Benjamin Speich is supported by an Advanced Postdoc.Mobility grant from the Swiss National Science Foundation (P300PB_177933). David Moher is supported by a University Research Chair, Ottawa. The funders had no role in designing the study and will also have no role in conducting the study as well as in analysing and reporting study results.

Roles and responsibilities:

Contributors: SH, BS, IB, MB, DM, PR, had the study idea and designed the study. SS provided expertise to ensure implementation at the journal level was possible. MMS was responsible for statistical aspects, including the sample size calculation and the data analysis plan. BS and SH wrote the first draft of the study protocol. All authors critically revised the protocol and approved the final version.

Sponsor and contact information: Centre for Statistics in Medicine, Botnar Research Centre, University of Oxford, Windmill Road, Oxford OX3 7LD. Principal investigator: Benjamin Speich (Email: Benjamin.speich@ndorms.ox.ac.uk)

Sponsor and funders: The funders had no role in designing the study and will also have no role in conducting the study as well as in analysing and reporting study results.

Roles and responsibilities: The principal investigator (BS) is responsible for the preparation and the revisions of the study protocol, organising meetings of the steering committee, recruiting and randomizing eligible manuscripts as well as the publication of study reports. The steering committee (IB, MB, SH, DM, PR, BS, MMS, and SS) is in charge of participating in the elaboration of the protocol, defining and validating the additional short explanation for each CONSORT item, following the evolution of the committed study and for publishing the results of this study. MMS is responsible for the sample size calculation and the statistical analyses.

For peer review only - http://bmjopen.bmj.com/site/about/guidelines.xhtml

Table of content:

1. BACKGROUND AND RATIONAL 6
   1.1 Need for clinical research and epidemiologic transparency 6
   1.2 Transparency in published randomised controlled trials 6
   1.3 Journal attempts to improve reporting in published randomised controlled trials 7
2. HYPOTHESIS 8
3. OBJECTIVE 8
   3.1 Main objective 8
4. METHODS 9
   4.1 Trial design 9
   4.2 Study setting and eligibility criteria 9
   4.3 Interventions 10
   4.4 Outcomes 13
   4.5 Participant timeline 14
   4.6. Sample size 16
   4.7 Randomisation and blinding 17
   4.8 Statistical methods 18
      4.8.1 Populations of analysis 18
      4.8.2 Data analysis 19
      4.8.3 Analysis of primary endpoint 19
      4.8.4 Analysis of secondary endpoints 19
5 LEGAL AND GENERAL LOGISTICS 20
   5.1. Organisation of study 20
      5.1.1 Coordinating centre 20
      5.1.2 Scientific committee 20
   5.2. Regulatory aspects 21
6 PUBLICATION POLICY AND ACCESS TO DATA 22
REFERENCES 23
APPENDIX 26

For peer review only

1. Background and rational

1.1 Need for clinical research and epidemiologic transparency

There is substantial agreement that well conducted and reported randomised controlled trials (RCTs) generate the most trustworthy evidence when newly developed or already existing clinical interventions are evaluated (1-3). Besides the complexity and the high associated costs of conducting RCTs (4-6), there are major issues with their reporting that often make it difficult for researchers, clinicians, patients or policymakers to interpret the current evidence on a specific topic (7, 8). Chronologically, the most prominent difficulties in reporting consist of (i) poor reporting in study protocols for RCTs (9-12); (ii) a substantial fraction of trials are not registered, prematurely discontinued (most common due to difficulties with recruitment) and not published (13, 14); and (iii) that published RCTs are often poorly reported (7).

For clinicians, scientists and decision makers, published articles are often the only way to know how a study was conducted. In order to judge the internal and external validity of RCTs, it is crucial that these articles present transparent, accurate and unbiased information about the methods and conduct of the RCT.

1.2 Transparency in published randomised controlled trials

To improve the transparency in clinical and epidemiological research the international organisation called the EQUATOR (Enhancing the Quality and Transparency of Research) Network was founded in 2006 (15-20). This international network consists of researchers, epidemiologists, people in charge of recommendations for the presentation of articles or “reporting guidelines”, statisticians, clinicians and editors from some of the most prestigious journals (e.g., *Lancet, JAMA, Annals of Internal Medicine, BMJ*).

The CONSORT Statement (CONsolidated Standards for Reporting Trials), is perhaps the most important reporting guideline designed to help improve the transparency and quality of reporting of RCTs (21, 22). The CONSORT Statement, consisting of 25 items and a flow diagram which should be reported in papers describing RCTs. The last update of the CONSORT Statement was published simultaneously in 10 leading medical journals in 2010 (23). Currently CONSORT is endorsed by 585 journals (24). The CONSORT Statement guides authors, peer reviewers and journal editors on what information should be included in published reports of RCTs in order to facilitate critical judgment and interpretation of results. It is important to note, that adhering to the CONSORT Statement does not mean that the study is of high quality. However, reporting all items from the CONSORT list will enable readers to adequately judge the quality of RCTs.

For peer review only - http://bmjopen.bmj.com/site/about/guidelines.xhtml

A number of research studies have identified serious limitations in the reporting of published RCTs (3, 25-30). Despite some improvement in reporting following the implementation of the CONSORT Statement, there still remain major reporting deficiencies in published RCTs (31). For example, Odutayo and colleagues showed that a large proportion of RCTs published in December 2012 in PubMed did not define the primary outcome (31%), did not state the sample size calculation (45%) and did not explain the method of allocation concealment (50%) (32). This lack of transparency is a major limiting factor for the reader who assesses an article in order to find the answer to a specific question; it is also a major problem for scientists who perform systematic reviews and meta-analyses. Thus, some published trials may not be included in the meta-analysis because of their lack of transparency. Chan showed (25, 33) that 50% of efficacy outcomes and 65% of safety outcomes could not be included in meta-analyses because of how they were reported. Furthermore, even if these trials are included in systematic reviews and meta-analyses, an adequate risk of bias assessment is often not possible due to the poor reporting quality. Nevertheless, the main consequence of the lack of transparency is the risk of accepting treatments that are ineffective or cause serious adverse events (34).

1.3 Journal attempts to improve reporting in published randomised controlled trials

Journals can play a vital role in improving the reporting of published reports of RCTs. For example, a survey of authors' instructions on journal websites revealed that in 2014 63% (106 of 168) of biomedical journals mentioned CONSORT within their "Instructions to Authors" (35). Of those journals 38 (36%) required a CONSORT checklist as a condition of RCT report submission. Such implementation indicates some improvement over time compared to an assessment in 2007 when only 17 journals requested the CONSORT checklist (36). An interrupted time series analysis which assessed if the CONSORT for Abstracts guideline had an effect on the reporting quality, found that results are better reported in Journals which enforce the policy (37).

In a study published in 2016 authors of RCTs were asked by journal editors to use the web-based CONSORT tool at the manuscript revision stage (38). Authors who were randomly allocated to the intervention had access to a tool which allowed them to combine different CONSORT extensions (according to study design, medical field) to generate customised checklists. In the control group, authors had access to a CONSORT flow diagram generator. The goal was to improve the reporting of CONSORT items with a simple webtool. However, a quarter of all authors either wrongly selected a CONSORT extension or failed to select an extension, indicating that further education is needed in terms of when and how to implement CONSORT extensions.

For peer review only - http://bmjopen.bmj.com/site/about/guidelines.xhtml

A systematic scoping review conducted in 2017 by Blanco and colleagues summarised different interventions aimed to improve adherence to reporting guidelines (39) (manuscript with results currently under review. Draft received via personal communication). A number of different interventions were identified and some had also been tested at journals. However, the interventions, besides requesting submission of checklists from authors, required additional resources at the journal level (e.g. internal peer review by editorial assistants or inviting an additional statistical peer-reviewer (40, 41)). Therefore, it is unlikely that these interventions will be implemented in the vast majority of journals, especially not in smaller journals with limited resources. A study examining "the nature and extent of changes made to manuscripts after peer review, in relation to the reporting of methodological aspects of RCTs" and "the type of changes requested by peer reviewers" found that peer review did lead to some improvement in reporting (40).

Building on these findings we plan to evaluate the impact of inviting peer reviewers to explicitly use a short version of the CONSORT checklist (including a short explanation of those items) as part of their review process. If this intervention deems to be effective, it could be easily implemented by all medical journals without needing additional resources at a journal level.

2. Hypothesis

We propose an RCT to evaluate the impact of asking peer reviewers to use a short version of the CONSORT checklist when reviewing a manuscript of an RCT and whether it improves the completeness of reporting. Our hypothesis is that reminding peer reviewers of the CONSORT items (including a short explanation of those items) will result in higher adherence to CONSORT guidelines in published RCTs. We only selected a limited number of the CONSORT items because we did not want to deter peer reviewers with too much information. Since peer reviewing in general can be burdensome, we felt that this approach is more promising than listing all items, risking that the information will be ignored. The short version of the CONSORT checklist is based on the same items described in a previous study as the 10 most important and underreported CONSORT items (38).

3. Objective

3.1 Main objective

The main objective of this study is to evaluate the impact of asking peer reviewers during the standard peer-review process to ask them to use a short version of the CONSORT checklist (C-short) and whether it will improve the reporting in published RCTs compared to manuscripts where the peer reviewers underwent usual practice.

For peer review only - http://bmjopen.bmj.com/site/about/guidelines.xhtml

4. Methods

4.1 Trial design

This study is a multicentre RCT with articles being the unit of randomisation (Figure 1; allocation ratio 1:1). A multicentre parallel arm RCT with randomisation at the individual article level was chosen instead of a cluster RCT because the risk of any “contamination” on journal level is not given as the intervention will be implemented by an external researcher (i.e. BS). The possibility of contamination due to the possibility that peer reviewer are invited to assess several RCTs and are randomised into both arms was judged as relatively small and therefore we do not plan to adjust for clustering by journal. The journal staff (i.e. editors) will not be actively told which manuscript was allocated to the proposed intervention and which to the control group.

Figure 1: Study flowchart

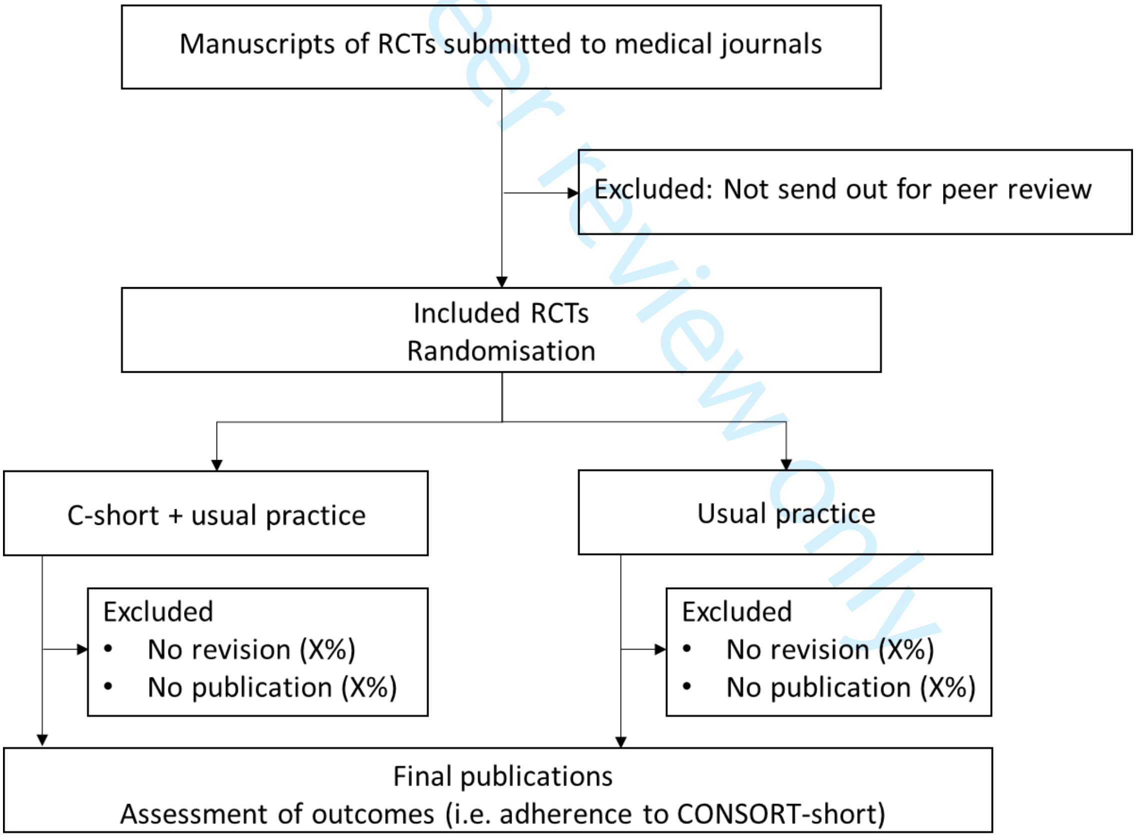

4.2 Study setting and eligibility criteria

The population will be defined on two levels. Included journals and included articles.

Included journals must: i) endorse the CONSORT Statement (e.g. assessed via journals Instruction to Authors); ii) publish primary results of at least five RCTs in 2017 (identified in a brief PubMed search as publishing RCTs in 2017). To be efficient, we plan to contact (via

For peer review only - http://bmjopen.bmj.com/site/about/guidelines.xhtml

email) the editors of eligible journals within a publishing house (i.e. journals which are part of the BMJ series, BMC series, PLoS, Lancet, JAMA) instead of separate journals. A description of the requirements for participation and a short summary information sheet will be included as part of the email invitation sent to journal editors. If a journal is eligible, and agrees to take part, the journal will also need to provide access to their journal editorial system (e.g. ScholarOne, Editorial Manager) to enable the external researcher (i.e. BS) to screen and randomise eligible manuscripts. In cases this is not possible, we will explore with separate journals if it would be possible to grant limited access (e.g. only rights to screen studies) and that the emails from the intervention would be sent by a person from the editorial team.

We will include all submitted manuscripts reporting RCTs for which the journal decides to send out for external peer review. Since the 10 chosen CONSORT checklist items are applicable to different study designs, we will include all RCTs regardless of study design (e.g. parallel group trial, cluster trial, superiority trial, non-inferiority trial). Articles presenting clearly secondary trial results, additional time points, economic analyses, or any other analyses derived from an RCT dataset not including the study's main results will be excluded. Furthermore, RCTs which are clearly labelled as a pilot or feasibility study or randomise animals or cells instead of individuals will be excluded.

Details of journal manuscript submission and peer review processes, including, consent and potential confidentiality issues will be discussed in detail with each journal by teleconference and/or face to face prior to the journal agreeing to take part to ensure that randomisation of manuscripts is feasible. We considered conducting randomisation at the level of the journal (i.e. cluster RCTs). However, in order to make the intervention as easy and simple to implement (and with little or no additional effort from the journal) we believe that randomisation at the manuscript level - with an external researcher implementing the intervention within the existing journal management systems - will be the most efficient study design.

In participating journals, the external investigator (BS) will have access to the editorial management software (e.g. ScholarOne or Editorial Manager) and will check at least twice a week (using automated report lists) all research manuscripts that are sent out for external peer review. As soon as the first peer-reviewer accepts the invitation to review, the manuscript will be randomised to the intervention or control arm (see “Randomisation” for more details). It is possible that this process might be slightly different amongst different included journals.

4.3 Interventions

<u>Experimental group: C-short plus usual practice</u>

For peer review only - http://bmjopen.bmj.com/site/about/guidelines.xhtml

After accepting to review an article, peer reviewers will receive the automated, journal specific standard email with general information as per each journal's usual practice (e.g. where to access the manuscript, date when the peer review report is due). In addition, peer-reviewers who received a manuscript which was randomised to C-short will receive an additional email including a short version of the CONSORT checklist (C-short) (either within the email or a as an attachment; based on the preferences and possibilities of the journal) focusing on the 10 most important and most poorly reported items (Table 1; as previously defined by a group of experts of the CONSORT Group (38)). Peer-reviewers will be asked to pay particular attention to items in the C-short checklist and request authors to report on these items, if not already adequately reported. This second email, containing the C-short checklist, is not generated automatically within the existing journal editorial management system (e.g. ScholarOne or Editorial Manager); it will be sent by the investigator who has access to the journal editorial system (BS). An example of this additional email is presented within the appendix (appendix 1; the exact wording might be changed according to the preferences of the participating journals). At least twice a week the editorial management system will be checked for each journal and if a peer reviewer has accepted an invitation to review, an email containing the C-short intervention will be generated and sent. It might be possible that some journals will only provide the right to access and read manuscripts in the editorial management system, but not to send emails. If this is the case, the corresponding Editor (or designated person within the journal) will be informed to send the emails.

Development and testing of the short explanation of the C-short items:

We chose the 10 most important and poorly reported CONSORT items as identified by a group of CONSORT experts in a previous study conducted by Hopewell and colleagues (38). The selection of the items was based on expert opinion and empirical evidence whenever available (38). In addition, we have added a short explanation for each of the 10 items. These short explanations were extracted and amended from the CONSORT explanation and elaboration paper (21) and from COBWEB which is online writing aid tool (42). The short explanation was discussed and adapted by the scientific committee.

Control group: Usual practice:

After accepting to review an article, peer reviewers will receive the automated, journal specific standard email with general information as per each journal's usual practice (e.g. where to access the manuscript, date until when the peer review report is due). However, they will not receive the second email, sent by the investigator who has access to the journal editorial system (BS) which contains the C-short checklist.

For peer review only - http://bmjopen.bmj.com/site/about/guidelines.xhtml

Table 1: The ten most important and poorly reported CONSORT items as defined by a group of experts on the CONSORT statement (38). For better understanding key features were summarised within a short explanation (extracted from the CONSORT explanation and elaboration paper (21) as well as from the COBWEB tool (42)).

Item	Section	CONSORT item	Short explanation
1	Outcomes (6a)	Completely defined pre-specified primary outcome measure, including how and when they were assessed	Is it clear (1) what the primary outcome is (usually the one used in the sample size calculation), (2) how it was measured (if relevant; e.g. which score used), (3) at what time point, and (4) what the analysis metric was (e.g. change from baseline, final value)?
2	Sample size (7a)	How sample size was determined	Is there a clear description of how the sample size was determined, including (1) the estimated outcomes in each group; (2) the α (type I) error level; (3) the statistical power (or the β (type II) error level); and (4) for continuous outcomes, the standard deviation of the measurements?
3	Sequence generation (8a)	Method used to generate random allocation sequence	Does the description make it clear if the "assigned intervention is determined by a chance process and cannot be predicted"?
4	Allocation concealment (9)	Mechanism used to implement random allocation sequence (such as sequentially numbered containers), describing any steps taken to conceal the sequence until interventions were assigned	Is it clear how the care provider enrolling participants was made ignorant of the next assignment in the sequence (different from blinding)? Possible methods can rely on centralised or "third-party" assignment (i.e., use of a central telephone randomisation system, automated assignment system, sealed containers).
5	Blinding (11a)	If done, who was blinded after assignment to interventions (for example, participants, care providers, those assessing outcomes)	Is it clear if (1) healthcare providers, (2) patients, and (3) outcome assessors are blinded to the intervention? General terms such as "double-blind" without further specifications should be avoided.
6	Outcomes and estimation (17a/b)	For the primary outcome, results for each group, and the estimated effect size and its precision (such as 95% confidence intervals)	Is the estimated effect size and its precision (such as standard deviation or 95% confidence intervals) for each treatment arm reported? When the primary outcome is binary, both the relative effect (risk ratio, relative risk) or odds ratio) and the absolute effect (risk difference) should be reported with confidence intervals.
7	Harms (19)	All-important harms or unintended effects in each group	Is the number of affected persons in each group, the severity grade (if relevant) and the absolute risk (e.g. frequency of incidence) reported? Are the number of serious, life threatening events and deaths reported? If no adverse event occurred this should be clearly stated.
8	Registration (23)	Registration number and name of trial registry	Is the registry and the registration number reported? If the trial was not registered, it should be explained why.
9	Protocol (24)	Where trial protocol can be accessed	Is it stated where the trial protocol can be assessed (e.g. published, supplementary file, repository, directly from author, confidential and therefore not available)?
10	Funding (25)	Sources of funding and other support (such as supply of drugs) and role of funders	Are (1) the funding sources, and (2) the role of the funder(s) described?

For peer review only - http://bmjopen.bmj.com/site/about/guidelines.xhtml

4.4 Outcomes

Primary outcome:

The primary outcome of this study will be the difference of the mean proportion of adequately reported items of the 10 most important and poorly reported CONSORT items between the two intervention arms.

Secondary outcomes:

Secondary outcomes will include the following:

- Mean proportion of adequate reporting of the 10 most important and poorly reported CONSORT items, considering each sub-item (see also "Assessment of outcomes") as a separate item.
- Mean proportion for each of the 10 most important and poorly reported CONSORT items separately (including also separate analysis of sub-items).
- Time from assigning an academic editor until the first decision (as communicated to the author after the first round of peer-review).
- Proportion of articles directly rejected after the first round of peer-review.
- Proportion of articles published.

Additional outcomes:

For journals where peer reviewer comments are subsequently published alongside the published article, we will examine the peer reviewer comments for any reference to CONSORT and trial reporting. We will contact those journals which do not make peer reviewer comments publicly available, to see if they still could be used for such an analyses under the condition that only anonymised data will be published.

Data collection methods:

The outcomes will be assessed independently by two (blinded or at least partially blinded; see "blinding") outcome assessors with expertise in the design and reporting of clinical trials. Any disagreement will be resolved by consensus or if necessary by consulting a third assessor. To ensure consistency between reviewers, we will first pilot the data extraction form; any disparities in the interpretation will be discussed and the data extraction form will be modified accordingly.

Adequate reporting of items will be assessed from published full-text publications adhering to the CONSORT C-short checklist (21). The following included items have sub-items which will be extracted separately:

For peer review only - http://bmjopen.bmj.com/site/about/guidelines.xhtml

- Outcomes (item 6a): (i) Define primary outcome, (ii) how it was measured, (iii) at what time point, and (iv) the analysis metric (e.g. change from baseline, final value).
- Sample size (item 7a): (i) The estimated outcomes in each group, (ii) the α (type I) error level, (iii) the statistical power (or the β (type II) error level), (iv) for continuous outcomes, the standard deviation of the measurements
- Blinding (item 11a): Is the blinding status clear for the following persons: (i) Healthcare provider, (ii) patients, and (iii) outcome assessors.
- Funding (item 25): (i) The funding source, and (ii) the role of funder in the design, conduct, analysis, and reporting.

All items will be judged as either "yes" meaning adequately reported, "no" meaning not adequately reported, or "NA" meaning that this sub-item is not applicable for this RCT. Items with different sub-items will only be judged as adequately reported if all relevant sub-items were adequately reported.

- Time from assigning an academic editor until the first decision: The day when the academic editor was assigned and the day of the first decision (e.g. major revision, minor revision, rejected) will be extracted to calculate the number of days until the first decision.
- Proportion of articles directly rejected after the first round of peer-review: Articles which were not invited for re-submission will be labelled and counted.
- Proportion of articles published: Articles which will be published will be counted and collected for data extraction.

The outcomes "time from assigning an academic editor until the first decision", "proportion of articles directly rejected after the first round of peer-review", and "proportion of articles published" will be extracted directly from editorial management software of the journal.

4.5 Participant timeline

The overview of the study schedule, including enrolment, intervention and assessments is presented in Table 2.

For peer review only - http://bmjopen.bmj.com/site/about/guidelines.xhtml

Table 2: Study schedule

	Enrolment	Allocation and intervention	Intervention	Post-intervention	
Time-point	*Studies which are sent out for peer-review*	*After first peer-reviewer accepts invitation*	*Whenever an additional peer-reviewer accepts invitation*	*First decision by journal*	*Published manuscripts*
Eligibility screen	X				
Allocation		X			
Intervention:					
C-short + usual care		X	X		
Usual care		X	X		
Assessment of trial characteristics:					
Funding source					X
Study centres (single centre or multicentre)					X
Sample size					X
Study design (e.g. parallel arm, crossover)					X
Hypothesis (e.g. superiority, non-inferiority)					X
Medical field					X
Intervention tested					X
Number of trial arms					X
Number of peer-reviewers					X
Journal which published the manuscript					X
Number of journals requesting CONSORT adherence (submission of checklist mandatory)					X
Assessment of outcomes:					
Time from assigning an academic editor until the first decision				X	
Proportion of articles directly rejected after the first round of peer-review				X	
Proportion of articles published					X
Adherence to CONSORT items and sub-items					X

For peer review only

4.6. Sample size

For the sample size calculation we hypothesise in a first scenario (Table 3) that the intervention C-Short will result in a 25% relative increase in adequate reporting compared to the control (meaning that 70% of items will be adequately reported in the intervention group and 56% in the control group). This is based on the rate of reporting of the 10 most important and poorly reported items was 0.56 (meaning that a mean of 56% of the 10 most important and poorly reported items were reported) in the control group of a previous study called WebCONSORT (38). The standard deviation (SD) in the same study was 0.23. However, we calculated our sample size to account for a slightly bigger variability in our data (SD = 0.25).To demonstrate a significant difference with a power of 90% and a type 1 error at 5% a total of 136 articles will be required in this scenario (68 per treatment arm; based on a two sided t-test).

The staff from one journal which will most likely be included (i.e. *PLoS One*) pointed out that 3 out of the 10 assessed items (i.e. item “Registration”, “Protocol”, and “Funding”) should always be implemented given their template. Assuming that this journal will recruit a high proportion, and that also other journals might update their templates, we increased the sample size in a second scenario, in which all these 3 items would have an overall adherence of 90% in the control arm (Table 3). This would entail an overall baseline adherence with the 10 CONSORT-short items of 71%. Based on a two sided t-test, a sample size of 166 (83 per treatment arm) will have a power of 80% to find a 15% relative increase (71% adherence in control group; 82% adherence in intervention group; SD = 0.25; a type 1 error at 5%).

Since the final sample size will be based on the number of articles published, rather than on the number of manuscripts randomised, eligible RCTs will be included and randomised until the number of 83 published RCTs is reached in each arm (resulting in no less than 166 articles), to avoid loss of power due to potential imbalance between arms. Recruitment will be stopped as soon as both arms reach the sample size of 83. After recruitment stop we will wait three month so that manuscripts which are still in production can be published. Manuscripts which are published after the three month period will be excluded.

For peer review only - http://bmjopen.bmj.com/site/about/guidelines.xhtml

Table 3: Assumptions for sample size calculations in two different scenarios.

Item	CONSORT item	Scenario 1. Adequate reporting as published in WebCONSORT	Scenario 2. Adapted from Scenario 1
1	Outcomes (6a)	77% (79 of 103)	77% (79 of 103)
2	Sample size (7a)	83% (85 of 103)	83% (85 of 103)
3	Sequence generation (8a)	76% (78 of 103)	76% (78 of 103)
4	Allocation concealment (9)	55% (57 of 103)	55% (57 of 103)
5	Blinding (11a)	35% (36 of 103)	35% (36 of 103)
6	Outcomes and estimation (17a	44% (45 of 103)	44% (45 of 103)
7	Harms (19)	71% (73 of 103)	71% (73 of 103)
8	Registration (23)	69% (71 of 103)	90%
9	Protocol (24)	19% (20 of 103)	90%
10	Funding (25)	34% (35 of 103)	90%
**Overall**		**56%**	**71%**

Abbreviation: CONSORT= CONsolidated Standards for Reporting Trials

4.7 Randomisation and blinding

Articles, which meet the eligibility criteria as a primary report of an RCT, for which the journal decides to send out for external peer review will be randomised into one of the two groups (allocation 1:1). The randomisation list will be created by the study-randomizer system (43) using random block sizes between 2 and 8 and stratification by journal. As soon as the first peer-reviewer accepts the invitation, the manuscript will be included and randomised to one of the two intervention arms. One of the investigators (BS) will log onto the study randomizer-system (43) entering the study identification number (ID; provided from the Journal), the study title, as well as the journal the study was submitted to. Subsequently, all additional peer-reviewers accepting the invitation to review the same manuscript will receive the same intervention (C-short plus usual practice or usual practice only) as the first peer-reviewer.

Authors will be blinded to the intervention allocation. Editors will not be actively informed about the randomisation (possible exception listed under “4.3 Interventions”). To avoid potential bias, peer reviewers and manuscript authors will not be informed of the study hypothesis, design and intervention.

Outcomes will be assessed in duplicate (see assessment of outcomes). At least one outcome assessors will be blinded. Due to restricted resources it might be possible that the investigator conducting the randomisation (BS) will be included in the data-extraction from published manuscripts.

4.7 Data management and confidentiality

For peer review only - http://bmjopen.bmj.com/site/about/guidelines.xhtml

Outcomes from publications will be assessed and extracted in duplicate. Since this information is not confidential, we will use Google Forms for data extraction from published RCTs. Data entered will be validated for completeness.

Data from the editorial manager software (e.g. Title of manuscript, first author, randomisation ID, Journal, date when manuscript was accepted by and academic editor, date when the final decision was made, final decision, number of peer-reviewers who peer reviewed the manuscript, the peer review) will be extracted, anonymised and entered in a password protected database which is saved on a server from the University of Oxford. Data will be managed and curated according to University of Oxford regulations, which includes regular back-up (on a daily basis) of the virtual drives where the data are stored.

The raw data extracted from the included manuscripts can be made openly accessible in an anonymised way (i.e. giving the included RCT a number instead of identifying them). Derived/aggregated data, including anonymised information generated from the journals' editorial manager software, will be stored and made available to the research community when the project ends (see also "8. Publication policy and access to data"). Where appropriate, the researcher who has access to the editorial manager software (BS) and anyone else who will see the identifiable data will sign a confidentially agreement with the participating journals, confirming that they will not share identifiable data with any other party. Journals such as the BMJ series state in their Company Privacy Statement that research programmes for quality improvement might be in place. Furthermore, peer reviewers for all BMJ journals receive the following statement in their invitation letter "*We are constantly trying to find ways of improving the peer review system and have an ongoing programme of research. If you do not wish your review entered into a study please let us know by emailing […] as soon as possible*."

For peer review only

4.8 Statistical methods

<u>4.8.1 Populations of analysis</u>

The main population for analysis will be all manuscripts randomised and accepted for publication in the participating journals. Differently from RCTs conducted with patients, where drop outs need to be carefully considered (e.g. multiple imputation of missing data), we are only interested in the reporting adherence of RCTs that are published. All outcomes will be calculated based on the main population for analysis. The secondary outcome "Time to the first decision", will additionally be calculated considering all randomised manuscripts (including the ones which were not published).

For peer review only - http://bmjopen.bmj.com/site/about/guidelines.xhtml

4.8.2 Data analysis

All quantitative variables will be described using means and standard deviations, or median and interquartile ranges in case severe departures from a normal distribution are identified. Data distribution will be inspected visually (i.e. by histograms) instead of performing formal statistical tests for normality. Categorical variables will be described using frequencies and percentages. For the primary and secondary outcomes, we will estimate the difference between means between the two groups and report them with respective 95% confidence intervals.

4.8.3 Analysis of primary endpoint

The primary outcome will be the difference of the mean proportion of adequately reported items of the 10 most important and poorly reported CONSORT items. If the data on the primary outcome is normally distributed then the two groups (i.e. C-short plus usual practice vs. usual practice) will be compared using an unpaired Student's t-test to compare the unadjusted mean proportion of adequate reporting. If the data is not normally distributed, comparisons will be performed using a non-parametric equivalent test (i.e. Wilcoxon-Mann-Whitney test for testing whether the population medians of the two groups are the same).

For the analyses of the primary outcomes a p-value of 0.05 (5% significance level) will be used to indicate statistical significance and treatment effect (mean difference) reported with 95% confidence intervals (or median and respective interquartile ranges, in case of asymmetric distribution). Exact p-values will be presented up to three decimal places. We anticipate there will be no missing data in this study, neither at the individual C-short items, nor at the manuscript level. This is due to the study design, which will include only the randomised manuscripts that are accepted for publication.

4.8.4 Analysis of secondary endpoints

To investigate the effect of the intervention on the secondary outcomes, mean differences with respective 95% confidence intervals will also be reported for these outcomes. If normality is not observed for any of the continuous secondary outcomes, the same strategy adopted for the primary outcome (use of a non-parametric equivalent to the Student's *t*-test) will be used.

A p-value of 0.05 will indicate statistical significance for the observed treatment effect on the secondary outcomes. Exact p-values will be presented up to three decimal places. Similarly to the primary outcome, we anticipate there will be no missing data for any of the secondary

For peer review only - http://bmjopen.bmj.com/site/about/guidelines.xhtml

outcomes, as we will have access to the Editorial Management system of the included journals, where all relevant information is automatically reported.

4.8.5 Pre-specified subgroup analysis

No formal subgroup comparative analysis is planned for the primary or secondary outcomes. However, the effect of the intervention on the primary outcome within subgroups, will be presented using forest plots to visually examine whether it differs according to some variables, such as: (1) Journals that actively implement the CONSORT Statement (defined as requiring authors to submit a completed CONSORT checklist alongside their manuscript) vs. journals that are not actively implementing the CONSORT Statement; (2) sample size ($n < 100$ vs. $n \geq 100$); and (3) impact factor (<5, 5.1-10; >10) as there is evidence that higher impact factor as well as higher sample size are associated with higher adherence to reporting guidelines (44). These analyses will be exploratory, with the aim of supporting new hypothesis generation, rather than conclusive.

5 Legal and general logistics

5.1. Organisation of study

5.1.1 Coordinating centre

The coordinating centre's, will be the Centre for Statistics in Medicine at the University of Oxford under the responsibilities of Dr Sally Hopewell and Dr Benjamin Speich.

The coordinating centre's will ensure the following missions:

- Training of the staff
- Implementation of quality control
- Logical controls of data
- Follow-up on requests for correction/validation
- Statistical analysis
- Archiving of data

5.1.2 Scientific committee

The scientific committee is composed of:

- Prof Isabelle Boutron: Centre D'Épidémiologie Clinique Hôtel-Dieu, Paris Descartes University, France
- Prof Matthias Briel, University of Basel, Switzerland
- Associate Prof Sally Hopewell: Centre for Statistics in Medicine, University of Oxford, UK

For peer review only - http://bmjopen.bmj.com/site/about/guidelines.xhtml

- Prof David Moher: Centre for Journalology, Clinical Epidemiology Program, Ottawa Hospital Research Institute, Canada
- Prof Philippe Ravaud: Centre d'Épidémiologie Clinique Hôtel-Dieu, Paris Descartes University, France
- Dr Benjamin Speich, Centre for Statistics in Medicine, University of Oxford, UK
- Dr. Michael M Schlussel, Centre for Statistics in Medicine, University of Oxford, UK
- Dr Sara Schroter, The BMJ, London, UK

The scientific committee is in charge of:

- Participating in the elaboration of the protocol
- Defining and validating the additional short explanation for each CONSORT item.
- Following the evolution of the committed study
- Publishing the results of this study

5.2. Regulatory aspects

Ethical approval for this study will be sought from the Central University Research Ethics Committee (CUREC) of the University of Oxford. Any amendments in the conduct of the study, collection of outcomes or analysis will be reported to the CUREC. The tested intervention has the goal to improve the quality of published journals (i.e. the adherence to CONSORT) and could also be implemented as usual practice without testing at the journal level. In agreement with another study, testing a similar intervention (45), we think that it is ethical to conduct this study without obtaining written consent. The main reason for this procedure are the following:

- Informing the authors and peer-reviewers would make it impossible to measure the effect of our intervention. In short, informing peer-reviewers and authors would create an artificial context which would not be comparable any more to the "real world context". Authors and peer-reviewers would most likely be much more aware of CONSORT if they received information about the study. Furthermore, being aware to participate in a study could strongly influence the natural behaviour of peer-reviewers (e.g. putting more effort into reviewing a manuscript than under "real world conditions") but also of authors.
- The intervention does not pose any risk of harms for authors and peer-reviewers.
- The intervention is not a medical intervention but rather tries to improve the research quality and journal processes.
- Several journal series (e.g. BMJ series) have Company Privacy Statements in place which clearly mention that research programmes might be in place for quality improvement.

For peer review only - http://bmjopen.bmj.com/site/about/guidelines.xhtml

- The intervention could be part of the routine at any Journal without previous assessment of its efficacy.
- No data which identifies participating manuscripts will be published.

6 Publication policy and access to data

The results from this study will be published in a peer-reviewed journal irrespective of the study results. Authorship to publications will be granted according to the rules of the International Committee of Medical Journal Editors (ICMJE). We plan to publish the full anonymised dataset as a supplementary file together with the main publication.

For peer review only

For peer review only - http://bmjopen.bmj.com/site/about/guidelines.xhtml

References

1. Duley L, Antman K, Arena J, Avezum A, Blumenthal M, Bosch J, et al. Specific barriers to the conduct of randomized trials. Clin Trials. 2008;5(1):40-8.
2. Collins R, MacMahon S. Reliable assessment of the effects of treatment on mortality and major morbidity, I: clinical trials. Lancet. 2001;357(9253):373-80.
3. Chalmers I. Unbiased, relevant, and reliable assessments in health care: important progress during the past century, but plenty of scope for doing better. BMJ. 1998;317(7167):1167-8.
4. Collier R. Rapidly rising clinical trial costs worry researchers. CMAJ. 2009;180(3):277-8.
5. Eisenstein EL. Commentary on Sertkaya et al. and Larson et al. Clin Trials. 2016;13(2):137-9.
6. Sertkaya A, Wong HH, Jessup A, Beleche T. Key cost drivers of pharmaceutical clinical trials in the United States. Clin Trials. 2016;13(2):117-26.
7. Glasziou P, Altman DG, Bossuyt P, Boutron I, Clarke M, Julious S, et al. Reducing waste from incomplete or unusable reports of biomedical research. Lancet. 2014;383(9913):267-76.
8. Chan AW, Song F, Vickers A, Jefferson T, Dickersin K, Gotzsche PC, et al. Increasing value and reducing waste: addressing inaccessible research. Lancet. 2014;383(9913):257-66.
9. Chan AW, Tetzlaff JM, Altman DG, Dickersin K, Moher D. SPIRIT 2013: new guidance for content of clinical trial protocols. Lancet. 2013;381(9861):91-2.
10. Chan AW, Tetzlaff JM, Altman DG, Laupacis A, Gotzsche PC, Krleza-Jeric K, et al. SPIRIT 2013 statement: defining standard protocol items for clinical trials. Ann Intern Med. 2013;158(3):200-7.
11. Chan AW, Tetzlaff JM, Gotzsche PC, Altman DG, Mann H, Berlin JA, et al. SPIRIT 2013 explanation and elaboration: guidance for protocols of clinical trials. BMJ. 2013;346:e7586.
12. Lund H, Brunnhuber K, Juhl C, Robinson K, Leenaars M, Dorch BF, et al. Towards evidence based research. BMJ. 2016;355:i5440.
13. Kasenda B, von Elm E, You J, Blumle A, Tomonaga Y, Saccilotto R, et al. Prevalence, characteristics, and publication of discontinued randomized trials. JAMA. 2014;311(10):1045-51.
14. Alturki R, Schandelmaier S, Olu KK, von Niederhausern B, Agarwal A, Frei R, et al. Premature trial discontinuation often not accurately reflected in registries: comparison of registry records with publications. J Clin Epidemiol. 2017;81:56-63.
15. Altman DG, Simera I, Hoey J, Moher D, Schulz K. EQUATOR: reporting guidelines for health research. Lancet. 2008;371(9619):1149-50.
16. Simera I. EQUATOR Network collates resources for good research. BMJ. 2008;337:a2471.
17. Simera I, Altman DG. Writing a research article that is "fit for purpose": EQUATOR Network and reporting guidelines. Evid Based Med. 2009;14(5):132-4.
18. Simera I, Altman DG, Moher D, Schulz KF, Hoey J. Guidelines for reporting health research: the EQUATOR network's survey of guideline authors. PLoS Med. 2008;5(6):e139.
19. Simera I, Moher D, Hirst A, Hoey J, Schulz KF, Altman DG. Transparent and accurate reporting increases reliability, utility, and impact of your research: reporting guidelines and the EQUATOR Network. BMC Med. 2010;8:24.
20. Simera I, Moher D, Hoey J, Schulz KF, Altman DG. The EQUATOR Network and reporting guidelines: Helping to achieve high standards in reporting health research studies. Maturitas. 2009;63(1):4-6.
21. Moher D, Hopewell S, Schulz KF, Montori V, Gotzsche PC, Devereaux PJ, et al. CONSORT 2010 explanation and elaboration: updated guidelines for reporting parallel group randomised trials. BMJ. 2010;340:c869.

For peer review only - http://bmjopen.bmj.com/site/about/guidelines.xhtml

22. Schulz KF, Altman DG, Moher D, Group C. CONSORT 2010 statement: updated guidelines for reporting parallel group randomised trials. BMJ. 2010;340:c332.
23. Schulz KF, Altman DG, Moher D, Group C. CONSORT 2010 Statement: updated guidelines for reporting parallel group randomised trials. BMC Med. 2010;8:18.
24. CONSORT Transparent Reporting of Trials. Endorsers. Journals and Organizations. http://www.consort-statement.org/about-consort/endorsers [accessed: 27 February 2019]
25. Chan AW, Hrobjartsson A, Haahr MT, Gotzsche PC, Altman DG. Empirical evidence for selective reporting of outcomes in randomized trials: comparison of protocols to published articles. JAMA. 2004;291(20):2457-65.
26. Chalmers I, Glasziou P. Avoidable waste in the production and reporting of research evidence. Lancet. 2009;374(9683):86-9.
27. Ioannidis JP, Lau J. Completeness of safety reporting in randomized trials: an evaluation of 7 medical areas. JAMA. 2001;285(4):437-43.
28. Glasziou P, Meats E, Heneghan C, Shepperd S. What is missing from descriptions of treatment in trials and reviews? BMJ. 2008;336(7659):1472-4.
29. Boutron I, Dutton S, Ravaud P, Altman DG. Reporting and interpretation of randomized controlled trials with statistically nonsignificant results for primary outcomes. JAMA. 2010;303(20):2058-64.
30. Hopewell S, Dutton S, Yu LM, Chan AW, Altman DG. The quality of reports of randomised trials in 2000 and 2006: comparative study of articles indexed in PubMed. BMJ. 2010;340:c723.
31. Turner L, Shamseer L, Altman DG, Schulz KF, Moher D. Does use of the CONSORT Statement impact the completeness of reporting of randomised controlled trials published in medical journals? A Cochrane review. Syst Rev. 2012;1:60.
32. Odutayo A, Emdin CA, Hsiao AJ, Shakir M, Copsey B, Dutton S, et al. Association between trial registration and positive study findings: cross sectional study (Epidemiological Study of Randomized Trials-ESORT). BMJ. 2017;356:j917.
33. Chan AW, Krleza-Jeric K, Schmid I, Altman DG. Outcome reporting bias in randomized trials funded by the Canadian Institutes of Health Research. CMAJ. 2004;171(7):735-40.
34. Glass KC. Toward a duty to report clinical trials accurately: the clinical alert and beyond. J Law Med Ethics. 1994;22(4):327-38.
35. Shamseer L, Hopewell S, Altman DG, Moher D, Schulz KF. Update on the endorsement of CONSORT by high impact factor journals: a survey of journal "Instructions to Authors" in 2014. Trials. 2016;17(1):301.
36. Hopewell S, Altman DG, Moher D, Schulz KF. Endorsement of the CONSORT Statement by high impact factor medical journals: a survey of journal editors and journal 'Instructions to Authors'. Trials. 2008;9:20.
37. Hopewell S, Ravaud P, Baron G, Boutron I. Effect of editors' implementation of CONSORT guidelines on the reporting of abstracts in high impact medical journals: interrupted time series analysis. BMJ. 2012;344:e4178.
38. Hopewell S, Boutron I, Altman DG, Barbour G, Moher D, Montori V, et al. Impact of a web-based tool (WebCONSORT) to improve the reporting of randomised trials: results of a randomised controlled trial. BMC Med. 2016;14(1):199.
39. Blanco D, Kirkham JJ, Altman DG, Moher D, Boutron I, Cobo E. Interventions to improve adherence to reporting guidelines in health research: a scoping review protocol. BMJ Open. 2017;7(11):e017551.
40. Hopewell S, Collins GS, Boutron I, Yu LM, Cook J, Shanyinde M, et al. Impact of peer review on reports of randomised trials published in open peer review journals: retrospective before and after study. BMJ. 2014;349:g4145.
41. Cobo E, Selva-O'Callagham A, Ribera JM, Cardellach F, Dominguez R, Vilardell M. Statistical reviewers improve reporting in biomedical articles: a randomized trial. PLoS One. 2007;2(3):e332.

For peer review only - http://bmjopen.bmj.com/site/about/guidelines.xhtml

42. Barnes C, Boutron I, Giraudeau B, Porcher R, Altman DG, Ravaud P. Impact of an online writing aid tool for writing a randomized trial report: the COBWEB (Consort-based WEB tool) randomized controlled trial. BMC Med. 2015;13:221.
43. Study Randomizer. https://studyrandomizer.com/ [accessed: 23. January 2019].
44. Jin Y, Sanger N, Shams I, Luo C, Shahid H, Li G, et al. Does the medical literature remain inadequately described despite having reporting guidelines for 21 years? - A systematic review of reviews: an update. Journal of multidisciplinary healthcare. 2018;11:495-510.
45. Hair K, Macleod MR, Sena ES. A randomised controlled trial of an Intervention to Improve Compliance with the ARRIVE guidelines (IICARus). bioRxiv. 2018.

For peer review only

Appendix

Appendix 1: Example of the email which will be sent out in the intervention arm (C-Short). The exact wording might be slightly adapted according to the journal preferences.

Dear **Title, Name**,

We thank you for accepting to peer-review a manuscript for **journal name**. As we are trying to improve the reporting for randomised controlled trials according to the CONSORT guidelines, we would like to ask if you could check whether the following most important and poorly reported items are adequately implemented as indicated in the *table below/attached table*.

Item	Section	CONSORT item	Short explanation
1	Outcomes (6a)	Completely defined pre-specified primary outcome measure, including how and when they were assessed	Is it clear (1) what the primary outcome is (usually the one used in the sample size calculation), (2) how it was measured (if relevant; e.g. which score used), (3) at what time point, and (4) what the analysis metric was (e.g. change from baseline, final value)?
2	Sample size (7a)	How sample size was determined	Is there a clear description of how the sample size was determined, including (1) the estimated outcomes in each group; (2) the α (type I) error level; (3) the statistical power (or the β (type II) error level); and (4) for continuous outcomes, the standard deviation of the measurements?
3	Sequence generation (8a)	Method used to generate random allocation sequence	Does the description make it clear if the "assigned intervention is determined by a chance process and cannot be predicted"?
4	Allocation concealment (9)	Mechanism used to implement random allocation sequence (such as sequentially numbered containers), describing any steps taken to conceal the sequence until interventions were assigned	Is it clear how the care provider enrolling participants was made ignorant of the next assignment in the sequence (different from blinding)? Possible methods can rely on centralised or "third-party" assignment (i.e., use of a central telephone randomisation system, automated assignment system, sealed containers).
5	Blinding (11a)	If done, who was blinded after assignment to interventions (for example, participants, care providers, those assessing outcomes)	Is it clear if (1) healthcare providers, (2) patients, and (3) outcome assessors are blinded to the intervention? General terms such as "double-blind" without further specifications should be avoided.
6	Outcomes and estimation (17a/b)	For the primary outcome, results for each group, and the estimated effect size and its precision (such as 95% confidence intervals)	Is the estimated effect size and its precision (such as standard deviation or 95% confidence intervals) for each treatment arm reported? When the primary outcome is binary, both the relative effect (risk ratio, relative risk) or odds ratio) and the absolute effect (risk difference) should be reported with confidence intervals.
7	Harms (19)	All-important harms or unintended effects in each group	Is the number of affected persons in each group, the severity grade (if relevant) and the absolute risk (e.g. frequency of incidence) reported? Are the number of serious, life threatening events and deaths reported? If no adverse event occurred this should be clearly stated.
8	Registration (23)	Registration number and name of trial registry	Is the registry and the registration number reported? If the trial was not registered, it should be explained why.
9	Protocol (24)	Where trial protocol can be accessed	Is it stated where the trial protocol can be assessed (e.g. published, supplementary file, repository, directly from author, confidential and therefore not available)?
10	Funding (25)	Sources of funding and other support (such as supply of drugs) and role of funders	Are (1) the funding sources, and (2) the role of funder(s) described?

Your efforts are highly appreciated.

Kind regards,

journal name-Team

For peer review only - http://bmjopen.bmj.com/site/about/guidelines.xhtml

Appendix 3: WHO Trial Registration Data Set (Version 1.3.1)

Statement was filled out on the 01. October 2019.

1. Primary Registry and Trial Identifying Number

This trial was denied registration on ClinicalTrials.gov as the study is not a clinical study that assesses a health outcome in human subjects. Instead we registered the trial on the Open Science Framework (https://osf.io/c4hn8).

2. Date of Registration in Primary Registry

21. June 2019

3. Secondary Identifying Numbers

Not applicable

4. Source(s) of Monetary or Material Support

No specific funding was acquired for this study. Benjamin Speich is supported by an Advanced Postdoc.Mobility grant from the Swiss National Science Foundation (P300PB_177933). David Moher is supported by a University Research Chair, Ottawa. Michael M Schlussel is funded by Cancer Research UK. The funders had no role in designing the study and will also have no role in conducting the study, or analysing and reporting study results.

5. Primary Sponsor

Sponsor:	University of Oxford
Principal Investigator/Sponsor Investigator:	Benjamin Speich, PhD<br>Postdoctoral Researcher<br>Centre for Statistics in Medicine (CSM)<br>Nuffield Department of Orthopaedics, Rheumatology and Musculoskeletal Sciences (NDORMS)<br>University of Oxford<br>Windmill Road<br>Oxford OX3 7LD<br>Tel: +44 1865 737904<br><br>Email: benjamin.speich@ndorms.ox.ac.uk

6. Secondary Sponsor(s)

Not applicable

For peer review only - http://bmjopen.bmj.com/site/about/guidelines.xhtml

7. Contact for Public Queries

Dr. Benjamin Speich

Tel: +44 1865 737904

Email: benjamin.speich@ndorms.ox.ac.uk

8. Contact for Scientific Queries

Sponsor:	University of Oxford
Principal Investigator/Sponsor Investigator:	Benjamin Speich, PhD<br>Postdoctoral Researcher<br>Centre for Statistics in Medicine (CSM)<br>Nuffield Department of Orthopaedics, Rheumatology and Musculoskeletal Sciences (NDORMS)<br>University of Oxford<br>Windmill Road<br>Oxford OX3 7LD<br>Tel: +44 1865 737904<br><br>Email: benjamin.speich@ndorms.ox.ac.uk

9. Public Title

Impact of checklists to improve the reporting of randomised controlled trials published in biomedical journals

10. Scientific Title

Impact of a short version of the CONSORT checklist for peer reviewers to improve the reporting of randomised controlled trials published in biomedical journals: a randomised controlled trial

Running title: CONSORT for Peer Review (CONSORT-PR)

Study identifier: CONSORT-PR

11. Countries of Recruitment

Multinational (Centres are Biomedical journals)

12. Health Condition(s) or Problem(s) Studied

Reporting in published randomised controlled trials

13. Intervention(s)

<u>Control group: Usual practice</u>

For peer review only - http://bmjopen.bmj.com/site/about/guidelines.xhtml

For peer review only

After accepting to review a manuscript, peer reviewers will receive the automated, journal specific standard email with general information as per each journal's usual practice (e.g. where to access the manuscript, date the peer review report is due).

Intervention group: C-short plus usual practice

After accepting to review a manuscript, peer reviewers will receive the automated, journal specific standard email with general information (identical to control group). In addition, peer reviewers will receive an additional email from the editorial office that includes a short version of the CONSORT checklist (C-short) together with a brief explanation of the items either as a table within the email or as an attachment. Peer reviewers will be asked to check whether the items in the C-short checklist are addressed in the manuscript and to request authors to include these items if they are not adequately reported.

14. Key Inclusion and Exclusion Criteria

The population will be defined on two levels: included journals and included manuscripts.

Inclusion criteria for journals:

Included journals must: i) endorse the CONSORT Statement by mentioning it in the journals' Instruction to Authors; ii) have published primary results of at least five RCTs in 2017 (identified using a PubMed search).

Inclusion criteria for manuscripts

- All new manuscript submissions reporting the primary results of RCTs, which the journal editor has decided to send out for external peer review. Since the 10 chosen CONSORT checklist items (C-short) are applicable to different study designs, we will include all manuscripts reporting the primary results of RCTs regardless of study design (e.g. parallel group trial, cluster trial, superiority trial, non-inferiority/equivalence trials).

Exclusion criteria for manuscripts

- Manuscripts clearly presenting secondary trial results, additional time points, economic analyses, or any other analyses.
- Manuscripts which are clearly labelled as a pilot or feasibility study or animal studies.
- Manuscripts not sent for peer review.

15. Study Type

This study is a multicentre RCT with submitted manuscripts as the unit of randomisation (allocation ratio 1:1).

For peer review only - http://bmjopen.bmj.com/site/about/guidelines.xhtml

16. Date of First Enrollment

22. July 2019

17. Sample Size

166 Since the final sample size will be based on the number of articles published, rather than on the number of manuscripts randomised, eligible manuscripts will be randomised until 83 articles are published in each arm (resulting in no less than 166 articles), to avoid loss of power due to potential imbalance between arms.

18. Recruitment Status

Recruiting

19. Primary Outcome(s)

- The primary outcome of this study will be the difference in the mean proportion of adequately reported C-short items in published articles between the two groups.

20. Key Secondary Outcomes

- Mean proportion of adequately reported C-short items in published articles considering each item separately.
- Difference in mean proportion of adequately reported C-short items in published articles considering each sub-item (see "Assessment of outcomes") as a separate item.
- Time from assigning an editor to the first decision (as communicated to the author after the first round of peer-review).
- Proportion of manuscripts rejected after the first round of peer review.
- Proportion of manuscripts that will be published in the journal under study.

21. Ethics Review

Ethical approval has been obtained from the Medical Sciences Interdivisional Research Ethics Committee of the University of Oxford (R62779/RE001).

22. Completion date

We expect that recruitment will be finished in summer 2021.

23. Summary Results

Not applicable

24. IPD sharing statement

We plan to make the anonymised dataset, including the data from the published articles, available as a supplementary file of the main publication.

For peer review only - http://bmjopen.bmj.com/site/about/guidelines.xhtml

Impact of a short form of the CONSORT checklist for peer reviewers to improve the reporting of randomised controlled trials published in biomedical journals: study protocol for a randomised controlled trial

Benjamin Speich[1,2,*], Sara Schroter[3], Matthias Briel[2,4], David Moher[5], Michael M Schlussel[1], Philippe Ravaud[6,7], Isabelle Boutron[6,7], Sally Hopewell[1]

SPIRIT 2013 Checklist: Recommended items to address in a clinical trial protocol and related documents*

STANDARD PROTOCOL ITEMS: RECOMMENDATIONS FOR INTERVENTIONAL TRIALS

**Section/item**	**Item No**	**Description**	**Addressed on page number**
**Administrative information**			
Title	1	Descriptive title identifying the study design, population, interventions, and, if applicable, trial acronym	1____________
Trial registration	2a	Trial identifier and registry name. If not yet registered, name of intended registry	3______
	2b	All items from the World Health Organization Trial Registration Data Set	Appendix 3
Protocol version	3	Date and version identifier	Appendix 1
Funding	4	Sources and types of financial, material, and other support	2_____________
Roles and responsibilities	5a	Names, affiliations, and roles of protocol contributors	1 and 19-20__
	5b	Name and contact information for the trial sponsor	1 and Appendix 1_

For peer review only

	5c	Role of study sponsor and funders, if any, in study design; collection, management, analysis, and interpretation of data; writing of the report; and the decision to submit the report for publication, including whether they will have ultimate authority over any of these activities	2_______
	5d	Composition, roles, and responsibilities of the coordinating centre, steering committee, endpoint adjudication committee, data management team, and other individuals or groups overseeing the trial, if applicable (see Item 21a for data monitoring committee)	20__________
**Introduction**			
Background and rationale	6a	Description of research question and justification for undertaking the trial, including summary of relevant studies (published and unpublished) examining benefits and harms for each intervention	5-6__________
	6b	Explanation for choice of comparators	5-6 (comparator, usual practice)______
Objectives	7	Specific objectives or hypotheses	6
Trial design	8	Description of trial design including type of trial (eg, parallel group, crossover, factorial, single group), allocation ratio, and framework (eg, superiority, equivalence, noninferiority, exploratory)	7____________
**Methods: Participants, interventions, and outcomes**			
Study setting	9	Description of study settings (eg, community clinic, academic hospital) and list of countries where data will be collected. Reference to where list of study sites can be obtained	7-9__________
Eligibility criteria	10	Inclusion and exclusion criteria for participants. If applicable, eligibility criteria for study centres and individuals who will perform the interventions (eg, surgeons, psychotherapists)	7-9_
Interventions	11a	Interventions for each group with sufficient detail to allow replication, including how and when they will be administered	9-10, Table 1, Appendix_____

For peer review only - http://bmjopen.bmj.com/site/about/guidelines.xhtml

	11b	Criteria for discontinuing or modifying allocated interventions for a given trial participant (eg, drug dose change in response to harms, participant request, or improving/worsening disease)	NA (one time intervention)
	11c	Strategies to improve adherence to intervention protocols, and any procedures for monitoring adherence (eg, drug tablet return, laboratory tests)	NA (one time intervention)
	11d	Relevant concomitant care and interventions that are permitted or prohibited during the trial	NA_______
Outcomes	12	Primary, secondary, and other outcomes, including the specific measurement variable (eg, systolic blood pressure), analysis metric (eg, change from baseline, final value, time to event), method of aggregation (eg, median, proportion), and time point for each outcome. Explanation of the clinical relevance of chosen efficacy and harm outcomes is strongly recommended	10-12______
Participant timeline	13	Time schedule of enrolment, interventions (including any run-ins and washouts), assessments, and visits for participants. A schematic diagram is highly recommended (see Figure)	Figure 1 and Table Table 2________
Sample size	14	Estimated number of participants needed to achieve study objectives and how it was determined, including clinical and statistical assumptions supporting any sample size calculations	13-14__________
Recruitment	15	Strategies for achieving adequate participant enrolment to reach target sample size	7-8, 13-14___

Methods: Assignment of interventions (for controlled trials)

Allocation:

Sequence generation	16a	Method of generating the allocation sequence (eg, computer-generated random numbers), and list of any factors for stratification. To reduce predictability of a random sequence, details of any planned restriction (eg, blocking) should be provided in a separate document that is unavailable to those who enrol participants or assign interventions	14________
Allocation concealment mechanism	16b	Mechanism of implementing the allocation sequence (eg, central telephone; sequentially numbered, opaque, sealed envelopes), describing any steps to conceal the sequence until interventions are assigned	14_________
Implementation	16c	Who will generate the allocation sequence, who will enrol participants, and who will assign participants to interventions	14_________

Blinding (masking)	17a	Who will be blinded after assignment to interventions (eg, trial participants, care providers, outcome assessors, data analysts), and how	14_____________
	17b	If blinded, circumstances under which unblinding is permissible, and procedure for revealing a participant's allocated intervention during the trial	NA__________
**Methods: Data collection, management, and analysis**			
Data collection methods	18a	Plans for assessment and collection of outcome, baseline, and other trial data, including any related processes to promote data quality (eg, duplicate measurements, training of assessors) and a description of study instruments (eg, questionnaires, laboratory tests) along with their reliability and validity, if known. Reference to where data collection forms can be found, if not in the protocol	11-12__
	18b	Plans to promote participant retention and complete follow-up, including list of any outcome data to be collected for participants who discontinue or deviate from intervention protocols	14 (no missing data expected)__
Data management	19	Plans for data entry, coding, security, and storage, including any related processes to promote data quality (eg, double data entry; range checks for data values). Reference to where details of data management procedures can be found, if not in the protocol	15-16__
Statistical methods	20a	Statistical methods for analysing primary and secondary outcomes. Reference to where other details of the statistical analysis plan can be found, if not in the protocol	13-15________
	20b	Methods for any additional analyses (eg, subgroup and adjusted analyses)	16_____
	20c	Definition of analysis population relating to protocol non-adherence (eg, as randomised analysis), and any statistical methods to handle missing data (eg, multiple imputation)	15 (no missing data expected)___
**Methods: Monitoring**			
Data monitoring	21a	Composition of data monitoring committee (DMC); summary of its role and reporting structure; statement of whether it is independent from the sponsor and competing interests; and reference to where further details about its charter can be found, if not in the protocol. Alternatively, an explanation of why a DMC is not needed	17_________
	21b	Description of any interim analyses and stopping guidelines, including who will have access to these interim results and make the final decision to terminate the trial	13___________

For peer review only - http://bmjopen.bmj.com/site/about/guidelines.xhtml

Harms	22	Plans for collecting, assessing, reporting, and managing solicited and spontaneously reported adverse events and other unintended effects of trial interventions or trial conduct	NA__
Auditing	23	Frequency and procedures for auditing trial conduct, if any, and whether the process will be independent from investigators and the sponsor	17__________
**Ethics and dissemination**			
Research ethics approval	24	Plans for seeking research ethics committee/institutional review board (REC/IRB) approval	20_____________
Protocol amendments	25	Plans for communicating important protocol modifications (eg, changes to eligibility criteria, outcomes, analyses) to relevant parties (eg, investigators, REC/IRBs, trial participants, trial registries, journals, regulators)	20_
Consent or assent	26a	Who will obtain informed consent or assent from potential trial participants or authorised surrogates, and how (see Item 32)	Appendix 2_____
	26b	Additional consent provisions for collection and use of participant data and biological specimens in ancillary studies, if applicable	NA__________
Confidentiality	27	How personal information about potential and enrolled participants will be collected, shared, and maintained in order to protect confidentiality before, during, and after the trial	17_
Declaration of interests	28	Financial and other competing interests for principal investigators for the overall trial and each study site	20_____________
Access to data	29	Statement of who will have access to the final trial dataset, and disclosure of contractual agreements that limit such access for investigators	21__
Ancillary and post-trial care	30	Provisions, if any, for ancillary and post-trial care, and for compensation to those who suffer harm from trial participation	NA__
Dissemination policy	31a	Plans for investigators and sponsor to communicate trial results to participants, healthcare professionals, the public, and other relevant groups (eg, via publication, reporting in results databases, or other data sharing arrangements), including any publication restrictions	21_________
	31b	Authorship eligibility guidelines and any intended use of professional writers	21____________

For peer review only

	31c	Plans, if any, for granting public access to the full protocol, participant-level dataset, and statistical code	Intend to publish in BMJ open (protocol), dataset: page 21
**Appendices**			
Informed consent materials	32	Model consent form and other related documentation given to participants and authorised surrogates	Appendix 2 (no consent)_
Biological specimens	33	Plans for collection, laboratory evaluation, and storage of biological specimens for genetic or molecular analysis in the current trial and for future use in ancillary studies, if applicable	NA_________

*It is strongly recommended that this checklist be read in conjunction with the SPIRIT 2013 Explanation & Elaboration for important clarification on the items. Amendments to the protocol should be tracked and dated. The SPIRIT checklist is copyrighted by the SPIRIT Group under the Creative Commons "Attribution-NonCommercial-NoDerivs 3.0 Unported" license.

For peer review only

BMJ Open

Impact of a short version of the CONSORT checklist for peer reviewers to improve the reporting of randomised controlled trials published in biomedical journals: study protocol for a randomised controlled trial

Journal:	*BMJ Open*
Manuscript ID	bmjopen-2019-035114.R1
Article Type:	Protocol
Date Submitted by the Author:	05-Feb-2020

Complete List of Authors:	Speich, Benjamin; University of Oxford, Centre for Statistics in Medicine, Nuffield Department of Orthopaedics, Rheumatology and Musculoskeletal Sciences<br>Schroter, Sara; BMJ Editorial,<br>Briel, Matthias; University of Basel, Basel Institute for Clinical Epidemiology and Biostatistics, Department of Clinical Research, University Hospital Basel<br>Moher, David; Ottawa Hospital Research Institute, Ottawa Methods Centre<br>Puebla, Iratxe ; PLOS ONE, PLOS ONE, Public Library of Science, San Francisco, California, United States of America and Cambridge<br>Clark, Alejandra ; PLOS One, PLOS ONE, Public Library of Science, San Francisco, California, United States of America and Cambridge<br>Maia Schlüssel, Michael; University of Oxford, Centre for Statistics in Medicine, Nuffield Department of Orthopaedics, Rheumatology and Musculoskeletal Sciences<br>Ravaud, Philippe; Universite Paris Descartes,<br>Boutron, Isabelle; Université Paris Descartes, Centre d\'épidémiologie clinique<br>Hopewell, Sally; University of Oxford, Centre for Statistics in Medicine, Nuffield Department of Orthopaedics, Rheumatology and Musculoskeletal Sciences

**Primary Subject Heading**:	Medical publishing and peer review
Secondary Subject Heading:	Medical education and training, Medical publishing and peer review, Research methods
Keywords:	Protocols & guidelines < HEALTH SERVICES ADMINISTRATION & MANAGEMENT, Quality in health care < HEALTH SERVICES ADMINISTRATION & MANAGEMENT, MEDICAL EDUCATION & TRAINING, STATISTICS & RESEARCH METHODS

I, the Submitting Author has the right to grant and does grant on behalf of all authors of the Work (as defined in the below author licence), an exclusive licence and/or a non-exclusive licence for contributions from authors who are: i) UK Crown employees; ii) where BMJ has agreed a CC-BY licence shall apply, and/or iii) in accordance with the terms applicable for US Federal Government officers or employees acting as part of their official duties; on a worldwide, perpetual, irrevocable, royalty-free basis to BMJ Publishing Group Ltd ("BMJ") its licensees and where the relevant Journal is co-owned by BMJ to the co-owners of the Journal, to publish the Work in this journal and any other BMJ products and to exploit all rights, as set out in our licence.

The Submitting Author accepts and understands that any supply made under these terms is made by BMJ to the Submitting Author unless you are acting as an employee on behalf of your employer or a postgraduate student of an affiliated institution which is paying any applicable article publishing charge ("APC") for Open Access articles. Where the Submitting Author wishes to make the Work available on an Open Access basis (and intends to pay the relevant APC), the terms of reuse of such Open Access shall be governed by a Creative Commons licence – details of these licences and which Creative Commons licence will apply to this Work are set out in our licence referred to above.

Other than as permitted in any relevant BMJ Author's Self Archiving Policies, I confirm this Work has not been accepted for publication elsewhere, is not being considered for publication elsewhere and does not duplicate material already published. I confirm all authors consent to publication of this Work and authorise the granting of this licence.

For peer review only

Impact of a short version of the CONSORT checklist for peer reviewers to improve the reporting of randomised controlled trials published in biomedical journals: study protocol for a randomised controlled trial

Benjamin Speich[1,2,*], Sara Schroter[3], Matthias Briel[2,4], David Moher[5,6], Iratxe Puebla[7], Alejandra Clark[7], Michael M Schlussel[1,8], Philippe Ravaud[9,10], Isabelle Boutron[9,10], Sally Hopewell[1]

[1]Centre for Statistics in Medicine, Nuffield Department of Orthopaedics, Rheumatology and Musculoskeletal Sciences, University of Oxford, Oxford, United Kingdom

[2]Basel Institute for Clinical Epidemiology and Biostatistics, Department of Clinical Research, University Hospital Basel, University of Basel, Basel, Switzerland

[3]The BMJ, London, United Kingdom

[4]Department of Health Research Methods, Evidence, and Impact, McMaster University, Hamilton, Canada

[5]Centre for Journalology, Clinical Epidemiology Program, Ottawa Hospital Research Institute, Canada

[6]School of Epidemiology and Public Health, Faculty of Medicine, University of Ottawa

[7]*PLOS ONE*, Public Library of Science, San Francisco, California, United States of America and Cambridge, United Kingdom

[8]The EQUATOR Network, Oxford, United Kingdom

[9]Centre d'Épidémiologie Clinique, Hôpital Hôtel-Dieu, Assistance Publique Hôpitaux de Paris (APHP) F-75004 Paris, France

[10]Université de Paris, CRESS, Inserm, INRA, F-75004, Paris, France

*Corresponding author:

Benjamin Speich, PhD,
Centre for Statistics in Medicine, Nuffield Department of Orthopaedics, Rheumatology and Musculoskeletal Sciences,

For peer review only

For peer review only - http://bmjopen.bmj.com/site/about/guidelines.xhtml

University of Oxford,
Windmill Road
OX3 7LD Oxford
United Kingdom
Email: benjamin.speich@ndorms.ox.ac.uk

E-mail addresses of all co-authors:

Benjamin Speich <benjamin.speich@ndorms.ox.ac.uk>

Sara Schroter <sschroter@bmj.com>

Matthias Briel <matthias.briel@usb.ch>

David Moher <dmoher@ohri.ca>

Iratxe Puebla <ipuebla@plos.org>

Alejandra Clark <aclark@plos.org>

Michael M Schlussel <michael.schlussel@ndorms.ox.ac.uk>

Philippe Ravaud <philipperavaud@gmail.com>

Isabelle Boutron <isabelle.boutron@aphp.fr>

Sally Hopewell <sally.hopewell@csm.ox.ac.uk>

Running title: CONSORT for Peer Review (CONSORT-PR)

Keywords: CONSORT; reporting guidelines; randomised controlled trials as topic; peer review; meta-research

For peer review only

For peer review only - http://bmjopen.bmj.com/site/about/guidelines.xhtml

Abstract

Introduction: Transparent and accurate reporting is essential for readers to adequately interpret the results of a study. Journals can play a vital role in improving the reporting of published randomised controlled trials (RCTs). We describe an RCT to evaluate our hypothesis that asking peer reviewers to check whether the most important and poorly reported CONSORT (CONsolidated Standards for Reporting Trials) items are adequately reported, will result in higher adherence to CONSORT guidelines in published RCTs.

Methods and Analysis: Manuscripts presenting the primary results of RCTs submitted to participating journals will be randomised to either the intervention group (peer reviewers will receive a reminder and short explanation of the ten most important and poorly reported CONSORT items; they will be asked to check if these items are reported in the submitted manuscript) or a control group (usual journal practice). The primary outcome will be the mean proportion of the ten items that are adequately reported in the published articles. Peer reviewers and manuscript authors will not be informed of the study hypothesis, design, or intervention. Outcomes will be assessed in duplicate from published articles by two data extractors (at least one blinded to the intervention). We will enrol eligible manuscripts until a minimum of 83 articles per group (166 in total) are published.

Ethics and Dissemination: This pragmatic RCT was approved by the Medical Sciences Interdivisional Research Ethics Committee of the University of Oxford (R62779/RE001). If this intervention is effective, it could be implemented by all medical journals without requiring large additional resources at journal level. Findings will be disseminated through presentations in relevant conferences and peer-reviewed publications. This trial is registered on the Open Science Framework (https://osf.io/c4hn8).

For peer review only

For peer review only - http://bmjopen.bmj.com/site/about/guidelines.xhtml

Strengths and limitations of this study

- Pragmatic randomised controlled trial (RCT) with individual randomisation of real manuscripts describing RCTs submitted to a variety of journals.
- Main outcomes will be assessed from publicly available sources (i.e. published articles).
- If this simple intervention is effective, it could be implemented by journals without requiring large additional resources at journal level.
- The intervention could not be included within the email from journal with the link to the manuscript for review, risking peer reviewers will potentially ignore the separate email containing the CONSORT reminder.

For peer review only

For peer review only - http://bmjopen.bmj.com/site/about/guidelines.xhtml

Introduction

Background and rationale

There is substantial agreement that well conducted and reported randomised controlled trials (RCTs) generate the most trustworthy evidence when evaluating newly developed or existing clinical interventions.[1-3] For clinicians, scientists and decision makers, published articles are often the only way to know how a study was conducted. In order to judge the internal and external validity of RCTs, it is crucial that these articles present transparent, accurate and unbiased information about the methods and conduct of the RCT.

To improve the quality and transparency of clinical and epidemiological research, the EQUATOR (Enhancing the Quality and Transparency of Research) Network was founded in 2006 and officially launched in 2008.[4-10] This international network, which assists in the development of reporting guidelines and actively promotes their use, consists of methodologists, epidemiologists, reporting guideline developers, statisticians, clinicians and journal editors.

The CONSORT Statement (CONsolidated Standards of Reporting Trials) is perhaps the most prominent reporting guideline, designed to help improve the transparency and quality of reporting of RCTs.[11-13] It guides authors, peer reviewers and journal editors on the minimum information to be included in published reports of RCTs to facilitate critical judgment and interpretation of results and consists of 25 items and a flow diagram. The last update of the CONSORT Statement was published simultaneously in 10 leading medical journals in 2010[13] and currently CONSORT is endorsed by over 600 journals worldwide.[14]

Despite some improvement in reporting following the endorsement of the CONSORT Statement, there remain major reporting deficiencies in published RCTs.[3 15-21] For example, a study of 1122 RCTs indexed in PubMed in December 2012 found that many did not define the

For peer review only - http://bmjopen.bmj.com/site/about/guidelines.xhtml

primary outcome (31%), state the sample size calculation (45%), or explain the method of allocation concealment (50%).[22] This lack of transparency is a major limiting factor for readers who assess an article in order to find the answer to a specific question; it is also a major problem for scientists who perform systematic reviews and meta-analyses.

Evidence to date

Journals can play a vital role in improving the reporting of published RCTs. For example, a survey of journals' 'Instructions to Authors' in 2014 found that 63% (106 of 168) of biomedical journals mentioned CONSORT;[23] however of those journals only 38 (36%) required a completed CONSORT checklist on submission. Such implementation indicates some improvement over time compared to an assessment in 2007 when only 17 of 62 (27%) journals requested the CONSORT checklist on submission.[24] A study using interrupted time series analysis and assessing if the CONSORT checklist for reporting abstracts of RCTs had an effect on reporting quality found that results were better reported in journals which had an active editorial policy to implement the checklist.[25]

A scoping review conducted in 2017 by Blanco and colleagues summarised different interventions aimed at improving adherence to reporting guidelines.[26] They identified a number of different interventions, some of which had been evaluated at journals. However, all the interventions, except requesting submission of checklists from authors, required additional resources from the journal (e.g. internal peer review by editorial assistants or an additional peer-reviewer round conducted by a senior statistician using appropriate reporting guidelines[27-29]). Therefore, it is unlikely that these interventions will be implemented in the majority of journals, especially smaller journals with limited resources. Another study found that providing authors with a web-based CONSORT tool, which combined different CONSORT extensions and provided authors with a customised checklist, did not improve reporting when used at the manuscript revision stage.[30] However, a study examining "the nature and extent of changes

For peer review only - http://bmjopen.bmj.com/site/about/guidelines.xhtml

made to manuscripts after peer review, in relation to the reporting of methodological aspects of RCTs" and "the type of changes requested by peer reviewers" found that peer review did lead to some improvement in reporting.[27]

The role of peer reviewers and expectations of them is varied.[31] While CONSORT checklists are sometimes available for peer reviewers to check, they are not typically instructed to assess this information as part of their review and there have been no studies evaluating the effect of asking them to do this. We plan to evaluate the impact of giving peer reviewers a short version of the CONSORT checklist together with a brief explanation of the items and asking them to check if they are adequately reported.

Methods and analysis

Objective

The objective of this study is to evaluate the impact of giving peer reviewers, during the standard peer review process, a short version of the CONSORT checklist (C-short) together with a brief explanation of the items and asking them to check if they are adequately reported in the manuscript.

Study design

This study is a multicentre superiority RCT with submitted manuscripts as the unit of randomisation (Figure 1; allocation ratio 1:1). This study protocol was written in adherence to the SPIRIT guidelines (supplementary file).[32]

Study setting and eligibility criteria

The population will be defined on two levels: included journals and included manuscripts.

For peer review only - http://bmjopen.bmj.com/site/about/guidelines.xhtml

Inclusion criteria for journals:

Included journals must: i) endorse the CONSORT Statement by mentioning it in the journals' Instruction to Authors; ii) have published primary results of at least five RCTs in 2017 (identified using a PubMed search). To be efficient, we plan to contact (via email) the editors of eligible journals from specific publishers (e.g. BMJ Publishing Group; Public Library of Science [PLOS]) instead of separate journals. A description of the requirements for participation and a short summary information sheet will be included as part of the email invitation sent to journal editors. If a journal is eligible, and the editor agrees to take part, the editor will need to provide access to their editorial system (e.g. ScholarOne, Editorial Manager) to enable the external researcher (BS) to screen and randomise eligible manuscripts. In cases where this is not possible, we will explore with individual journals if it would be possible to grant limited access (e.g. only rights to screen studies) or to handle the different steps without access to the editorial system (e.g. screening through automated reports; intervention provided by a journal staff member) and that the emails for the intervention would be sent by a member of the editorial team.

Inclusion criteria for manuscripts

- All new manuscript submissions reporting the primary results of RCTs, which the journal editor has decided to send out for external peer review. Since the 10 chosen CONSORT checklist items (C-short) are applicable to different study designs, we will include all manuscripts reporting the primary results of RCTs regardless of study design (e.g. parallel group trial, cluster trial, superiority trial, non-inferiority/equivalence trials).

Exclusion criteria for manuscripts

- Manuscripts clearly presenting secondary trial results, additional time points, economic analyses, or any other analyses.
- Manuscripts which are clearly labelled as a pilot or feasibility study or animal studies.

For peer review only - http://bmjopen.bmj.com/site/about/guidelines.xhtml

- Manuscripts not sent for peer review.

Details of journal manuscript submission and peer review processes, including consent and potential confidentiality issues will be discussed in detail with each journal by teleconference and/or face to face prior to the journal agreeing to take part to ensure that randomisation of manuscripts is feasible.

In participating journals, the external researcher (BS) will check at least twice a week (by screening automated submission lists) all research manuscripts that are sent out for external peer review. As soon as the first invited peer reviewer accepts the invitation to review, the manuscript will be randomised to the intervention or control arm (see “Randomisation” for more details). It is possible that this process might be slightly different amongst different included journals (e.g. that team members of a journal might be involved in the screening if limited or no access to the journal’s editorial system is granted).

Interventions

Control group: Usual practice

After accepting to review a manuscript, peer reviewers will receive the automated, journal specific standard email with general information as per each journal’s usual practice (e.g. where to access the manuscript, date the peer review report is due).

Intervention group: C-short plus usual practice

After accepting to review a manuscript, peer reviewers will receive the automated, journal specific standard email with general information (identical to control group). In addition, peer reviewers will receive an additional email from the editorial office that includes a short version of the CONSORT checklist (C-short) together with a brief explanation of the items either as a

For peer review only - http://bmjopen.bmj.com/site/about/guidelines.xhtml

table within the email or as an attachment - based on the preferences and possibilities of the journal (Table 1, appendix 1). Peer reviewers will be asked to check whether the items in the C-short checklist are addressed in the manuscript and to request authors to include these items if they are not adequately reported. This second email (see appendix 1), containing the C-short checklist together with a brief explanation, is not generated automatically within the existing journal editorial systems (e.g. ScholarOne or Editorial Manager); it will be sent manually by a researcher (BS) from the journal's editorial system or by a member of the journal's staff. In both cases the email will appear to have come from the editorial office (not the researcher).

Development of the C-short checklist and explanation of items

For the development of C-short we chose the 10 most important and poorly reported CONSORT items as identified by a group of CONSORT experts in a previous study conducted by Hopewell and colleagues.[30] The selection of the items was based on expert opinion and empirical evidence whenever available.[30] In addition, to enable peer reviewers to better understand the items, we added a short explanation for each of the 10 items. These short explanations were extracted and amended from the CONSORT explanation and elaboration paper[11] and from COBWEB which is an online writing aid tool.[33] The short explanation was discussed and adapted by the scientific committee.

Outcomes

Primary outcome

The primary outcome of this study will be the difference in the mean proportion of adequately reported C-short items in published articles between the two groups.

Secondary outcomes

Secondary outcomes will include the following:

For peer review only - http://bmjopen.bmj.com/site/about/guidelines.xhtml

- Mean proportion of adequately reported C-short items in published articles considering each item separately.
- Difference in mean proportion of adequately reported C-short items in published articles considering each sub-item (see "Assessment of outcomes") as a separate item.
- Time from assigning an editor to the first decision (as communicated to the author after the first round of peer-review).
- Proportion of manuscripts rejected after the first round of peer review.
- Proportion of manuscripts that will be published in the journal under study.

<u>Additional outcomes:</u>

- Exploratory analysis of available peer reviewer comments (i.e. any references to CONSORT).

For journals where peer reviewers' comments are subsequently published alongside the published article, we will examine the peer reviewers' comments for any reference to CONSORT and trial reporting. We will contact those journals which do not make peer reviewers' comments publicly available, to see if reviews could be provided for such analyses under the condition that only anonymised data will be published.

<u>Assessment of outcomes:</u>

The outcomes will be assessed independently by two (blinded or at least partially blinded; see "blinding") outcome assessors with expertise in the design and reporting of clinical trials. Any disagreement will be resolved by consensus or if necessary by consulting a third assessor. To ensure consistency between reviewers, we will first pilot the data extraction form; any disparities in the interpretation will be discussed and the data extraction form will be modified accordingly.

For peer review only

For peer review only - http://bmjopen.bmj.com/site/about/guidelines.xhtml

Adequate reporting of items will be assessed in duplicate from published full-text publications following the same instructions as provided by the CONSORT C-short checklist.[11] The following checklist items have, due to their complexity, sub-items which will be extracted separately. The sub-items are highlighted in the short explanation of the intervention (see Table 1 and appendix 1):

- Outcomes (item 6a): (i) Define primary outcome, (ii) how it was measured, (iii) at what time point, and (iv) the analysis metric (e.g. change from baseline, final value).
- Sample size (item 7a): (i) The estimated outcomes in each group, (ii) the α (type I) error level, (iii) the statistical power (or the β (type II) error level), (iv) for continuous outcomes, the standard deviation of the measurements
- Blinding (item 11a): Is the blinding status clear for the following persons: (i) Healthcare provider, (ii) patients, and (iii) outcome assessors.
- Funding (item 25): (i) The funding source, and (ii) the role of funder in the design, conduct, analysis, and reporting.

All items will be judged as either "yes" meaning adequately reported, "no" meaning not adequately reported or not reported at all, or "NA" meaning that this sub-item is not applicable for this RCT. Items with different sub-items will only be judged as adequately reported if all relevant sub-items were adequately reported.

The outcomes "time from assigning an editor to the first decision", "proportion of manuscripts rejected after the first round of peer-review", and "proportion of manuscripts that will be published in the journal under study" will be extracted directly from the journal's editorial system or provided by the journal.

Participant timeline

The overview of the study schedule, including enrolment, intervention and assessments is presented in Table 2.

For peer review only - http://bmjopen.bmj.com/site/about/guidelines.xhtml

Sample size

For the sample size calculation, we hypothesised in a first scenario (Table 3) that the intervention C-short will result in a 25% relative increase in adequate reporting compared to the control (meaning that 70% of items will be adequately reported in the intervention group and 56% in the control group). This is based on a proportion of adequate reporting of 0.56 for the 10 most important and poorly reported items found in the control group of a previous study (meaning that a mean of 56% of the 10 most important and poorly reported items were reported).[30] The standard deviation (SD) in the same study was 0.23. However, we calculated our sample size to account for a slightly larger variability in our data (SD = 0.25). To demonstrate a significant difference with a power of 90% and a type 1 error at 5%, a total of 136 published articles will be required in this scenario (68 per treatment arm; based on a two sided t-test).

Two authors of this protocol, working for *PLOS ONE* (IP and AC), one of the participating journals, pointed out that 3 out of the 10 assessed items (i.e. item “Registration”, “Protocol”, and “Funding”) should always be implemented in submissions to their journal given their policy requirements for clinical trials. Assuming that this journal will recruit a high proportion of manuscripts, and that also other journals might update their templates, we increased the sample size in a second scenario, in which all these 3 items would have an overall adherence of 90% in the control arm (Table 3). This would entail an overall baseline adherence with the 10 C-short items of 71%. Based on a two sided t-test, a sample size of 166 (83 per treatment arm) will have a power of 80% to find a 15% relative increase (71% adherence in control group; 82% adherence in intervention group; SD = 0.25; a type 1 error at 5%).

Since the final sample size will be based on the number of articles published, rather than on the number of manuscripts randomised, eligible manuscripts will be randomised until 83 articles are published in each arm (resulting in no less than 166 articles), to avoid loss of power due to potential imbalance between arms. Recruitment will be stopped as soon as both arms

For peer review only - http://bmjopen.bmj.com/site/about/guidelines.xhtml

reach the sample size of 83. After recruitment has stopped we will wait three months so that manuscripts, which are still in production, can be published. Manuscripts which are published after the three month period will be excluded

Randomisation and blinding

Manuscripts meeting the eligibility criteria and sent out for external peer review by the journals will be randomised into one of the two groups (allocation 1:1). The randomisation list will be created by the Study-Randomizer© system[34] using random block sizes between 2 and 8 and stratified by journal. As soon as the first peer reviewer accepts the invitation, the manuscript will be included and randomised to one of the two study arms. One of the investigators (BS) will log onto the Study-Randomizer© system[34] and enter the study identification number (ID; provided by the journal), the study title, and the journal the study was submitted to. Subsequently, all additional peer reviewers accepting the invitation to review the same manuscript will receive the same group assignment as the first peer reviewer.

For peer review only

Authors will be blinded to the intervention. Editors will not be actively informed about the randomisation (possible exception listed under “Interventions”). To avoid potential bias, peer reviewers and manuscript authors will not be informed of the study hypothesis, design and intervention.

Outcomes will be assessed in duplicate (see “Assessment of outcomes”). At least one outcome assessor will be blinded. Due to restricted resources the investigator conducting the randomisation (BS) might be involved in the data-extraction from published manuscripts.

Data analysis

For peer review only - http://bmjopen.bmj.com/site/about/guidelines.xhtml

All quantitative variables will be described using means and standard deviations, or medians and interquartile ranges in case severe departures from a normal distribution are identified. Data distributions will be inspected visually (i.e. by histograms) instead of performing formal statistical tests for normality. Categorical variables will be described using frequencies and percentages. For the primary and secondary outcomes, we will estimate the mean difference between the two groups and report them with respective 95% confidence intervals. No interim analysis will be conducted.

Populations of analysis

The main population for analysis will be all manuscripts randomised and accepted for publication in the participating journals. In contrast to RCTs conducted with patients, where losses to follow-up need to be carefully considered (e.g. multiple imputation of missing data), we are only interested in the reporting adherence of RCTs that are published. As such, we will exclude randomised manuscripts that were not published from the main analysis. All outcomes will be calculated based on the main population. The secondary outcome “Time to the first decision”, will additionally be calculated considering all randomised manuscripts (including the ones which were not published). For all analyses a p-value of 0.05 (5% significance level) will be used to indicate statistical significance. Exact p-values will be presented up to three decimal places. We anticipate there will be no missing data in this study, neither at the individual C-short items, nor at the manuscript level. This is due to the study design, which will include only the randomised manuscripts that are accepted for publication. We will analyse if the rate of manuscripts rejected after the first round of peer-review and if the proportion of manuscripts that will be published differentiate amongst the two study arms (both secondary results).

For peer review only - http://bmjopen.bmj.com/site/about/guidelines.xhtml

Analysis of primary endpoint

The effect of the intervention will be estimated as the mean difference in the proportion of C-short items adequately reported between the study arms. If the data on the primary outcome is normally distributed, groups will be compared using an unpaired Student's t-test. If the data is not normally distributed, comparisons will be performed using a non-parametric equivalent test (i.e. Wilcoxon-Mann-Whitney test).

Analysis of secondary endpoints

To investigate the effect of the intervention on the secondary outcomes, mean differences with respective 95% confidence intervals will be reported. If normality is not observed for any of the continuous secondary outcomes, the same strategy adopted for the primary outcome (use of a non-parametric equivalent to the Student's *t*-test) will be used.

Pre-specified subgroup analysis

No formal subgroup comparative analysis is planned for the primary or secondary outcomes. However, the effect of the intervention on the primary outcome within subgroups will be presented using forest plots to visually examine whether it may differ according to some variables, such as: (1) Journals that actively implement the CONSORT Statement (defined as requiring authors to submit a completed CONSORT checklist alongside their manuscript) vs. journals that are not actively implementing the CONSORT Statement; (2) sample size of included RCTs (n < 100 vs. n ≥ 100); and (3) impact factor (<5, 5.1-10; >10) as there is evidence that higher impact factor as well as higher sample size are associated with higher adherence to reporting guidelines.[35] Sub-group analysis at the journal level will only be conducted when sufficient journals are in each group so that no results of individual journals are revealed. All analyses will be exploratory, with the aim of supporting new hypothesis generation, rather than being conclusive.

For peer review only - http://bmjopen.bmj.com/site/about/guidelines.xhtml

Data management and confidentiality

Outcomes from publications will be assessed and extracted in duplicate. Since this information is not confidential, we will use freely available online forms (e.g. Google forms) for data extraction from published RCTs. Data entered will be validated for completeness.

Data from the journal's editorial system (e.g. title of manuscript, first author, randomisation ID, journal, date when manuscript was assigned to an editor, date when the final decision was made, final editorial decision, number of peer reviewers who reviewed the manuscript, the peer review reports [if available]) will be extracted (by BS or a member of the journal's staff), anonymised and entered in password protected files which are saved on a server from the University of Oxford. Data will be managed and curated according to University of Oxford regulations, which includes regular back-up (on a daily basis) of the virtual drives where the data are stored. No auditing or data monitoring is planned (as outcomes are directly extracted from journal's editorial system or in duplicate from published RCTs).

The raw data extracted from the included published manuscripts can be made openly accessible in an anonymised way (i.e. giving the included RCT a number instead of identifying them). Derived/aggregated data, including anonymised information generated from the journal's editorial system, will be stored and made available to the research community when the project ends (see also "Publication policy and access to data"). Where appropriate, the researcher who has access to the journal's editorial system (BS) and anyone else who will see the identifiable data will sign a confidentially agreement with the participating journals, confirming that they will not share identifiable data with any other party. Publishers such as the BMJ state in their Company Privacy Statement that reviews and manuscripts may be used for quality improvement purposes and that is the nature of this research. Furthermore, peer reviewers for all BMJ journals receive the following statement in their invitation letter "*We are constantly trying to find ways of improving the peer review system and have an ongoing*

For peer review only - http://bmjopen.bmj.com/site/about/guidelines.xhtml

programme of research. If you do not wish your review entered into a study please let us know by emailing […] as soon as possible."

Trial registration

This trial was denied registration on ClinicalTrials.gov as the study is not a clinical study that assesses a health outcome in human subjects. Instead we registered the trial on the Open Science Framework (https://osf.io/c4hn8). The first manuscript was randomised in July 2019. We expect that recruitment will be finished in summer 2021.

Patient and public involvement

Given the specific study topic, the steering committee agreed that patient or public involvement is not needed for this study.

Discussion

RCTs are the current gold standard for evaluating any new intervention in evidence-based medicine. Unfortunately, not all RCTs are of high quality. In fact, there are several well-known shortcomings with respect to reporting.[3 15-20] It is important to note that adhering to the CONSORT Statement does not mean that the study is of high quality. However, reporting all items from the CONSORT checklist will enable readers to adequately judge the quality of RCTs.

In this RCT we will test if a simple intervention in the form of asking peer reviewers to check whether selected CONSORT items are adequately addressed will increase the proportion of

For peer review only - http://bmjopen.bmj.com/site/about/guidelines.xhtml

reporting completeness in the published RCTs in the participating journals. A multicentre parallel arm RCT with randomisation at the individual manuscript level was chosen instead of a cluster RCT because the risk of "contamination" at journal level was judged as low as the intervention will be implemented by an external researcher (i.e. BS) or a member of the journal staff (e.g. personnel from Editorial services). The likelihood of contamination due to peer reviewers being invited to assess several RCTs and therefore becoming exposed to both intervention arms was judged small and therefore we do not plan to adjust for clustering by journal. Originally we planned to implement the intervention within the original instruction to peer reviewer email which is sent out as soon as a peer reviewer accepts the invitation from the journal. However, as these emails are sent automatically by the journal's editorial system we would have needed to modify the software from each journal to make sure that only half of the manuscripts administered the intervention. After our first discussion with journal editors and journal staff, we realised that this approach is not feasible and therefore decided to implement the intervention in the form of a separate email. We intended to conduct this RCT in a pragmatic way so that results "would also be relevant to […] people who decide whether to implement the intervention on the basis of its results".[36] Hence we chose to assess outcomes from published articles and not from manuscripts after the first round of revisions. Ideally, the full impact of the intervention would also be measured including all versions of randomised manuscripts in the final statistical analysis. However, due to confidentiality issues and limited resources we will not be able to evaluate manuscript versions prior to publication.

A selection of CONSORT items was chosen instead of the entire CONSORT checklist as we did not want to put too high a burden on peer reviewers, which could increase the risk that peer reviewers ignore our reminder.

For peer review only - http://bmjopen.bmj.com/site/about/guidelines.xhtml

Should the proposed intervention be successful in improving the reporting quality of published RCTs, as measured by the adherence to CONSORT, the intervention could be implemented at the journal level without requiring a large amount of additional resources. In addition, very similar interventions for other article types (e.g. systematic reviews, trial protocols) and corresponding guidelines (e.g. PRISMA, SPIRIT) could be easily implemented too.

Ethics and dissemination

Ethical approval has been obtained from the Medical Sciences Interdivisional Research Ethics Committee of the University of Oxford (R62779/RE001). The original approved study protocol is available in Appendix 2. The WHO Trial Registration Data Set is available in Appendix 3.

The results from this study will be published in a peer reviewed journal irrespective of the study results. Authorship of publications will be granted according to the criteria of the International Committee of Medical Journal Editors (ICMJE). We plan to make the anonymised dataset, including the data from the published articles, available as a supplementary file of the main publication.

For peer review only

For peer review only - http://bmjopen.bmj.com/site/about/guidelines.xhtml

Full references

1. Duley L, Antman K, Arena J, et al. Specific barriers to the conduct of randomized trials. *Clin Trials* 2008;5(1):40-8. doi: 10.1177/1740774507087704
2. Collins R, MacMahon S. Reliable assessment of the effects of treatment on mortality and major morbidity, I: clinical trials. *Lancet* 2001;357(9253):373-80. doi: 10.1016/S0140-6736(00)03651-5
3. Chalmers I. Unbiased, relevant, and reliable assessments in health care: important progress during the past century, but plenty of scope for doing better. *BMJ* 1998;317(7167):1167-8.
4. Altman DG, Simera I, Hoey J, et al. EQUATOR: reporting guidelines for health research. *Lancet* 2008;371(9619):1149-50. doi: 10.1016/S0140-6736(08)60505-X
5. Simera I. EQUATOR Network collates resources for good research. *BMJ* 2008;337:a2471. doi: 10.1136/bmj.a2471
6. Simera I, Altman DG. Writing a research article that is "fit for purpose": EQUATOR Network and reporting guidelines. *Evid Based Med* 2009;14(5):132-4. doi: 10.1136/ebm.14.5.132
7. Simera I, Altman DG, Moher D, et al. Guidelines for reporting health research: the EQUATOR network's survey of guideline authors. *PLoS Med* 2008;5(6):e139. doi: 10.1371/journal.pmed.0050139
8. Simera I, Moher D, Hirst A, et al. Transparent and accurate reporting increases reliability, utility, and impact of your research: reporting guidelines and the EQUATOR Network. *BMC Med* 2010;8:24. doi: 10.1186/1741-7015-8-24
9. Simera I, Moher D, Hoey J, et al. The EQUATOR Network and reporting guidelines: Helping to achieve high standards in reporting health research studies. *Maturitas* 2009;63(1):4-6. doi: 10.1016/j.maturitas.2009.03.011
10. Altman DG, Simera I. A history of the evolution of guidelines for reporting medical research: the long road to the EQUATOR Network. *J R Soc Med* 2016;109(2):67-77. doi: 10.1177/0141076815625599 [published Online First: 2016/02/18]
11. Moher D, Hopewell S, Schulz KF, et al. CONSORT 2010 explanation and elaboration: updated guidelines for reporting parallel group randomised trials. *BMJ* 2010;340:c869. doi: 10.1136/bmj.c869
12. Schulz KF, Altman DG, Moher D, et al. CONSORT 2010 Statement: updated guidelines for reporting parallel group randomised trials. *BMC Med* 2010;8:18. doi: 10.1186/1741-7015-8-18
13. Schulz KF, Altman DG, Moher D, et al. CONSORT 2010 statement: updated guidelines for reporting parallel group randomised trials. *BMJ* 2010;340:c332. doi: 10.1136/bmj.c332
14. CONSORT Transparent Reporting of Trials. Endorsers. Journals and Organizations. http://www.consort-statement.org/about-consort/endorsers [accessed: 27 February 2019]
15. Chan AW, Hrobjartsson A, Haahr MT, et al. Empirical evidence for selective reporting of outcomes in randomized trials: comparison of protocols to published articles. *JAMA* 2004;291(20):2457-65. doi: 10.1001/jama.291.20.2457
16. Chalmers I, Glasziou P. Avoidable waste in the production and reporting of research evidence. *Lancet* 2009;374(9683):86-9. doi: 10.1016/S0140-6736(09)60329-9
17. Ioannidis JP, Lau J. Completeness of safety reporting in randomized trials: an evaluation of 7 medical areas. *JAMA* 2001;285(4):437-43.
18. Glasziou P, Meats E, Heneghan C, et al. What is missing from descriptions of treatment in trials and reviews? *BMJ* 2008;336(7659):1472-4. doi: 10.1136/bmj.39590.732037.47 [published Online First: 2008/06/28]

For peer review only - http://bmjopen.bmj.com/site/about/guidelines.xhtml

19. Boutron I, Dutton S, Ravaud P, et al. Reporting and interpretation of randomized controlled trials with statistically nonsignificant results for primary outcomes. *JAMA* 2010;303(20):2058-64. doi: 10.1001/jama.2010.651
20. Hopewell S, Dutton S, Yu LM, et al. The quality of reports of randomised trials in 2000 and 2006: comparative study of articles indexed in PubMed. *BMJ* 2010;340:c723. doi: 10.1136/bmj.c723
21. Turner L, Shamseer L, Altman DG, et al. Does use of the CONSORT Statement impact the completeness of reporting of randomised controlled trials published in medical journals? A Cochrane review. *Syst Rev* 2012;1:60. doi: 10.1186/2046-4053-1-60
22. Odutayo A, Emdin CA, Hsiao AJ, et al. Association between trial registration and positive study findings: cross sectional study (Epidemiological Study of Randomized Trials-ESORT). *BMJ* 2017;356:j917. doi: 10.1136/bmj.j917 [published Online First: 2017/03/16]
23. Shamseer L, Hopewell S, Altman DG, et al. Update on the endorsement of CONSORT by high impact factor journals: a survey of journal "Instructions to Authors" in 2014. *Trials* 2016;17(1):301. doi: 10.1186/s13063-016-1408-z [published Online First: 2016/06/28]
24. Hopewell S, Altman DG, Moher D, et al. Endorsement of the CONSORT Statement by high impact factor medical journals: a survey of journal editors and journal 'Instructions to Authors'. *Trials* 2008;9:20. doi: 10.1186/1745-6215-9-20 [published Online First: 2008/04/22]
25. Hopewell S, Ravaud P, Baron G, et al. Effect of editors' implementation of CONSORT guidelines on the reporting of abstracts in high impact medical journals: interrupted time series analysis. *BMJ* 2012;344:e4178. doi: 10.1136/bmj.e4178 [published Online First: 2012/06/26]
26. Blanco D, Altman D, Moher D, et al. Scoping review on interventions to improve adherence to reporting guidelines in health research. *BMJ Open* 2019;9(5):e026589. doi: 10.1136/bmjopen-2018-026589 [published Online First: 2019/05/12]
27. Hopewell S, Collins GS, Boutron I, et al. Impact of peer review on reports of randomised trials published in open peer review journals: retrospective before and after study. *BMJ* 2014;349:g4145. doi: 10.1136/bmj.g4145
28. Cobo E, Selva-O'Callagham A, Ribera JM, et al. Statistical reviewers improve reporting in biomedical articles: a randomized trial. *PLoS One* 2007;2(3):e332. doi: 10.1371/journal.pone.0000332 [published Online First: 2007/03/29]
29. Cobo E, Cortes J, Ribera JM, et al. Effect of using reporting guidelines during peer review on quality of final manuscripts submitted to a biomedical journal: masked randomised trial. *Bmj* 2011;343:d6783. doi: 10.1136/bmj.d6783 [published Online First: 2011/11/24]
30. Hopewell S, Boutron I, Altman DG, et al. Impact of a web-based tool (WebCONSORT) to improve the reporting of randomised trials: results of a randomised controlled trial. *BMC Med* 2016;14(1):199. doi: 10.1186/s12916-016-0736-x
31. Glonti K, Cauchi D, Cobo E, et al. A scoping review on the roles and tasks of peer reviewers in the manuscript review process in biomedical journals. *BMC Med* 2019;17(1):118. doi: 10.1186/s12916-019-1347-0 [published Online First: 2019/06/21]
32. Chan AW, Tetzlaff JM, Gotzsche PC, et al. SPIRIT 2013 explanation and elaboration: guidance for protocols of clinical trials. *BMJ* 2013;346:e7586. doi: 10.1136/bmj.e7586
33. Barnes C, Boutron I, Giraudeau B, et al. Impact of an online writing aid tool for writing a randomized trial report: the COBWEB (Consort-based WEB tool) randomized controlled trial. *BMC Med* 2015;13:221. doi: 10.1186/s12916-015-0460-y [published Online First: 2015/09/16]
34. Study Randomizer. https://studyrandomizer.com/ [accessed: 8. July 2019].
35. Jin Y, Sanger N, Shams I, et al. Does the medical literature remain inadequately described despite having reporting guidelines for 21 years? - A systematic review of

For peer review only - http://bmjopen.bmj.com/site/about/guidelines.xhtml

reviews: an update. *Journal of multidisciplinary healthcare* 2018;11:495-510. doi: 10.2147/jmdh.S155103 [published Online First: 2018/10/13]
36. Loudon K, Treweek S, Sullivan F, et al. The PRECIS-2 tool: designing trials that are fit for purpose. *BMJ* 2015;350:h2147. doi: 10.1136/bmj.h2147 [published Online First: 2015/05/10]

For peer review only

For peer review only - http://bmjopen.bmj.com/site/about/guidelines.xhtml

Authors' contributions

SH, BS, IB, MB, DM, and PR had the study idea and designed the study. SS, IP and AC provided expertise to ensure implementation at the journal level was possible. MMS was responsible for statistical aspects, including the sample size calculation and the data analysis plan. BS and SH wrote the first draft of the study protocol. All authors critically revised the protocol and approved the final version.

Funding statement: Benjamin Speich is supported by an Advanced Postdoc.Mobility grant from the Swiss National Science Foundation (P300PB_177933). David Moher is supported by a University Research Chair, University of Ottawa. Michael M Schlussel is funded by Cancer Research UK. The funders had no role in designing the study and will also have no role in conducting the study, or analysing and reporting study results.

Competing interests statement

SS is employed by the British Medical Journal (BMJ). IP and AC are employed by the Public Library of Science (PLOS). DM, SH, and IB are members of the CONSORT executive and authors of the CONSORT 2010 Statement. DM and PR are members of the EQUATOR network steering group. MMS is a meta-researcher and reporting guideline developer, enthusiast, and disseminators, he may therefore overestimate the importance of this project. All authors have declared that no other competing interests exist.

Roles and responsibilities

The principal investigator (BS) is responsible for the preparation and the revisions of the study protocol, organising meetings of the steering committee, recruiting and randomising eligible manuscripts as well as the publication of study reports. The steering committee (IB, MB, SH, DM, PR, BS, MMS, and SS) is responsible for revising the protocol, defining and validating the

For peer review only - http://bmjopen.bmj.com/site/about/guidelines.xhtml

additional short explanation for each CONSORT item, advising on study implementation, and for publishing the results of this study. MMS is responsible for the sample size calculation and the statistical analyses.

Word count: 4408

For peer review only - http://bmjopen.bmj.com/site/about/guidelines.xhtml

632 **Figure legend**

633

634 **Figure 1:** Study flowchart

635

636

For peer review only

For peer review only - http://bmjopen.bmj.com/site/about/guidelines.xhtml

Table 1: The ten most important and poorly reported CONSORT items as defined by a group of experts on the CONSORT statement.[30] For better understanding key features were summarised within a short explanation (extracted from the CONSORT explanation and elaboration paper[11] as well as from the COBWEB tool[33]).

Item	Section	CONSORT item	Short explanation
1	Outcomes (6a)	Completely defined pre-specified primary outcome measure, including how and when it was assessed	Is it clear (1) what the primary outcome is (usually the one used in the sample size calculation), (2) how it was measured (if relevant; e.g. which score used), (3) at what time point, and (4) what the analysis metric was (e.g. change from baseline, final value)?
2	Sample size (7a)	How sample size was determined	Is there a clear description of how the sample size was determined, including (1) the estimated outcomes in each group; (2) the α (type I) error level; (3) the statistical power (or the β (type II) error level); and (4) for continuous outcomes, the standard deviation of the measurements?
3	Sequence generation (8a)	Method used to generate random allocation sequence	Does the description make it clear if the “assigned intervention is determined by a chance process and cannot be predicted”?
4	Allocation concealment (9)	Mechanism used to implement random allocation sequence (such as sequentially numbered containers), describing any steps taken to conceal the sequence until interventions were assigned	Is it clear how the care provider enrolling participants was made ignorant of the next assignment in the sequence (different from blinding)? Possible methods can rely on centralised or “third-party” assignment (i.e., use of a central telephone randomisation system, automated assignment system, sealed containers).
5	Blinding (11a)	If done, who was blinded after assignment to interventions (for example, participants, care providers, those assessing outcomes)	Is it clear if (1) healthcare providers, (2) patients, and (3) outcome assessors are blinded to the intervention? General terms such as “double-blind” without further specifications should be avoided.
6	Outcomes and estimation (17a/b)	For the primary outcome, results for each group, and the estimated effect size and its precision (such as 95% confidence intervals)	Is the estimated effect size and its precision (such as standard deviation or 95% confidence intervals) for each treatment arm reported? When the primary outcome is binary, both the relative effect (risk ratio, relative risk) or odds ratio) and the absolute effect (risk difference) should be reported with confidence intervals.
7	Harms (19)	All-important harms or unintended effects in each group	Is the number of affected persons in each group, the severity grade (if relevant) and the absolute risk (e.g. frequency of incidence) reported? Are the number of serious, life threatening events and deaths reported? If no adverse event occurred this should be clearly stated.
8	Registration (23)	Registration number and name of trial registry	Is the registry and the registration number reported? If the trial was not registered, it should be explained why.
9	Protocol (24)	Where trial protocol can be accessed	Is it stated where the trial protocol can be assessed (e.g. published, supplementary file, repository, directly from author, confidential and therefore not available)?
10	Funding (25)	Sources of funding and other support (such as supply of drugs) and role of funders	Are (1) the funding sources, and (2) the role of the funder(s) described?

For peer review only - http://bmjopen.bmj.com/site/about/guidelines.xhtml

Table 2: Study schedule

	Enrolment	Allocation and intervention	Intervention	Post-intervention	
Time-point	*Studies which are sent out for peer-review*	*After first peer-reviewer accepts invitation*	*Whenever an additional peer-reviewer accepts invitation*	*First decision by journal*	*Published manuscripts*
Eligibility screen	X				
Allocation		X			
Intervention:					
C-short + usual care		X	X		
Usual care		X	X		
Assessment of trial characteristics:					
Funding source					X
Study centres (single centre or multicentre)					X
Sample size					X
Study design (e.g. parallel arm, crossover)					X
Hypothesis (e.g. superiority, non-inferiority)					X
Medical field					X
Intervention tested					X
Number of trial arms					X
Number of peer-reviewers					X
Journal which published the manuscript					X
Number of journals requesting CONSORT adherence (submission of checklist mandatory)					X
Assessment of outcomes:					
Time from assigning an academic editor until the first decision				X	
Proportion of manuscripts directly rejected after the first round of peer-review				X	
Proportion of manuscripts that will be published in the journal under study					X
Adherence to CONSORT items and sub-items					X

For peer review only

For peer review only - http://bmjopen.bmj.com/site/about/guidelines.xhtml

644 **Table 3:** Assumptions for sample size calculations in two different scenarios.

Item	CONSORT item	Scenario 1. Adequate reporting as published in WebCONSORT [30]	Scenario 2. Adapted from Scenario 1
1	Outcomes (6a)	77% (79 of 103)	77% (79 of 103)
2	Sample size (7a)	83% (85 of 103)	83% (85 of 103)
3	Sequence generation (8a)	76% (78 of 103)	76% (78 of 103)
4	Allocation concealment (9)	55% (57 of 103)	55% (57 of 103)
5	Blinding (11a)	35% (36 of 103)	35% (36 of 103)
6	Outcomes and estimation (17a	44% (45 of 103)	44% (45 of 103)
7	Harms (19)	71% (73 of 103)	71% (73 of 103)
8	Registration (23)	69% (71 of 103)	90%
9	Protocol (24)	19% (20 of 103)	90%
10	Funding (25)	34% (35 of 103)	90%
**Overall**		**56%**	**71%**

645 Abbreviation: CONSORT= CONsolidated Standards for Reporting Trials

646

For peer review only

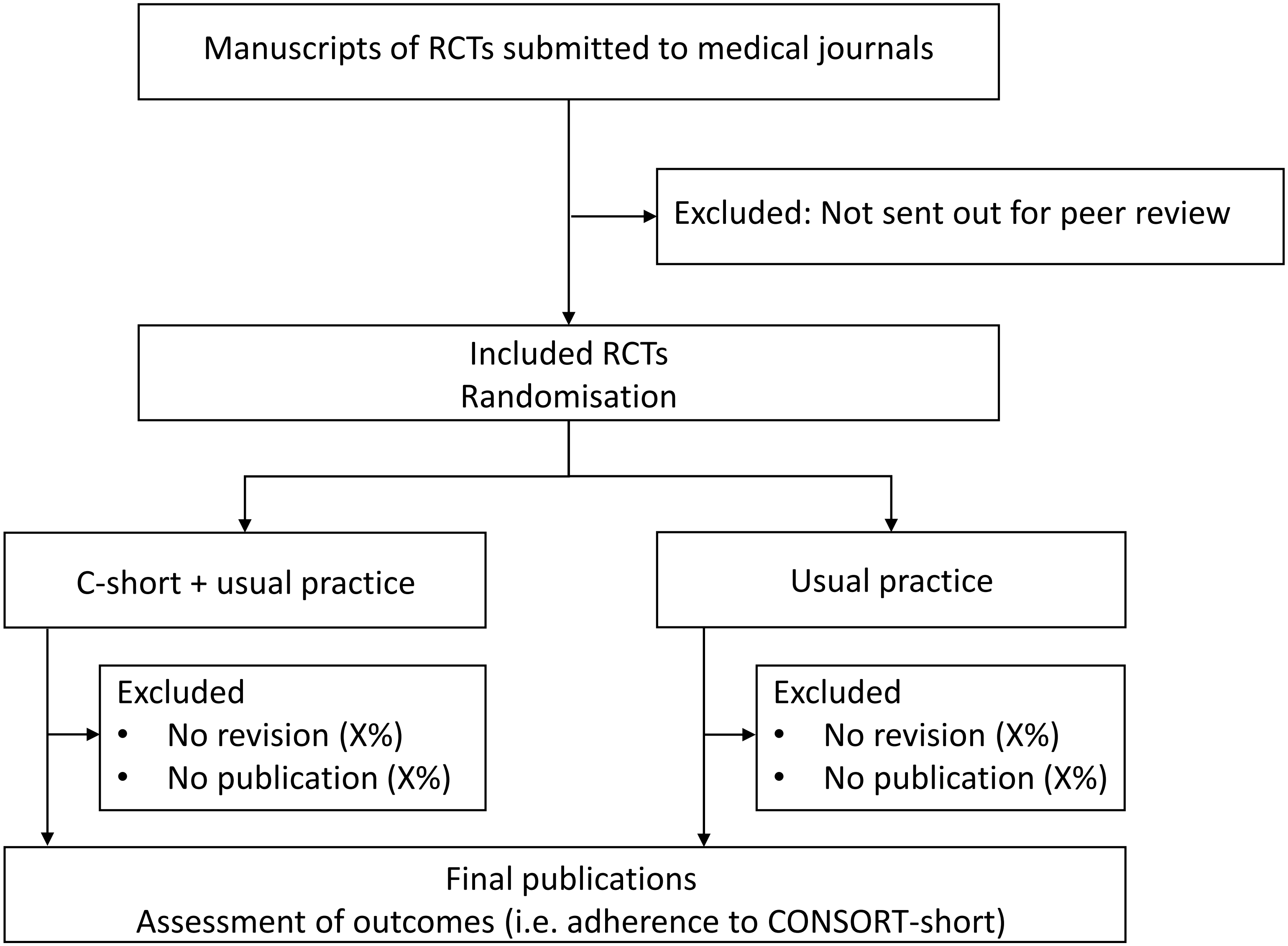
Manuscripts of RCTs submitted to medical journals
Excluded: Not sent out for peer review
Included RCTs
Randomisation
C-short + usual practice
Usual practice
Excluded
No revision (X%)
No publication (X%)
Excluded
No revision (X%)
No publication (X%)
Final publications
Assessment of outcomes (i.e. adherence to CONSORT-short)

Impact of a short version of the CONSORT checklist for peer reviewers to improve the reporting of randomised controlled trials published in biomedical journals: study protocol for a randomised controlled trial

Benjamin Speich, Sara Schroter, Matthias Briel, David Moher, Iratxe Puebla, Alejandra Clark, Michael M Schlussel, Philippe Ravaud, Isabelle Boutron, Sally Hopewell

Appendix

Appendix 1: Example of the email which will be sent out in the intervention arm (C-Short). The exact wording might be slightly adapted according to the journal preferences.
Page 2

Appendix 2: Original study protocol as it was approved by the Medical Sciences Interdivisional Research Ethics Committee of the University of Oxford (R62779/RE001).
Page 3

Appendix 3: WHO Trial Registration Data Set (Version 1.3.1)
Page 27

For peer review only

For peer review only - http://bmjopen.bmj.com/site/about/guidelines.xhtml

Appendix 1: Example of the email which will be sent out in the intervention arm (C-Short). The exact wording might be slightly adapted according to the journal preferences.

Dear **Title, Name**,

We thank you for accepting to peer-review a manuscript for **journal name**. As we are trying to improve the reporting for randomised controlled trials according to the CONSORT guidelines, we would like to ask if you could check whether the following most important and poorly reported items are adequately implemented as indicated in the *table below/attached table*.

Item	Section	CONSORT item	Short explanation
1	Outcomes (6a)	Completely defined pre-specified primary outcome measure, including how and when they were assessed	Is it clear (1) what the primary outcome is (usually the one used in the sample size calculation), (2) how it was measured (if relevant; e.g. which score used), (3) at what time point, and (4) what the analysis metric was (e.g. change from baseline, final value)?
2	Sample size (7a)	How sample size was determined	Is there a clear description of how the sample size was determined, including (1) the estimated outcomes in each group; (2) the α (type I) error level; (3) the statistical power (or the β (type II) error level); and (4) for continuous outcomes, the standard deviation of the measurements?
3	Sequence generation (8a)	Method used to generate random allocation sequence	Does the description make it clear if the "assigned intervention is determined by a chance process and cannot be predicted"?
4	Allocation concealment (9)	Mechanism used to implement random allocation sequence (such as sequentially numbered containers), describing any steps taken to conceal the sequence until interventions were assigned	Is it clear how the care provider enrolling participants was made ignorant of the next assignment in the sequence (different from blinding)? Possible methods can rely on centralised or "third-party" assignment (i.e., use of a central telephone randomisation system, automated assignment system, sealed containers).
5	Blinding (11a)	If done, who was blinded after assignment to interventions (for example, participants, care providers, those assessing outcomes)	Is it clear if (1) healthcare providers, (2) patients, and (3) outcome assessors are blinded to the intervention? General terms such as "double-blind" without further specifications should be avoided.
6	Outcomes and estimation (17a/b)	For the primary outcome, results for each group, and the estimated effect size and its precision (such as 95% confidence intervals)	Is the estimated effect size and its precision (such as standard deviation or 95% confidence intervals) for each treatment arm reported? When the primary outcome is binary, both the relative effect (risk ratio, relative risk) or odds ratio) and the absolute effect (risk difference) should be reported with confidence intervals.
7	Harms (19)	All-important harms or unintended effects in each group	Is the number of affected persons in each group, the severity grade (if relevant) and the absolute risk (e.g. frequency of incidence) reported? Are the number of serious, life threatening events and deaths reported? If no adverse event occurred this should be clearly stated.
8	Registration (23)	Registration number and name of trial registry	Is the registry and the registration number reported? If the trial was not registered, it should be explained why.
9	Protocol (24)	Where trial protocol can be accessed	Is it stated where the trial protocol can be assessed (e.g. published, supplementary file, repository, directly from author, confidential and therefore not available)?
10	Funding (25)	Sources of funding and other support (such as supply of drugs) and role of funders	Are (1) the funding sources, and (2) the role of funder(s) described?

Your efforts are highly appreciated.

Kind regards,

journal name-Team

For peer review only - http://bmjopen.bmj.com/site/about/guidelines.xhtml

Appendix 2: Original study protocol as it was approved by the Medical Sciences Interdivisional Research Ethics Committee of the University of Oxford (R62779/RE001).

CENTRE for STATISTICS in MEDICINE

UNIVERSITY OF OXFORD

Impact of a short form of the CONSORT checklist for peer reviewers to improve the reporting of randomised controlled trials published in biomedical journals: a randomised controlled trial

Short title: CONSORT for Peer Review (CONSORT-PR)

Principal investigator: Dr Benjamin Speich
Centre for Statistics in Medicine, University of Oxford

Dr Benjamin Speich
Centre for Statistics in Medicine,
Botnar Research Centre, University of Oxford, Windmill Road, Oxford OX3 7LD
Email: Benjamin.speich@ndorms.ox.ac.uk

For peer review only

SCIENTIFIC COMMITTEE

Prof Isabelle Boutron, Paris Descartes University, France
Prof Matthias Briel, University of Basel, Switzerland
Associate Prof Sally Hopewell, Centre for Statistics in Medicine, Oxford University, UK
Prof David Moher, Centre for Journalology, Clinical Epidemiology Program, Ottawa Hospital Research Institute, Canada
Prof Philippe Ravaud, Paris Descartes University, France
Dr Benjamin Speich, Centre for Statistics in Medicine, University of Oxford, UK
Dr. Michael M Schlussel, Centre for Statistics in Medicine, University of Oxford, UK

For peer review only - http://bmjopen.bmj.com/site/about/guidelines.xhtml

Dr Sara Schroter, The BMJ, London, UK

Trial registration: This trial will be prospectively registered under clinicaltrials.gov.

Protocol version: Version 1.1 2019-05-21

Funding: Benjamin Speich is supported by an Advanced Postdoc.Mobility grant from the Swiss National Science Foundation (P300PB_177933). David Moher is supported by a University Research Chair, Ottawa. The funders had no role in designing the study and will also have no role in conducting the study as well as in analysing and reporting study results.

Roles and responsibilities:

Contributors: SH, BS, IB, MB, DM, PR, had the study idea and designed the study. SS provided expertise to ensure implementation at the journal level was possible. MMS was responsible for statistical aspects, including the sample size calculation and the data analysis plan. BS and SH wrote the first draft of the study protocol. All authors critically revised the protocol and approved the final version.

Sponsor and contact information: Centre for Statistics in Medicine, Botnar Research Centre, University of Oxford, Windmill Road, Oxford OX3 7LD. Principal investigator: Benjamin Speich (Email: Benjamin.speich@ndorms.ox.ac.uk)

Sponsor and funders: The funders had no role in designing the study and will also have no role in conducting the study as well as in analysing and reporting study results.

Roles and responsibilities: The principal investigator (BS) is responsible for the preparation and the revisions of the study protocol, organising meetings of the steering committee, recruiting and randomizing eligible manuscripts as well as the publication of study reports. The steering committee (IB, MB, SH, DM, PR, BS, MMS, and SS) is in charge of participating in the elaboration of the protocol, defining and validating the additional short explanation for each CONSORT item, following the evolution of the committed study and for publishing the results of this study. MMS is responsible for the sample size calculation and the statistical analyses.

For peer review only - http://bmjopen.bmj.com/site/about/guidelines.xhtml

Table of content:

1. BACKGROUND AND RATIONAL 6
1.1 Need for clinical research and epidemiologic transparency 6
1.2 Transparency in published randomised controlled trials 6
1.3 Journal attempts to improve reporting in published randomised controlled trials 7
2. HYPOTHESIS 8
3. OBJECTIVE 8
3.1 Main objective 8
4. METHODS 9
4.1 Trial design 9
4.2 Study setting and eligibility criteria 9
4.3 Interventions 10
4.4 Outcomes 13
4.5 Participant timeline 14
4.6. Sample size 16
4.7 Randomisation and blinding 17
4.8 Statistical methods 18
4.8.1 Populations of analysis 18
4.8.2 Data analysis 19
4.8.3 Analysis of primary endpoint 19
4.8.4 Analysis of secondary endpoints 19
5 LEGAL AND GENERAL LOGISTICS 20
5.1. Organisation of study 20
5.1.1 Coordinating centre 20
5.1.2 Scientific committee 20
5.2. Regulatory aspects 21
6 PUBLICATION POLICY AND ACCESS TO DATA 22
REFERENCES 23
APPENDIX 26

For peer review only

For peer review only - http://bmjopen.bmj.com/site/about/guidelines.xhtml

1. Background and rational

1.1 Need for clinical research and epidemiologic transparency

There is substantial agreement that well conducted and reported randomised controlled trials (RCTs) generate the most trustworthy evidence when newly developed or already existing clinical interventions are evaluated (1-3). Besides the complexity and the high associated costs of conducting RCTs (4-6), there are major issues with their reporting that often make it difficult for researchers, clinicians, patients or policymakers to interpret the current evidence on a specific topic (7, 8). Chronologically, the most prominent difficulties in reporting consist of (i) poor reporting in study protocols for RCTs (9-12); (ii) a substantial fraction of trials are not registered, prematurely discontinued (most common due to difficulties with recruitment) and not published (13, 14); and (iii) that published RCTs are often poorly reported (7).

For clinicians, scientists and decision makers, published articles are often the only way to know how a study was conducted. In order to judge the internal and external validity of RCTs, it is crucial that these articles present transparent, accurate and unbiased information about the methods and conduct of the RCT.

1.2 Transparency in published randomised controlled trials

To improve the transparency in clinical and epidemiological research the international organisation called the EQUATOR (Enhancing the Quality and Transparency of Research) Network was founded in 2006 (15-20). This international network consists of researchers, epidemiologists, people in charge of recommendations for the presentation of articles or “reporting guidelines”, statisticians, clinicians and editors from some of the most prestigious journals (e.g., *Lancet, JAMA, Annals of Internal Medicine, BMJ*).

The CONSORT Statement (CONsolidated Standards for Reporting Trials), is perhaps the most important reporting guideline designed to help improve the transparency and quality of reporting of RCTs (21, 22). The CONSORT Statement, consisting of 25 items and a flow diagram which should be reported in papers describing RCTs. The last update of the CONSORT Statement was published simultaneously in 10 leading medical journals in 2010 (23). Currently CONSORT is endorsed by 585 journals (24). The CONSORT Statement guides authors, peer reviewers and journal editors on what information should be included in published reports of RCTs in order to facilitate critical judgment and interpretation of results. It is important to note, that adhering to the CONSORT Statement does not mean that the study is of high quality. However, reporting all items from the CONSORT list will enable readers to adequately judge the quality of RCTs.

For peer review only - http://bmjopen.bmj.com/site/about/guidelines.xhtml

A number of research studies have identified serious limitations in the reporting of published RCTs (3, 25-30). Despite some improvement in reporting following the implementation of the CONSORT Statement, there still remain major reporting deficiencies in published RCTs (31). For example, Odutayo and colleagues showed that a large proportion of RCTs published in December 2012 in PubMed did not define the primary outcome (31%), did not state the sample size calculation (45%) and did not explain the method of allocation concealment (50%) (32). This lack of transparency is a major limiting factor for the reader who assesses an article in order to find the answer to a specific question; it is also a major problem for scientists who perform systematic reviews and meta-analyses. Thus, some published trials may not be included in the meta-analysis because of their lack of transparency. Chan showed (25, 33) that 50% of efficacy outcomes and 65% of safety outcomes could not be included in meta-analyses because of how they were reported. Furthermore, even if these trials are included in systematic reviews and meta-analyses, an adequate risk of bias assessment is often not possible due to the poor reporting quality. Nevertheless, the main consequence of the lack of transparency is the risk of accepting treatments that are ineffective or cause serious adverse events (34).

1.3 Journal attempts to improve reporting in published randomised controlled trials

Journals can play a vital role in improving the reporting of published reports of RCTs. For example, a survey of authors' instructions on journal websites revealed that in 2014 63% (106 of 168) of biomedical journals mentioned CONSORT within their "Instructions to Authors" (35). Of those journals 38 (36%) required a CONSORT checklist as a condition of RCT report submission. Such implementation indicates some improvement over time compared to an assessment in 2007 when only 17 journals requested the CONSORT checklist (36). An interrupted time series analysis which assessed if the CONSORT for Abstracts guideline had an effect on the reporting quality, found that results are better reported in Journals which enforce the policy (37).

In a study published in 2016 authors of RCTs were asked by journal editors to use the web-based CONSORT tool at the manuscript revision stage (38). Authors who were randomly allocated to the intervention had access to a tool which allowed them to combine different CONSORT extensions (according to study design, medical field) to generate customised checklists. In the control group, authors had access to a CONSORT flow diagram generator. The goal was to improve the reporting of CONSORT items with a simple webtool. However, a quarter of all authors either wrongly selected a CONSORT extension or failed to select an extension, indicating that further education is needed in terms of when and how to implement CONSORT extensions.

For peer review only - http://bmjopen.bmj.com/site/about/guidelines.xhtml

A systematic scoping review conducted in 2017 by Blanco and colleagues summarised different interventions aimed to improve adherence to reporting guidelines (39) (manuscript with results currently under review. Draft received via personal communication). A number of different interventions were identified and some had also been tested at journals. However, the interventions, besides requesting submission of checklists from authors, required additional resources at the journal level (e.g. internal peer review by editorial assistants or inviting an additional statistical peer-reviewer (40, 41)). Therefore, it is unlikely that these interventions will be implemented in the vast majority of journals, especially not in smaller journals with limited resources. A study examining “the nature and extent of changes made to manuscripts after peer review, in relation to the reporting of methodological aspects of RCTs” and “the type of changes requested by peer reviewers” found that peer review did lead to some improvement in reporting (40).

Building on these findings we plan to evaluate the impact of inviting peer reviewers to explicitly use a short version of the CONSORT checklist (including a short explanation of those items) as part of their review process. If this intervention deems to be effective, it could be easily implemented by all medical journals without needing additional resources at a journal level.

2. Hypothesis

We propose an RCT to evaluate the impact of asking peer reviewers to use a short version of the CONSORT checklist when reviewing a manuscript of an RCT and whether it improves the completeness of reporting. Our hypothesis is that reminding peer reviewers of the CONSORT items (including a short explanation of those items) will result in higher adherence to CONSORT guidelines in published RCTs. We only selected a limited number of the CONSORT items because we did not want to deter peer reviewers with too much information. Since peer reviewing in general can be burdensome, we felt that this approach is more promising than listing all items, risking that the information will be ignored. The short version of the CONSORT checklist is based on the same items described in a previous study as the 10 most important and underreported CONSORT items (38).

3. Objective

3.1 Main objective

The main objective of this study is to evaluate the impact of asking peer reviewers during the standard peer-review process to ask them to use a short version of the CONSORT checklist (C-short) and whether it will improve the reporting in published RCTs compared to manuscripts where the peer reviewers underwent usual practice.

For peer review only - http://bmjopen.bmj.com/site/about/guidelines.xhtml

4. Methods

4.1 Trial design

This study is a multicentre RCT with articles being the unit of randomisation (Figure 1; allocation ratio 1:1). A multicentre parallel arm RCT with randomisation at the individual article level was chosen instead of a cluster RCT because the risk of any “contamination” on journal level is not given as the intervention will be implemented by an external researcher (i.e. BS). The possibility of contamination due to the possibility that peer reviewer are invited to assess several RCTs and are randomised into both arms was judged as relatively small and therefore we do not plan to adjust for clustering by journal. The journal staff (i.e. editors) will not be actively told which manuscript was allocated to the proposed intervention and which to the control group.

Figure 1: Study flowchart

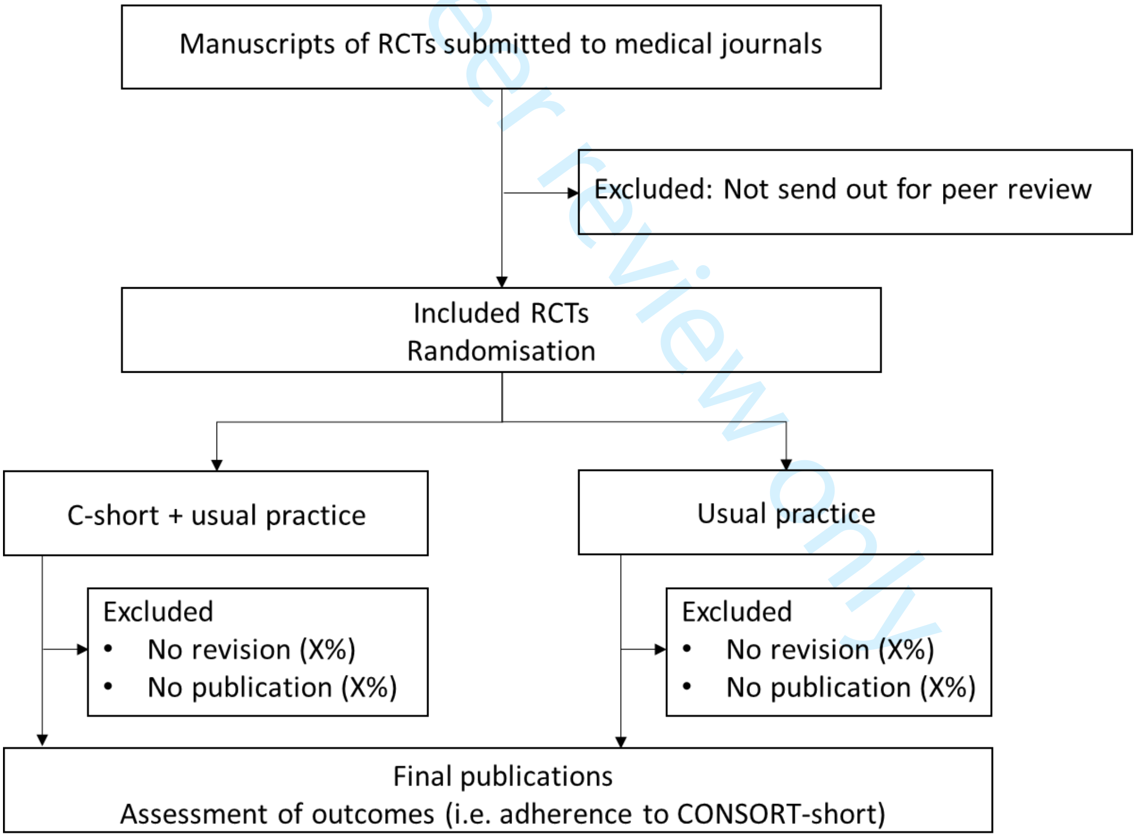

4.2 Study setting and eligibility criteria

The population will be defined on two levels. Included journals and included articles.

Included journals must: i) endorse the CONSORT Statement (e.g. assessed via journals Instruction to Authors); ii) publish primary results of at least five RCTs in 2017 (identified in a brief PubMed search as publishing RCTs in 2017). To be efficient, we plan to contact (via

For peer review only - http://bmjopen.bmj.com/site/about/guidelines.xhtml

email) the editors of eligible journals within a publishing house (i.e. journals which are part of the BMJ series, BMC series, PLoS, Lancet, JAMA) instead of separate journals. A description of the requirements for participation and a short summary information sheet will be included as part of the email invitation sent to journal editors. If a journal is eligible, and agrees to take part, the journal will also need to provide access to their journal editorial system (e.g. ScholarOne, Editorial Manager) to enable the external researcher (i.e. BS) to screen and randomise eligible manuscripts. In cases this is not possible, we will explore with separate journals if it would be possible to grant limited access (e.g. only rights to screen studies) and that the emails from the intervention would be sent by a person from the editorial team.

We will include all submitted manuscripts reporting RCTs for which the journal decides to send out for external peer review. Since the 10 chosen CONSORT checklist items are applicable to different study designs, we will include all RCTs regardless of study design (e.g. parallel group trial, cluster trial, superiority trial, non-inferiority trial). Articles presenting clearly secondary trial results, additional time points, economic analyses, or any other analyses derived from an RCT dataset not including the study's main results will be excluded. Furthermore, RCTs which are clearly labelled as a pilot or feasibility study or randomise animals or cells instead of individuals will be excluded.

Details of journal manuscript submission and peer review processes, including, consent and potential confidentiality issues will be discussed in detail with each journal by teleconference and/or face to face prior to the journal agreeing to take part to ensure that randomisation of manuscripts is feasible. We considered conducting randomisation at the level of the journal (i.e. cluster RCTs). However, in order to make the intervention as easy and simple to implement (and with little or no additional effort from the journal) we believe that randomisation at the manuscript level - with an external researcher implementing the intervention within the existing journal management systems - will be the most efficient study design.

In participating journals, the external investigator (BS) will have access to the editorial management software (e.g. ScholarOne or Editorial Manager) and will check at least twice a week (using automated report lists) all research manuscripts that are sent out for external peer review. As soon as the first peer-reviewer accepts the invitation to review, the manuscript will be randomised to the intervention or control arm (see "Randomisation" for more details). It is possible that this process might be slightly different amongst different included journals.

4.3 Interventions

Experimental group: C-short plus usual practice

For peer review only - http://bmjopen.bmj.com/site/about/guidelines.xhtml

After accepting to review an article, peer reviewers will receive the automated, journal specific standard email with general information as per each journal's usual practice (e.g. where to access the manuscript, date when the peer review report is due). In addition, peer-reviewers who received a manuscript which was randomised to C-short will receive an additional email including a short version of the CONSORT checklist (C-short) (either within the email or a as an attachment; based on the preferences and possibilities of the journal) focusing on the 10 most important and most poorly reported items (Table 1; as previously defined by a group of experts of the CONSORT Group (38)). Peer-reviewers will be asked to pay particular attention to items in the C-short checklist and request authors to report on these items, if not already adequately reported. This second email, containing the C-short checklist, is not generated automatically within the existing journal editorial management system (e.g. ScholarOne or Editorial Manager); it will be sent by the investigator who has access to the journal editorial system (BS). An example of this additional email is presented within the appendix (appendix 1; the exact wording might be changed according to the preferences of the participating journals). At least twice a week the editorial management system will be checked for each journal and if a peer reviewer has accepted an invitation to review, an email containing the C-short intervention will be generated and sent. It might be possible that some journals will only provide the right to access and read manuscripts in the editorial management system, but not to send emails. If this is the case, the corresponding Editor (or designated person within the journal) will be informed to send the emails.

Development and testing of the short explanation of the C-short items:

We chose the 10 most important and poorly reported CONSORT items as identified by a group of CONSORT experts in a previous study conducted by Hopewell and colleagues (38). The selection of the items was based on expert opinion and empirical evidence whenever available (38). In addition, we have added a short explanation for each of the 10 items. These short explanations were extracted and amended from the CONSORT explanation and elaboration paper (21) and from COBWEB which is online writing aid tool (42). The short explanation was discussed and adapted by the scientific committee.

Control group: Usual practice:

After accepting to review an article, peer reviewers will receive the automated, journal specific standard email with general information as per each journal's usual practice (e.g. where to access the manuscript, date until when the peer review report is due). However, they will not receive the second email, sent by the investigator who has access to the journal editorial system (BS) which contains the C-short checklist.

For peer review only - http://bmjopen.bmj.com/site/about/guidelines.xhtml

Table 1: The ten most important and poorly reported CONSORT items as defined by a group of experts on the CONSORT statement (38). For better understanding key features were summarised within a short explanation (extracted from the CONSORT explanation and elaboration paper (21) as well as from the COBWEB tool (42)).

Item	Section	CONSORT item	Short explanation
1	Outcomes (6a)	Completely defined pre-specified primary outcome measure, including how and when they were assessed	Is it clear (1) what the primary outcome is (usually the one used in the sample size calculation), (2) how it was measured (if relevant; e.g. which score used), (3) at what time point, and (4) what the analysis metric was (e.g. change from baseline, final value)?
2	Sample size (7a)	How sample size was determined	Is there a clear description of how the sample size was determined, including (1) the estimated outcomes in each group; (2) the α (type I) error level; (3) the statistical power (or the β (type II) error level); and (4) for continuous outcomes, the standard deviation of the measurements?
3	Sequence generation (8a)	Method used to generate random allocation sequence	Does the description make it clear if the "assigned intervention is determined by a chance process and cannot be predicted"?
4	Allocation concealment (9)	Mechanism used to implement random allocation sequence (such as sequentially numbered containers), describing any steps taken to conceal the sequence until interventions were assigned	Is it clear how the care provider enrolling participants was made ignorant of the next assignment in the sequence (different from blinding)? Possible methods can rely on centralised or "third-party" assignment (i.e., use of a central telephone randomisation system, automated assignment system, sealed containers).
5	Blinding (11a)	If done, who was blinded after assignment to interventions (for example, participants, care providers, those assessing outcomes)	Is it clear if (1) healthcare providers, (2) patients, and (3) outcome assessors are blinded to the intervention? General terms such as "double-blind" without further specifications should be avoided.
6	Outcomes and estimation (17a/b)	For the primary outcome, results for each group, and the estimated effect size and its precision (such as 95% confidence intervals)	Is the estimated effect size and its precision (such as standard deviation or 95% confidence intervals) for each treatment arm reported? When the primary outcome is binary, both the relative effect (risk ratio, relative risk) or odds ratio) and the absolute effect (risk difference) should be reported with confidence intervals.
7	Harms (19)	All-important harms or unintended effects in each group	Is the number of affected persons in each group, the severity grade (if relevant) and the absolute risk (e.g. frequency of incidence) reported? Are the number of serious, life threatening events and deaths reported? If no adverse event occurred this should be clearly stated.
8	Registration (23)	Registration number and name of trial registry	Is the registry and the registration number reported? If the trial was not registered, it should be explained why.
9	Protocol (24)	Where trial protocol can be accessed	Is it stated where the trial protocol can be assessed (e.g. published, supplementary file, repository, directly from author, confidential and therefore not available)?
10	Funding (25)	Sources of funding and other support (such as supply of drugs) and role of funders	Are (1) the funding sources, and (2) the role of the funder(s) described?

For peer review only - http://bmjopen.bmj.com/site/about/guidelines.xhtml

4.4 Outcomes

Primary outcome:

The primary outcome of this study will be the difference of the mean proportion of adequately reported items of the 10 most important and poorly reported CONSORT items between the two intervention arms.

Secondary outcomes:

Secondary outcomes will include the following:

- Mean proportion of adequate reporting of the 10 most important and poorly reported CONSORT items, considering each sub-item (see also "Assessment of outcomes") as a separate item.
- Mean proportion for each of the 10 most important and poorly reported CONSORT items separately (including also separate analysis of sub-items).
- Time from assigning an academic editor until the first decision (as communicated to the author after the first round of peer-review).
- Proportion of articles directly rejected after the first round of peer-review.
- Proportion of articles published.

Additional outcomes:

For journals where peer reviewer comments are subsequently published alongside the published article, we will examine the peer reviewer comments for any reference to CONSORT and trial reporting. We will contact those journals which do not make peer reviewer comments publicly available, to see if they still could be used for such an analyses under the condition that only anonymised data will be published.

Data collection methods:

The outcomes will be assessed independently by two (blinded or at least partially blinded; see "blinding") outcome assessors with expertise in the design and reporting of clinical trials. Any disagreement will be resolved by consensus or if necessary by consulting a third assessor. To ensure consistency between reviewers, we will first pilot the data extraction form; any disparities in the interpretation will be discussed and the data extraction form will be modified accordingly.

Adequate reporting of items will be assessed from published full-text publications adhering to the CONSORT C-short checklist (21). The following included items have sub-items which will be extracted separately:

For peer review only - http://bmjopen.bmj.com/site/about/guidelines.xhtml

- Outcomes (item 6a): (i) Define primary outcome, (ii) how it was measured, (iii) at what time point, and (iv) the analysis metric (e.g. change from baseline, final value).
- Sample size (item 7a): (i) The estimated outcomes in each group, (ii) the α (type I) error level, (iii) the statistical power (or the β (type II) error level), (iv) for continuous outcomes, the standard deviation of the measurements
- Blinding (item 11a): Is the blinding status clear for the following persons: (i) Healthcare provider, (ii) patients, and (iii) outcome assessors.
- Funding (item 25): (i) The funding source, and (ii) the role of funder in the design, conduct, analysis, and reporting.

All items will be judged as either "yes" meaning adequately reported, "no" meaning not adequately reported, or "NA" meaning that this sub-item is not applicable for this RCT. Items with different sub-items will only be judged as adequately reported if all relevant sub-items were adequately reported.

- Time from assigning an academic editor until the first decision: The day when the academic editor was assigned and the day of the first decision (e.g. major revision, minor revision, rejected) will be extracted to calculate the number of days until the first decision.
- Proportion of articles directly rejected after the first round of peer-review: Articles which were not invited for re-submission will be labelled and counted.
- Proportion of articles published: Articles which will be published will be counted and collected for data extraction.

The outcomes "time from assigning an academic editor until the first decision", "proportion of articles directly rejected after the first round of peer-review", and "proportion of articles published" will be extracted directly from editorial management software of the journal.

4.5 Participant timeline

The overview of the study schedule, including enrolment, intervention and assessments is presented in Table 2.

For peer review only - http://bmjopen.bmj.com/site/about/guidelines.xhtml

Table 2: Study schedule

	Enrolment	Allocation and intervention	Intervention	Post-intervention	
Time-point	*Studies which are sent out for peer-review*	*After first peer-reviewer accepts invitation*	*Whenever an additional peer-reviewer accepts invitation*	*First decision by journal*	*Published manuscripts*
Eligibility screen	X				
Allocation		X			
Intervention:					
C-short + usual care		X	X		
Usual care		X	X		
Assessment of trial characteristics:					
Funding source					X
Study centres (single centre or multicentre)					X
Sample size					X
Study design (e.g. parallel arm, crossover)					X
Hypothesis (e.g. superiority, non-inferiority)					X
Medical field					X
Intervention tested					X
Number of trial arms					X
Number of peer-reviewers					X
Journal which published the manuscript					X
Number of journals requesting CONSORT adherence (submission of checklist mandatory)					X
Assessment of outcomes:					
Time from assigning an academic editor until the first decision				X	
Proportion of articles directly rejected after the first round of peer-review				X	
Proportion of articles published					X
Adherence to CONSORT items and sub-items					X

For peer review only - http://bmjopen.bmj.com/site/about/guidelines.xhtml

4.6. Sample size

For the sample size calculation we hypothesise in a first scenario (Table 3) that the intervention C-Short will result in a 25% relative increase in adequate reporting compared to the control (meaning that 70% of items will be adequately reported in the intervention group and 56% in the control group). This is based on the rate of reporting of the 10 most important and poorly reported items was 0.56 (meaning that a mean of 56% of the 10 most important and poorly reported items were reported) in the control group of a previous study called WebCONSORT (38). The standard deviation (SD) in the same study was 0.23. However, we calculated our sample size to account for a slightly bigger variability in our data (SD = 0.25).To demonstrate a significant difference with a power of 90% and a type 1 error at 5% a total of 136 articles will be required in this scenario (68 per treatment arm; based on a two sided t-test).

The staff from one journal which will most likely be included (i.e. *PLoS One*) pointed out that 3 out of the 10 assessed items (i.e. item “Registration”, “Protocol”, and “Funding”) should always be implemented given their template. Assuming that this journal will recruit a high proportion, and that also other journals might update their templates, we increased the sample size in a second scenario, in which all these 3 items would have an overall adherence of 90% in the control arm (Table 3). This would entail an overall baseline adherence with the 10 CONSORT-short items of 71%. Based on a two sided t-test, a sample size of 166 (83 per treatment arm) will have a power of 80% to find a 15% relative increase (71% adherence in control group; 82% adherence in intervention group; SD = 0.25; a type 1 error at 5%).

Since the final sample size will be based on the number of articles published, rather than on the number of manuscripts randomised, eligible RCTs will be included and randomised until the number of 83 published RCTs is reached in each arm (resulting in no less than 166 articles), to avoid loss of power due to potential imbalance between arms. Recruitment will be stopped as soon as both arms reach the sample size of 83. After recruitment stop we will wait three month so that manuscripts which are still in production can be published. Manuscripts which are published after the three month period will be excluded.

For peer review only - http://bmjopen.bmj.com/site/about/guidelines.xhtml

Table 3: Assumptions for sample size calculations in two different scenarios.

Item	CONSORT item	Scenario 1. Adequate reporting as published in WebCONSORT	Scenario 2. Adapted from Scenario 1
1	Outcomes (6a)	77% (79 of 103)	77% (79 of 103)
2	Sample size (7a)	83% (85 of 103)	83% (85 of 103)
3	Sequence generation (8a)	76% (78 of 103)	76% (78 of 103)
4	Allocation concealment (9)	55% (57 of 103)	55% (57 of 103)
5	Blinding (11a)	35% (36 of 103)	35% (36 of 103)
6	Outcomes and estimation (17a	44% (45 of 103)	44% (45 of 103)
7	Harms (19)	71% (73 of 103)	71% (73 of 103)
8	Registration (23)	69% (71 of 103)	90%
9	Protocol (24)	19% (20 of 103)	90%
10	Funding (25)	34% (35 of 103)	90%
**Overall**		**56%**	**71%**

Abbreviation: CONSORT= CONsolidated Standards for Reporting Trials

4.7 Randomisation and blinding

Articles, which meet the eligibility criteria as a primary report of an RCT, for which the journal decides to send out for external peer review will be randomised into one of the two groups (allocation 1:1). The randomisation list will be created by the study-randomizer system (43) using random block sizes between 2 and 8 and stratification by journal. As soon as the first peer-reviewer accepts the invitation, the manuscript will be included and randomised to one of the two intervention arms. One of the investigators (BS) will log onto the study randomizer-system (43) entering the study identification number (ID; provided from the Journal), the study title, as well as the journal the study was submitted to. Subsequently, all additional peer-reviewers accepting the invitation to review the same manuscript will receive the same intervention (C-short plus usual practice or usual practice only) as the first peer-reviewer.

Authors will be blinded to the intervention allocation. Editors will not be actively informed about the randomisation (possible exception listed under “4.3 Interventions”). To avoid potential bias, peer reviewers and manuscript authors will not be informed of the study hypothesis, design and intervention.

Outcomes will be assessed in duplicate (see assessment of outcomes). At least one outcome assessors will be blinded. Due to restricted resources it might be possible that the investigator conducting the randomisation (BS) will be included in the data-extraction from published manuscripts.

4.7 Data management and confidentiality

For peer review only - http://bmjopen.bmj.com/site/about/guidelines.xhtml

Outcomes from publications will be assessed and extracted in duplicate. Since this information is not confidential, we will use Google Forms for data extraction from published RCTs. Data entered will be validated for completeness.

Data from the editorial manager software (e.g. Title of manuscript, first author, randomisation ID, Journal, date when manuscript was accepted by and academic editor, date when the final decision was made, final decision, number of peer-reviewers who peer reviewed the manuscript, the peer review) will be extracted, anonymised and entered in a password protected database which is saved on a server from the University of Oxford. Data will be managed and curated according to University of Oxford regulations, which includes regular back-up (on a daily basis) of the virtual drives where the data are stored.

The raw data extracted from the included manuscripts can be made openly accessible in an anonymised way (i.e. giving the included RCT a number instead of identifying them). Derived/aggregated data, including anonymised information generated from the journals' editorial manager software, will be stored and made available to the research community when the project ends (see also "8. Publication policy and access to data"). Where appropriate, the researcher who has access to the editorial manager software (BS) and anyone else who will see the identifiable data will sign a confidentially agreement with the participating journals, confirming that they will not share identifiable data with any other party. Journals such as the BMJ series state in their Company Privacy Statement that research programmes for quality improvement might be in place. Furthermore, peer reviewers for all BMJ journals receive the following statement in their invitation letter "*We are constantly trying to find ways of improving the peer review system and have an ongoing programme of research. If you do not wish your review entered into a study please let us know by emailing […] as soon as possible*."

For peer review only

4.8 Statistical methods

4.8.1 Populations of analysis

The main population for analysis will be all manuscripts randomised and accepted for publication in the participating journals. Differently from RCTs conducted with patients, where drop outs need to be carefully considered (e.g. multiple imputation of missing data), we are only interested in the reporting adherence of RCTs that are published. All outcomes will be calculated based on the main population for analysis. The secondary outcome "Time to the first decision", will additionally be calculated considering all randomised manuscripts (including the ones which were not published).

For peer review only - http://bmjopen.bmj.com/site/about/guidelines.xhtml

4.8.2 Data analysis

All quantitative variables will be described using means and standard deviations, or median and interquartile ranges in case severe departures from a normal distribution are identified. Data distribution will be inspected visually (i.e. by histograms) instead of performing formal statistical tests for normality. Categorical variables will be described using frequencies and percentages. For the primary and secondary outcomes, we will estimate the difference between means between the two groups and report them with respective 95% confidence intervals.

For peer review only

4.8.3 Analysis of primary endpoint

The primary outcome will be the difference of the mean proportion of adequately reported items of the 10 most important and poorly reported CONSORT items. If the data on the primary outcome is normally distributed then the two groups (i.e. C-short plus usual practice vs. usual practice) will be compared using an unpaired Student's t-test to compare the unadjusted mean proportion of adequate reporting. If the data is not normally distributed, comparisons will be performed using a non-parametric equivalent test (i.e. Wilcoxon-Mann-Whitney test for testing whether the population medians of the two groups are the same).

For the analyses of the primary outcomes a p-value of 0.05 (5% significance level) will be used to indicate statistical significance and treatment effect (mean difference) reported with 95% confidence intervals (or median and respective interquartile ranges, in case of asymmetric distribution). Exact p-values will be presented up to three decimal places. We anticipate there will be no missing data in this study, neither at the individual C-short items, nor at the manuscript level. This is due to the study design, which will include only the randomised manuscripts that are accepted for publication.

4.8.4 Analysis of secondary endpoints

To investigate the effect of the intervention on the secondary outcomes, mean differences with respective 95% confidence intervals will also be reported for these outcomes. If normality is not observed for any of the continuous secondary outcomes, the same strategy adopted for the primary outcome (use of a non-parametric equivalent to the Student's *t*-test) will be used.

A p-value of 0.05 will indicate statistical significance for the observed treatment effect on the secondary outcomes. Exact p-values will be presented up to three decimal places. Similarly to the primary outcome, we anticipate there will be no missing data for any of the secondary

For peer review only - http://bmjopen.bmj.com/site/about/guidelines.xhtml

outcomes, as we will have access to the Editorial Management system of the included journals, where all relevant information is automatically reported.

4.8.5 Pre-specified subgroup analysis

No formal subgroup comparative analysis is planned for the primary or secondary outcomes. However, the effect of the intervention on the primary outcome within subgroups, will be presented using forest plots to visually examine whether it differs according to some variables, such as: (1) Journals that actively implement the CONSORT Statement (defined as requiring authors to submit a completed CONSORT checklist alongside their manuscript) vs. journals that are not actively implementing the CONSORT Statement; (2) sample size (n < 100 vs. n ≥ 100); and (3) impact factor (<5, 5.1-10; >10) as there is evidence that higher impact factor as well as higher sample size are associated with higher adherence to reporting guidelines (44). These analyses will be exploratory, with the aim of supporting new hypothesis generation, rather than conclusive.

5 Legal and general logistics

5.1. Organisation of study

5.1.1 Coordinating centre

The coordinating centre's, will be the Centre for Statistics in Medicine at the University of Oxford under the responsibilities of Dr Sally Hopewell and Dr Benjamin Speich.

The coordinating centre's will ensure the following missions:

- Training of the staff
- Implementation of quality control
- Logical controls of data
- Follow-up on requests for correction/validation
- Statistical analysis
- Archiving of data

5.1.2 Scientific committee

The scientific committee is composed of:

- Prof Isabelle Boutron: Centre D'Épidémiologie Clinique Hôtel-Dieu, Paris Descartes University, France
- Prof Matthias Briel, University of Basel, Switzerland
- Associate Prof Sally Hopewell: Centre for Statistics in Medicine, University of Oxford, UK

For peer review only - http://bmjopen.bmj.com/site/about/guidelines.xhtml

- Prof David Moher: Centre for Journalology, Clinical Epidemiology Program, Ottawa Hospital Research Institute, Canada
- Prof Philippe Ravaud: Centre d'Épidémiologie Clinique Hôtel-Dieu, Paris Descartes University, France
- Dr Benjamin Speich, Centre for Statistics in Medicine, University of Oxford, UK
- Dr. Michael M Schlussel, Centre for Statistics in Medicine, University of Oxford, UK
- Dr Sara Schroter, The BMJ, London, UK

The scientific committee is in charge of:

- Participating in the elaboration of the protocol
- Defining and validating the additional short explanation for each CONSORT item.
- Following the evolution of the committed study
- Publishing the results of this study

5.2. Regulatory aspects

Ethical approval for this study will be sought from the Central University Research Ethics Committee (CUREC) of the University of Oxford. Any amendments in the conduct of the study, collection of outcomes or analysis will be reported to the CUREC. The tested intervention has the goal to improve the quality of published journals (i.e. the adherence to CONSORT) and could also be implemented as usual practice without testing at the journal level. In agreement with another study, testing a similar intervention (45), we think that it is ethical to conduct this study without obtaining written consent. The main reason for this procedure are the following:

- Informing the authors and peer-reviewers would make it impossible to measure the effect of our intervention. In short, informing peer-reviewers and authors would create an artificial context which would not be comparable any more to the "real world context". Authors and peer-reviewers would most likely be much more aware of CONSORT if they received information about the study. Furthermore, being aware to participate in a study could strongly influence the natural behaviour of peer-reviewers (e.g. putting more effort into reviewing a manuscript than under "real world conditions") but also of authors.
- The intervention does not pose any risk of harms for authors and peer-reviewers.
- The intervention is not a medical intervention but rather tries to improve the research quality and journal processes.
- Several journal series (e.g. BMJ series) have Company Privacy Statements in place which clearly mention that research programmes might be in place for quality improvement.

For peer review only - http://bmjopen.bmj.com/site/about/guidelines.xhtml

- The intervention could be part of the routine at any Journal without previous assessment of its efficacy.
- No data which identifies participating manuscripts will be published.

6 Publication policy and access to data

The results from this study will be published in a peer-reviewed journal irrespective of the study results. Authorship to publications will be granted according to the rules of the International Committee of Medical Journal Editors (ICMJE). We plan to publish the full anonymised dataset as a supplementary file together with the main publication.

For peer review only

For peer review only - http://bmjopen.bmj.com/site/about/guidelines.xhtml

References

1. Duley L, Antman K, Arena J, Avezum A, Blumenthal M, Bosch J, et al. Specific barriers to the conduct of randomized trials. Clin Trials. 2008;5(1):40-8.
2. Collins R, MacMahon S. Reliable assessment of the effects of treatment on mortality and major morbidity, I: clinical trials. Lancet. 2001;357(9253):373-80.
3. Chalmers I. Unbiased, relevant, and reliable assessments in health care: important progress during the past century, but plenty of scope for doing better. BMJ. 1998;317(7167):1167-8.
4. Collier R. Rapidly rising clinical trial costs worry researchers. CMAJ. 2009;180(3):277-8.
5. Eisenstein EL. Commentary on Sertkaya et al. and Larson et al. Clin Trials. 2016;13(2):137-9.
6. Sertkaya A, Wong HH, Jessup A, Beleche T. Key cost drivers of pharmaceutical clinical trials in the United States. Clin Trials. 2016;13(2):117-26.
7. Glasziou P, Altman DG, Bossuyt P, Boutron I, Clarke M, Julious S, et al. Reducing waste from incomplete or unusable reports of biomedical research. Lancet. 2014;383(9913):267-76.
8. Chan AW, Song F, Vickers A, Jefferson T, Dickersin K, Gotzsche PC, et al. Increasing value and reducing waste: addressing inaccessible research. Lancet. 2014;383(9913):257-66.
9. Chan AW, Tetzlaff JM, Altman DG, Dickersin K, Moher D. SPIRIT 2013: new guidance for content of clinical trial protocols. Lancet. 2013;381(9861):91-2.
10. Chan AW, Tetzlaff JM, Altman DG, Laupacis A, Gotzsche PC, Krleza-Jeric K, et al. SPIRIT 2013 statement: defining standard protocol items for clinical trials. Ann Intern Med. 2013;158(3):200-7.
11. Chan AW, Tetzlaff JM, Gotzsche PC, Altman DG, Mann H, Berlin JA, et al. SPIRIT 2013 explanation and elaboration: guidance for protocols of clinical trials. BMJ. 2013;346:e7586.
12. Lund H, Brunnhuber K, Juhl C, Robinson K, Leenaars M, Dorch BF, et al. Towards evidence based research. BMJ. 2016;355:i5440.
13. Kasenda B, von Elm E, You J, Blumle A, Tomonaga Y, Saccilotto R, et al. Prevalence, characteristics, and publication of discontinued randomized trials. JAMA. 2014;311(10):1045-51.
14. Alturki R, Schandelmaier S, Olu KK, von Niederhausern B, Agarwal A, Frei R, et al. Premature trial discontinuation often not accurately reflected in registries: comparison of registry records with publications. J Clin Epidemiol. 2017;81:56-63.
15. Altman DG, Simera I, Hoey J, Moher D, Schulz K. EQUATOR: reporting guidelines for health research. Lancet. 2008;371(9619):1149-50.
16. Simera I. EQUATOR Network collates resources for good research. BMJ. 2008;337:a2471.
17. Simera I, Altman DG. Writing a research article that is "fit for purpose": EQUATOR Network and reporting guidelines. Evid Based Med. 2009;14(5):132-4.
18. Simera I, Altman DG, Moher D, Schulz KF, Hoey J. Guidelines for reporting health research: the EQUATOR network's survey of guideline authors. PLoS Med. 2008;5(6):e139.
19. Simera I, Moher D, Hirst A, Hoey J, Schulz KF, Altman DG. Transparent and accurate reporting increases reliability, utility, and impact of your research: reporting guidelines and the EQUATOR Network. BMC Med. 2010;8:24.
20. Simera I, Moher D, Hoey J, Schulz KF, Altman DG. The EQUATOR Network and reporting guidelines: Helping to achieve high standards in reporting health research studies. Maturitas. 2009;63(1):4-6.
21. Moher D, Hopewell S, Schulz KF, Montori V, Gotzsche PC, Devereaux PJ, et al. CONSORT 2010 explanation and elaboration: updated guidelines for reporting parallel group randomised trials. BMJ. 2010;340:c869.

For peer review only - http://bmjopen.bmj.com/site/about/guidelines.xhtml

22. Schulz KF, Altman DG, Moher D, Group C. CONSORT 2010 statement: updated guidelines for reporting parallel group randomised trials. BMJ. 2010;340:c332.
23. Schulz KF, Altman DG, Moher D, Group C. CONSORT 2010 Statement: updated guidelines for reporting parallel group randomised trials. BMC Med. 2010;8:18.
24. CONSORT Transparent Reporting of Trials. Endorsers. Journals and Organizations. http://www.consort-statement.org/about-consort/endorsers [accessed: 27 February 2019]
25. Chan AW, Hrobjartsson A, Haahr MT, Gotzsche PC, Altman DG. Empirical evidence for selective reporting of outcomes in randomized trials: comparison of protocols to published articles. JAMA. 2004;291(20):2457-65.
26. Chalmers I, Glasziou P. Avoidable waste in the production and reporting of research evidence. Lancet. 2009;374(9683):86-9.
27. Ioannidis JP, Lau J. Completeness of safety reporting in randomized trials: an evaluation of 7 medical areas. JAMA. 2001;285(4):437-43.
28. Glasziou P, Meats E, Heneghan C, Shepperd S. What is missing from descriptions of treatment in trials and reviews? BMJ. 2008;336(7659):1472-4.
29. Boutron I, Dutton S, Ravaud P, Altman DG. Reporting and interpretation of randomized controlled trials with statistically nonsignificant results for primary outcomes. JAMA. 2010;303(20):2058-64.
30. Hopewell S, Dutton S, Yu LM, Chan AW, Altman DG. The quality of reports of randomised trials in 2000 and 2006: comparative study of articles indexed in PubMed. BMJ. 2010;340:c723.
31. Turner L, Shamseer L, Altman DG, Schulz KF, Moher D. Does use of the CONSORT Statement impact the completeness of reporting of randomised controlled trials published in medical journals? A Cochrane review. Syst Rev. 2012;1:60.
32. Odutayo A, Emdin CA, Hsiao AJ, Shakir M, Copsey B, Dutton S, et al. Association between trial registration and positive study findings: cross sectional study (Epidemiological Study of Randomized Trials-ESORT). BMJ. 2017;356:j917.
33. Chan AW, Krleza-Jeric K, Schmid I, Altman DG. Outcome reporting bias in randomized trials funded by the Canadian Institutes of Health Research. CMAJ. 2004;171(7):735-40.
34. Glass KC. Toward a duty to report clinical trials accurately: the clinical alert and beyond. J Law Med Ethics. 1994;22(4):327-38.
35. Shamseer L, Hopewell S, Altman DG, Moher D, Schulz KF. Update on the endorsement of CONSORT by high impact factor journals: a survey of journal "Instructions to Authors" in 2014. Trials. 2016;17(1):301.
36. Hopewell S, Altman DG, Moher D, Schulz KF. Endorsement of the CONSORT Statement by high impact factor medical journals: a survey of journal editors and journal 'Instructions to Authors'. Trials. 2008;9:20.
37. Hopewell S, Ravaud P, Baron G, Boutron I. Effect of editors' implementation of CONSORT guidelines on the reporting of abstracts in high impact medical journals: interrupted time series analysis. BMJ. 2012;344:e4178.
38. Hopewell S, Boutron I, Altman DG, Barbour G, Moher D, Montori V, et al. Impact of a web-based tool (WebCONSORT) to improve the reporting of randomised trials: results of a randomised controlled trial. BMC Med. 2016;14(1):199.
39. Blanco D, Kirkham JJ, Altman DG, Moher D, Boutron I, Cobo E. Interventions to improve adherence to reporting guidelines in health research: a scoping review protocol. BMJ Open. 2017;7(11):e017551.
40. Hopewell S, Collins GS, Boutron I, Yu LM, Cook J, Shanyinde M, et al. Impact of peer review on reports of randomised trials published in open peer review journals: retrospective before and after study. BMJ. 2014;349:g4145.
41. Cobo E, Selva-O'Callagham A, Ribera JM, Cardellach F, Dominguez R, Vilardell M. Statistical reviewers improve reporting in biomedical articles: a randomized trial. PLoS One. 2007;2(3):e332.

For peer review only - http://bmjopen.bmj.com/site/about/guidelines.xhtml

42. Barnes C, Boutron I, Giraudeau B, Porcher R, Altman DG, Ravaud P. Impact of an online writing aid tool for writing a randomized trial report: the COBWEB (Consort-based WEB tool) randomized controlled trial. BMC Med. 2015;13:221.
43. Study Randomizer. https://studyrandomizer.com/ [accessed: 23. January 2019].
44. Jin Y, Sanger N, Shams I, Luo C, Shahid H, Li G, et al. Does the medical literature remain inadequately described despite having reporting guidelines for 21 years? - A systematic review of reviews: an update. Journal of multidisciplinary healthcare. 2018;11:495-510.
45. Hair K, Macleod MR, Sena ES. A randomised controlled trial of an Intervention to Improve Compliance with the ARRIVE guidelines (IICARus). bioRxiv. 2018.

For peer review only

Appendix

Appendix 1: Example of the email which will be sent out in the intervention arm (C-Short). The exact wording might be slightly adapted according to the journal preferences.

Dear **Title, Name*,

We thank you for accepting to peer-review a manuscript for **journal name**. As we are trying to improve the reporting for randomised controlled trials according to the CONSORT guidelines, we would like to ask if you could check whether the following most important and poorly reported items are adequately implemented as indicated in the *table below/attached table*.

Item	Section	CONSORT item	Short explanation
1	Outcomes (6a)	Completely defined pre-specified primary outcome measure, including how and when they were assessed	Is it clear (1) what the primary outcome is (usually the one used in the sample size calculation), (2) how it was measured (if relevant; e.g. which score used), (3) at what time point, and (4) what the analysis metric was (e.g. change from baseline, final value)?
2	Sample size (7a)	How sample size was determined	Is there a clear description of how the sample size was determined, including (1) the estimated outcomes in each group; (2) the α (type I) error level; (3) the statistical power (or the β (type II) error level); and (4) for continuous outcomes, the standard deviation of the measurements?
3	Sequence generation (8a)	Method used to generate random allocation sequence	Does the description make it clear if the "assigned intervention is determined by a chance process and cannot be predicted"?
4	Allocation concealment (9)	Mechanism used to implement random allocation sequence (such as sequentially numbered containers), describing any steps taken to conceal the sequence until interventions were assigned	Is it clear how the care provider enrolling participants was made ignorant of the next assignment in the sequence (different from blinding)? Possible methods can rely on centralised or "third-party" assignment (i.e., use of a central telephone randomisation system, automated assignment system, sealed containers).
5	Blinding (11a)	If done, who was blinded after assignment to interventions (for example, participants, care providers, those assessing outcomes)	Is it clear if (1) healthcare providers, (2) patients, and (3) outcome assessors are blinded to the intervention? General terms such as "double-blind" without further specifications should be avoided.
6	Outcomes and estimation (17a/b)	For the primary outcome, results for each group, and the estimated effect size and its precision (such as 95% confidence intervals)	Is the estimated effect size and its precision (such as standard deviation or 95% confidence intervals) for each treatment arm reported? When the primary outcome is binary, both the relative effect (risk ratio, relative risk) or odds ratio) and the absolute effect (risk difference) should be reported with confidence intervals.
7	Harms (19)	All-important harms or unintended effects in each group	Is the number of affected persons in each group, the severity grade (if relevant) and the absolute risk (e.g. frequency of incidence) reported? Are the number of serious, life threatening events and deaths reported? If no adverse event occurred this should be clearly stated.
8	Registration (23)	Registration number and name of trial registry	Is the registry and the registration number reported? If the trial was not registered, it should be explained why.
9	Protocol (24)	Where trial protocol can be accessed	Is it stated where the trial protocol can be assessed (e.g. published, supplementary file, repository, directly from author, confidential and therefore not available)?
10	Funding (25)	Sources of funding and other support (such as supply of drugs) and role of funders	Are (1) the funding sources, and (2) the role of funder(s) described?

Your efforts are highly appreciated.

Kind regards,

journal name-Team

For peer review only - http://bmjopen.bmj.com/site/about/guidelines.xhtml

Appendix 3: WHO Trial Registration Data Set (Version 1.3.1)

Statement was filled out on the 01. October 2019.

1. Primary Registry and Trial Identifying Number

This trial was denied registration on ClinicalTrials.gov as the study is not a clinical study that assesses a health outcome in human subjects. Instead we registered the trial on the Open Science Framework (https://osf.io/c4hn8).

2. Date of Registration in Primary Registry

21. June 2019

3. Secondary Identifying Numbers

Not applicable

4. Source(s) of Monetary or Material Support

No specific funding was acquired for this study. Benjamin Speich is supported by an Advanced Postdoc.Mobility grant from the Swiss National Science Foundation (P300PB_177933). David Moher is supported by a University Research Chair, Ottawa. Michael M Schlussel is funded by Cancer Research UK. The funders had no role in designing the study and will also have no role in conducting the study, or analysing and reporting study results.

5. Primary Sponsor

Sponsor:	University of Oxford
Principal Investigator/Sponsor Investigator:	Benjamin Speich, PhD<br>Postdoctoral Researcher<br>Centre for Statistics in Medicine (CSM)<br>Nuffield Department of Orthopaedics, Rheumatology and Musculoskeletal Sciences (NDORMS)<br>University of Oxford<br>Windmill Road<br>Oxford OX3 7LD<br>Tel: +44 1865 737904<br><br>Email: benjamin.speich@ndorms.ox.ac.uk

6. Secondary Sponsor(s)

Not applicable

For peer review only - http://bmjopen.bmj.com/site/about/guidelines.xhtml

7. Contact for Public Queries

Dr. Benjamin Speich

Tel: +44 1865 737904

Email: benjamin.speich@ndorms.ox.ac.uk

8. Contact for Scientific Queries

Sponsor:	University of Oxford
Principal Investigator/Sponsor Investigator:	Benjamin Speich, PhD<br>Postdoctoral Researcher<br>Centre for Statistics in Medicine (CSM)<br>Nuffield Department of Orthopaedics, Rheumatology and Musculoskeletal Sciences (NDORMS)<br>University of Oxford<br>Windmill Road<br>Oxford OX3 7LD<br>Tel: +44 1865 737904<br><br>Email: benjamin.speich@ndorms.ox.ac.uk

9. Public Title

Impact of checklists to improve the reporting of randomised controlled trials published in biomedical journals

10. Scientific Title

Impact of a short version of the CONSORT checklist for peer reviewers to improve the reporting of randomised controlled trials published in biomedical journals: a randomised controlled trial

Running title: CONSORT for Peer Review (CONSORT-PR)

Study identifier: CONSORT-PR

11. Countries of Recruitment

Multinational (Centres are Biomedical journals)

12. Health Condition(s) or Problem(s) Studied

Reporting in published randomised controlled trials

13. Intervention(s)

Control group: Usual practice

For peer review only

After accepting to review a manuscript, peer reviewers will receive the automated, journal specific standard email with general information as per each journal's usual practice (e.g. where to access the manuscript, date the peer review report is due).

Intervention group: C-short plus usual practice

After accepting to review a manuscript, peer reviewers will receive the automated, journal specific standard email with general information (identical to control group). In addition, peer reviewers will receive an additional email from the editorial office that includes a short version of the CONSORT checklist (C-short) together with a brief explanation of the items either as a table within the email or as an attachment. Peer reviewers will be asked to check whether the items in the C-short checklist are addressed in the manuscript and to request authors to include these items if they are not adequately reported.

14. Key Inclusion and Exclusion Criteria

The population will be defined on two levels: included journals and included manuscripts.

Inclusion criteria for journals:

Included journals must: i) endorse the CONSORT Statement by mentioning it in the journals' Instruction to Authors; ii) have published primary results of at least five RCTs in 2017 (identified using a PubMed search).

Inclusion criteria for manuscripts

- All new manuscript submissions reporting the primary results of RCTs, which the journal editor has decided to send out for external peer review. Since the 10 chosen CONSORT checklist items (C-short) are applicable to different study designs, we will include all manuscripts reporting the primary results of RCTs regardless of study design (e.g. parallel group trial, cluster trial, superiority trial, non-inferiority/equivalence trials).

Exclusion criteria for manuscripts

- Manuscripts clearly presenting secondary trial results, additional time points, economic analyses, or any other analyses.
- Manuscripts which are clearly labelled as a pilot or feasibility study or animal studies.
- Manuscripts not sent for peer review.

15. Study Type

This study is a multicentre RCT with submitted manuscripts as the unit of randomisation (allocation ratio 1:1).

For peer review only - http://bmjopen.bmj.com/site/about/guidelines.xhtml

16. Date of First Enrollment

22. July 2019

17. Sample Size

166 Since the final sample size will be based on the number of articles published, rather than on the number of manuscripts randomised, eligible manuscripts will be randomised until 83 articles are published in each arm (resulting in no less than 166 articles), to avoid loss of power due to potential imbalance between arms.

18. Recruitment Status

Recruiting

19. Primary Outcome(s)

- The primary outcome of this study will be the difference in the mean proportion of adequately reported C-short items in published articles between the two groups.

20. Key Secondary Outcomes

- Mean proportion of adequately reported C-short items in published articles considering each item separately.
- Difference in mean proportion of adequately reported C-short items in published articles considering each sub-item (see "Assessment of outcomes") as a separate item.
- Time from assigning an editor to the first decision (as communicated to the author after the first round of peer-review).
- Proportion of manuscripts rejected after the first round of peer review.
- Proportion of manuscripts that will be published in the journal under study.

21. Ethics Review

Ethical approval has been obtained from the Medical Sciences Interdivisional Research Ethics Committee of the University of Oxford (R62779/RE001).

22. Completion date

We expect that recruitment will be finished in summer 2021.

23. Summary Results

Not applicable

24. IPD sharing statement

We plan to make the anonymised dataset, including the data from the published articles, available as a supplementary file of the main publication.

For peer review only - http://bmjopen.bmj.com/site/about/guidelines.xhtml

Impact of a short form of the CONSORT checklist for peer reviewers to improve the reporting of randomised controlled trials published in biomedical journals: study protocol for a randomised controlled trial

Benjamin Speich[1,2,*], Sara Schroter[3], Matthias Briel[2,4], David Moher[5], Michael M Schlussel[1], Philippe Ravaud[6,7], Isabelle Boutron[6,7], Sally Hopewell[1]

SPIRIT — STANDARD PROTOCOL ITEMS: RECOMMENDATIONS FOR INTERVENTIONAL TRIALS

SPIRIT 2013 Checklist: Recommended items to address in a clinical trial protocol and related documents*

Section/item	Item No	Description	Addressed on page number
**Administrative information**			
Title	1	Descriptive title identifying the study design, population, interventions, and, if applicable, trial acronym	1____________
Trial registration	2a	Trial identifier and registry name. If not yet registered, name of intended registry	3______
	2b	All items from the World Health Organization Trial Registration Data Set	Appendix 3
Protocol version	3	Date and version identifier	Appendix 1
Funding	4	Sources and types of financial, material, and other support	24____________
Roles and responsibilities	5a	Names, affiliations, and roles of protocol contributors	1 and 24-25__
	5b	Name and contact information for the trial sponsor	1 and Appendix 1_

For peer review only - http://bmjopen.bmj.com/site/about/guidelines.xhtml

	5c	Role of study sponsor and funders, if any, in study design; collection, management, analysis, and interpretation of data; writing of the report; and the decision to submit the report for publication, including whether they will have ultimate authority over any of these activities	24-25_______
	5d	Composition, roles, and responsibilities of the coordinating centre, steering committee, endpoint adjudication committee, data management team, and other individuals or groups overseeing the trial, if applicable (see Item 21a for data monitoring committee)	24-25_______
**Introduction**			
Background and rationale	6a	Description of research question and justification for undertaking the trial, including summary of relevant studies (published and unpublished) examining benefits and harms for each intervention	5-7__________
	6b	Explanation for choice of comparators	5-7 (comparator, usual practice)______
Objectives	7	Specific objectives or hypotheses	7
Trial design	8	Description of trial design including type of trial (eg, parallel group, crossover, factorial, single group), allocation ratio, and framework (eg, superiority, equivalence, noninferiority, exploratory)	7____________
**Methods: Participants, interventions, and outcomes**			
Study setting	9	Description of study settings (eg, community clinic, academic hospital) and list of countries where data will be collected. Reference to where list of study sites can be obtained	7-9__________
Eligibility criteria	10	Inclusion and exclusion criteria for participants. If applicable, eligibility criteria for study centres and individuals who will perform the interventions (eg, surgeons, psychotherapists)	7-9_
Interventions	11a	Interventions for each group with sufficient detail to allow replication, including how and when they will be administered	9-10, Table 1, Appendix_____
	11b	Criteria for discontinuing or modifying allocated interventions for a given trial participant (eg, drug dose change in response to harms, participant request, or improving/worsening disease)	NA (one time intervention)

For peer review only

	11c	Strategies to improve adherence to intervention protocols, and any procedures for monitoring adherence (eg, drug tablet return, laboratory tests)	NA (one time intervention)
	11d	Relevant concomitant care and interventions that are permitted or prohibited during the trial	NA_______
Outcomes	12	Primary, secondary, and other outcomes, including the specific measurement variable (eg, systolic blood pressure), analysis metric (eg, change from baseline, final value, time to event), method of aggregation (eg, median, proportion), and time point for each outcome. Explanation of the clinical relevance of chosen efficacy and harm outcomes is strongly recommended	10-12______
Participant timeline	13	Time schedule of enrolment, interventions (including any run-ins and washouts), assessments, and visits for participants. A schematic diagram is highly recommended (see Figure)	Figure 1 and Table Table 2________
Sample size	14	Estimated number of participants needed to achieve study objectives and how it was determined, including clinical and statistical assumptions supporting any sample size calculations	13-14__________
Recruitment	15	Strategies for achieving adequate participant enrolment to reach target sample size	7-8, 13-14___

Methods: Assignment of interventions (for controlled trials)

Allocation:

Sequence generation	16a	Method of generating the allocation sequence (eg, computer-generated random numbers), and list of any factors for stratification. To reduce predictability of a random sequence, details of any planned restriction (eg, blocking) should be provided in a separate document that is unavailable to those who enrol participants or assign interventions	14________
Allocation concealment mechanism	16b	Mechanism of implementing the allocation sequence (eg, central telephone; sequentially numbered, opaque, sealed envelopes), describing any steps to conceal the sequence until interventions are assigned	14_________
Implementation	16c	Who will generate the allocation sequence, who will enrol participants, and who will assign participants to interventions	14_________
Blinding (masking)	17a	Who will be blinded after assignment to interventions (eg, trial participants, care providers, outcome assessors, data analysts), and how	14_____________

	17b	If blinded, circumstances under which unblinding is permissible, and procedure for revealing a participant's allocated intervention during the trial	NA__________
**Methods: Data collection, management, and analysis**			
Data collection methods	18a	Plans for assessment and collection of outcome, baseline, and other trial data, including any related processes to promote data quality (eg, duplicate measurements, training of assessors) and a description of study instruments (eg, questionnaires, laboratory tests) along with their reliability and validity, if known. Reference to where data collection forms can be found, if not in the protocol	11-12__
	18b	Plans to promote participant retention and complete follow-up, including list of any outcome data to be collected for participants who discontinue or deviate from intervention protocols	15 (no missing data expected)__
Data management	19	Plans for data entry, coding, security, and storage, including any related processes to promote data quality (eg, double data entry; range checks for data values). Reference to where details of data management procedures can be found, if not in the protocol	16-17__
Statistical methods	20a	Statistical methods for analysing primary and secondary outcomes. Reference to where other details of the statistical analysis plan can be found, if not in the protocol	14-16________
	20b	Methods for any additional analyses (eg, subgroup and adjusted analyses)	16_____
	20c	Definition of analysis population relating to protocol non-adherence (eg, as randomised analysis), and any statistical methods to handle missing data (eg, multiple imputation)	15 (no missing data expected)___
**Methods: Monitoring**			
Data monitoring	21a	Composition of data monitoring committee (DMC); summary of its role and reporting structure; statement of whether it is independent from the sponsor and competing interests; and reference to where further details about its charter can be found, if not in the protocol. Alternatively, an explanation of why a DMC is not needed	24-25_________
	21b	Description of any interim analyses and stopping guidelines, including who will have access to these interim results and make the final decision to terminate the trial	15___________
Harms	22	Plans for collecting, assessing, reporting, and managing solicited and spontaneously reported adverse events and other unintended effects of trial interventions or trial conduct	NA__

For peer review only - http://bmjopen.bmj.com/site/about/guidelines.xhtml

Auditing	23	Frequency and procedures for auditing trial conduct, if any, and whether the process will be independent from investigators and the sponsor	24-25__________
**Ethics and dissemination**			
Research ethics approval	24	Plans for seeking research ethics committee/institutional review board (REC/IRB) approval	20_____________
Protocol amendments	25	Plans for communicating important protocol modifications (eg, changes to eligibility criteria, outcomes, analyses) to relevant parties (eg, investigators, REC/IRBs, trial participants, trial registries, journals, regulators)	20_
Consent or assent	26a	Who will obtain informed consent or assent from potential trial participants or authorised surrogates, and how (see Item 32)	Appendix 2_____
	26b	Additional consent provisions for collection and use of participant data and biological specimens in ancillary studies, if applicable	NA__________
Confidentiality	27	How personal information about potential and enrolled participants will be collected, shared, and maintained in order to protect confidentiality before, during, and after the trial	17_
Declaration of interests	28	Financial and other competing interests for principal investigators for the overall trial and each study site	24_____________
Access to data	29	Statement of who will have access to the final trial dataset, and disclosure of contractual agreements that limit such access for investigators	20__
Ancillary and post-trial care	30	Provisions, if any, for ancillary and post-trial care, and for compensation to those who suffer harm from trial participation	NA__
Dissemination policy	31a	Plans for investigators and sponsor to communicate trial results to participants, healthcare professionals, the public, and other relevant groups (eg, via publication, reporting in results databases, or other data sharing arrangements), including any publication restrictions	20_________
	31b	Authorship eligibility guidelines and any intended use of professional writers	20____________

For peer review only - http://bmjopen.bmj.com/site/about/guidelines.xhtml

	31c	Plans, if any, for granting public access to the full protocol, participant-level dataset, and statistical code	Intend to publish in BMJ open (protocol), dataset: page 20
**Appendices**			
Informed consent materials	32	Model consent form and other related documentation given to participants and authorised surrogates	Appendix 2 (no consent)_
Biological specimens	33	Plans for collection, laboratory evaluation, and storage of biological specimens for genetic or molecular analysis in the current trial and for future use in ancillary studies, if applicable	NA_________

*It is strongly recommended that this checklist be read in conjunction with the SPIRIT 2013 Explanation & Elaboration for important clarification on the items. Amendments to the protocol should be tracked and dated. The SPIRIT checklist is copyrighted by the SPIRIT Group under the Creative Commons "Attribution-NonCommercial-NoDerivs 3.0 Unported" license.

For peer review only
